# Development and mechanistic investigation of the manganese(iii) salen-catalyzed dehydrogenation of alcohols†
†Electronic supplementary information (ESI) available: Experimental procedures, kinetic and compound characterization data, copies of ^1^H and ^13^C NMR spectra as well as coordinates and energies from DFT calculations. See DOI: 10.1039/c8sc03969k


**DOI:** 10.1039/c8sc03969k

**Published:** 2018-11-13

**Authors:** Simone V. Samuelsen, Carola Santilli, Mårten S. G. Ahlquist, Robert Madsen

**Affiliations:** a Department of Chemistry , Technical University of Denmark , 2800 Kgs. Lyngby , Denmark . Email: rm@kemi.dtu.dk; b Department of Theoretical Chemistry & Biology , School of Engineering Sciences in Chemistry Biotechnology and Health , KTH Royal Institute of Technology , 10691 Stockholm , Sweden

## Abstract

The first example of a manganese(iii) catalyst for the acceptorless dehydrogenation of alcohols is presented. *N*,*N′*-Bis(salicylidene)-1,2-cyclohexanediaminomanganese(iii) chloride (**2**) has been shown to catalyze the direct synthesis of imines from a variety of alcohols and amines with the liberation of hydrogen gas. The mechanism has been investigated experimentally with labelled substrates and theoretically with DFT calculations. The results indicate a metal–ligand bifunctional pathway in which both imine groups in the salen ligand are first reduced to form a manganese(iii) amido complex as the catalytically active species. Dehydrogenation of the alcohol then takes place by a stepwise outer-sphere hydrogen transfer generating a manganese(iii) salan hydride from which hydrogen gas is released.

## Abstract 2

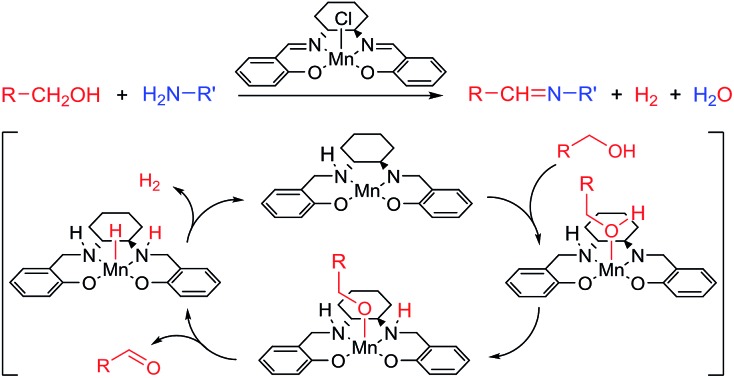
Manganese(iii) salen has been developed as a new catalytic motif for alcohol dehydrogenation and the mechanism has been elucidated.

## Introduction

**Fig. 1:**
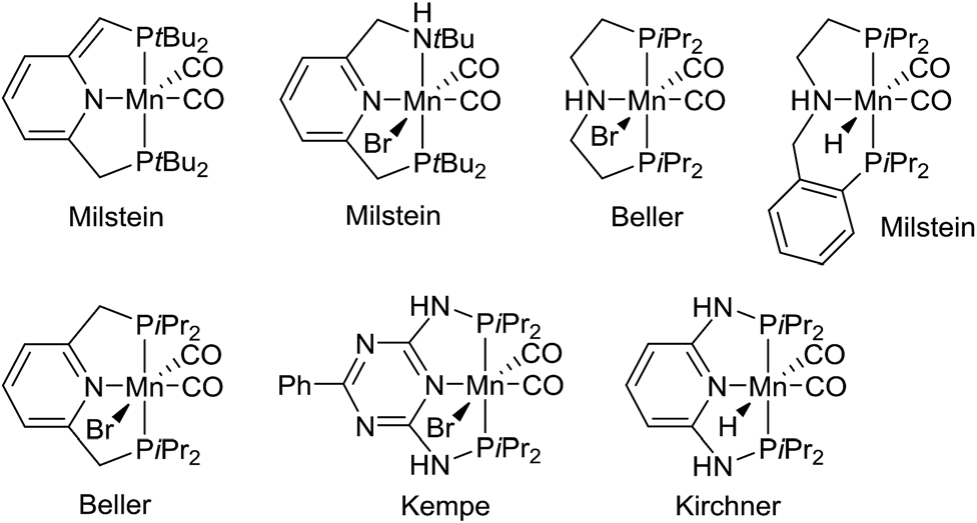
Manganese(i) complexes for alcohol dehydrogenation.^3^,^4^

Metal-catalyzed dehydrogenation of alcohols gives rise to aldehydes and ketones, which can be further transformed into imines, amides, esters, carboxylic acids and various heterocycles in the same pot.^1^ The acceptorless dehydrogenation constitutes an attractive synthetic protocol since it does not require any stoichiometric oxidants and only releases hydrogen gas as a co-product. The dehydrogenative transformations are usually catalyzed by complexes of the platinum-group metals such as ruthenium and iridium.^1^ Recently, however, non-noble metal complexes based on iron, cobalt and manganese have also been shown to catalyze alcohol dehydrogenations.^2^ Especially manganese-catalyzed dehydrogenations have been a hot research area since the first catalyst was introduced in 2016.^3^ Since then several research groups have presented different manganese complexes for preparing various functional groups and heterocyclic frameworks (Fig. 1).^4^ These complexes have also been used to catalyze the hydrogenation of carbonyl compounds.^5^ The significance of the discoveries is exemplified by the large number of works published on this topic over the past two years^3^–^6^ including several reviews.2a–d

Notably, the developed complexes are all manganese(i) compounds with CO ligands and a pincer ligand. The electron-withdrawing CO ligand is necessary for stabilizing the low oxidation state of manganese. The mechanism for the dehydrogenation with these complexes is believed to involve a catalytic cycle with different manganese(i) species^3^,4b,e,5b and no catalytic activity is observed without the CO ligands or with the corresponding manganese(ii) dihalide complexes.5*d* Although manganese is an Earth-abundant and cheap metal, manganese(i) complexes are not inexpensive since they are prepared in several steps from Mn_2_(CO)_10_. This carbonyl complex is quite expensive due to its difficult preparation by a carbonylation reaction.^7^ As a result, there is a need for identifying a new and more abundantly available class of manganese complexes for alcohol dehydrogenations. Especially, it would be attractive to catalyze the dehydrogenations by higher valent complexes where stabilization by CO ligands is not necessary.

This prompted the question whether manganese(iii) complexes would be able to catalyze the same acceptorless alcohol dehydrogenations? Manganese(iii) complexes such as the Jacobsen's catalyst^8^ are widely known for catalyzing oxidation reactions in the presence of a stoichiometric oxidant. This includes epoxidation of olefins, oxidation of alcohols as well as hydroxylation and halogenation of alkanes.^9^ The reactions proceed through catalytic cycles with different manganese(iii), (iv) and (v) species.^9^

Herein, we describe the first example of an acceptorless alcohol dehydrogenation catalyzed by a manganese(iii) complex. An easily available manganese(iii) salen catalyst has been shown to liberate hydrogen gas from alcohols and the transformation has been applied to the synthesis of imines from alcohols and amines.^10^ The mechanism has been investigated by experimental and theoretical methods which indicates a metal–ligand bifunctional pathway^11^ for the removal of hydrogen gas from the alcohol.

## Results and discussion

**Fig. 2:**
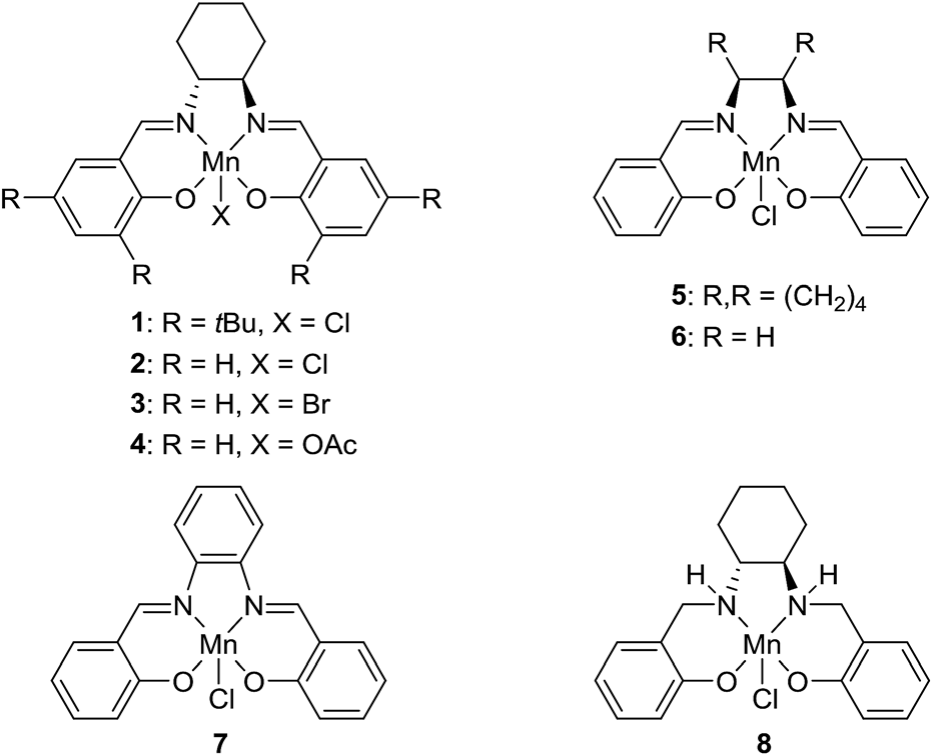
Manganese(iii) salen/salan complexes used in the investigation.

The transformation was discovered while attempting to develop a more convenient *in situ*-formed catalyst system (Table 1). Benzyl alcohol was treated with an equimolar amount of cyclohexylamine in the presence of Mn_2_(CO)_10_ and different ligands. The most promising result was obtained with *N*,*N′*-bis(salicylidene)ethylenediamine (H_2_salen) as the ligand (entry 1). Since this is a common ligand for manganese(iii) complexes, an experiment was also performed with the Jacobsen's catalyst (**1**, Fig. 2). Interestingly, this increased the yield to 79% with some unreacted benzyl alcohol remaining (entry 2).^12^ This observation shows that the acceptorless dehydrogenation of alcohols can also be catalyzed by manganese(iii) complexes and a number of experiments were now performed to optimize the reaction. First, several derivatives of Jacobsen's catalyst were prepared to investigate the influence of the aryl substituent, the axial group on manganese, the scaffold and the Schiff base functionality (Fig. 2). Essentially the same result was obtained with the unsubstituted analogue **2** (entry 3) and the *tert*-butyl group can therefore be omitted. The axial substituent on manganese, on the other hand, needs to be chloride since both the bromide **3** and the acetate **4** gave significantly lower conversion and yield (entries 4 and 5). The same was observed when the *trans*-1,2-diaminocyclohexane scaffold was changed to either the *cis* (*i.e.***5**), the ethylene (*i.e.***6**) or the benzene (*i.e.***7**) analogue (entries 6–8). Notably, the corresponding salan complex **8** also furnished some conversion into the imine (entry 9) which may indicate that the Schiff base functionality in the salen ligand plays an important role in the mechanism (*vide infra*). In the end, *trans-N*,*N′*-bis(salicylidene)-1,2-cyclohexanediamine was selected as the salen ligand for the transformation and thus complex **2** as the catalyst.

**Table 1:** Dehydrogenation with manganese salen/salan complexes^*a*^

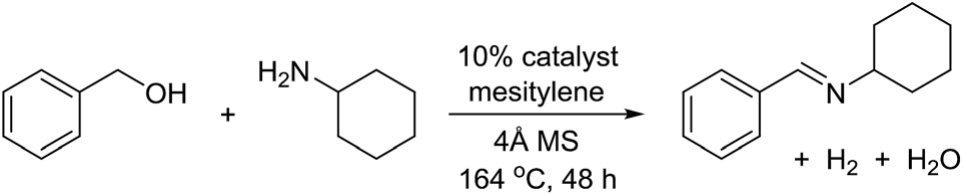			
Entry	Catalyst	BnOH conversion^*b*^ (%)	Imine yield^*c*^ (%)
1	5% Mn_2_(CO)_10_, 10% H_2_salen	—	38
2	**1**	81	79
3	**2**	86	81
4	**3**	70	65
5	**4**	66	64
6	**5**	83	74^*d*^
7	**6**	60	58
8	**7**	27	25
9	**8**	47	12^*e*^

^*a*^Conditions: BnOH (1 mmol), CyNH_2_ (1 mmol), catalyst (0.1 mmol), tetradecane (0.5 mmol, internal standard), 4 Å MS (150 mg), mesitylene (4 mL), reflux, 48 h.

^*b*^Determined by GC using the internal standard.

^*c*^GC yield based on the internal standard.

^*d*^6% of *N*-cyclohexylbenzamide was also formed.

^*e*^Traces of benzyl benzoate was also detected.

For further optimization, the influence of additives and the solvent were investigated (Table 2). A number of common additives^13^ were included in the reaction, but they all led to lower imine yields due to a moderate conversion of benzyl alcohol (results not shown). We have previously used nitride salts as a basic additive for alcohol dehydrogenations,^14^ but lower yields and conversion were also observed when Li_3_N and Mg_3_N_2_ were added to the reaction (entries 1 and 2). However, with Ca_3_N_2_ a complete transformation of benzyl alcohol and a high yield of the imine was now obtained (entry 3). The optimum amount of Ca_3_N_2_ was 16.7% since both higher and lower quantities decreased the yield of the imine (entries 4–7). We observed the same optimum percentage in our earlier work^14^ and 16.7% is an intriguing number since one equiv. of Ca_3_N_2_ can theoretically react as a base with 6 equiv. of either the alcohol or water.

**Table 2:** Optimization of dehydrogenation with complex **2**^*a*^

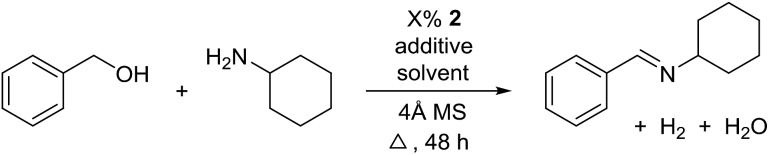					
Entry	X	Additive	Solvent	Conversion^*b*^ (%)	Yield^*c*^ (%)
1	10	20% Li_3_N	Mesitylene	66	12^*d*^
2	10	20% Mg_3_N_2_	Mesitylene	54	48
3	10	20% Ca_3_N_2_	Mesitylene	100	92
4	10	16.7% Ca_3_N_2_	Mesitylene	100	93
5	10	33% Ca_3_N_2_	Mesitylene	100	84
6	10	10% Ca_3_N_2_	Mesitylene	100	90
7	10	5% Ca_3_N_2_	Mesitylene	100	88
8	10	16.7% Ca_3_N_2_	Toluene	100	98
9	10	16.7% Ca_3_N_2_	Dioxane	65	62
10	10	16.7% Ca_3_N_2_	Heptane	20	18
11	5	16.7% Ca_3_N_2_	Toluene	100	96
12	2.5	16.7% Ca_3_N_2_	Toluene	76	73
13	1.25	16.7% Ca_3_N_2_	Toluene	69	66
14	5	20% MgSO_4_	Toluene	80	77
15	5	20% Na_2_SO_4_	Toluene	81	79
16^*e*^	5	16.7% Ca_3_N_2_	Toluene	100	97
17^*e*^	5	20% Ca(OH)_2_	Toluene	99	95

^*a*^Conditions: BnOH (1 mmol), CyNH_2_ (1 mmol), **2** (X/100 mmol), additive, tetradecane (0.5 mmol, internal standard), 4 Å MS (150 mg), solvent (4 mL), reflux, 48 h.

^*b*^Determined by GC using the internal standard.

^*c*^GC yield based on the internal standard.

^*d*^4% of *N*-cyclohexylbenzamide was also formed.

^*e*^Without 4 Å MS.

The influence of the solvent was then investigated and a slightly improved outcome was observed upon conducting the reaction in toluene (entry 8). Dioxane and heptane gave lower yields due to incomplete conversion of benzyl alcohol (entries 9 and 10). Under the reaction conditions, complex **2** is fully soluble at reflux in mesitylene, toluene and dioxane, but not in heptane. The catalyst loading could be adjusted to 5% without affecting the imine yield while 2.5% or 1.25% gave lower conversion of the alcohol (entries 11–13). The role of Ca_3_N_2_ is probably to act as both a base and a desiccant (no byproducts were observed from a potential release of NH_3_). Replacing Ca_2_N_3_ with MgSO_4_ or Na_2_SO_4_ led to a slower reaction (entries 14 and 15) while molecular sieves had no influence on the transformation (entry 16). Ca_3_N_2_ reacts with water to form Ca(OH)_2_ and interestingly this salt gave almost the same result as Ca_3_N_2_ (entry 17). Eventually, 5% of **2** and 16.7% of Ca_3_N_2_ in refluxing toluene were chosen as the optimum conditions for conversion of equimolar amounts of alcohol and amine.

With the optimized catalyst system in place, the substrate scope of the imination reaction could now be investigated. Cyclohexylamine was first reacted with different alcohols and the imines were isolated by flash chromatography (Table 3). This produced 89% yield for the parent reaction with benzyl alcohol while the *p*-methyl analogue furnished 70% yield (entries 1 and 2). The *p*- and *o*-methoxy substituted substrates afforded 83 and 90% yield, respectively, while *p*-phenyl- and *p*-methylthiobenzyl alcohol both gave 60% yield (entries 3–6). *p*-Nitrobenzyl alcohol is often a challenging substrate in dehydrogenative transformations due to an accompanying reduction of the nitro group,^1^ but this was not observed here and the imine was obtained in 70% yield (entry 7). A *p*-fluoro and a *p*-chloro substituent was also tolerated giving 73 and 85% yield (entries 8 and 9). The analogous bromo substrate reacted slightly slower although no competing dehalogenation was observed (entry 10). In addition, the two isomeric naphthalenemethanols gave the corresponding imines in 71 and 60% yield (entries 11 and 12). Full conversion of the alcohol was observed in all cases in Table 3 except for entries 5, 6, 10 and 12 where 5–10% of the alcohol remained and thus accounts for the isolated ∼60% yield.

**Table 3:** Imination of alcohols with cyclohexylamine^*a*^

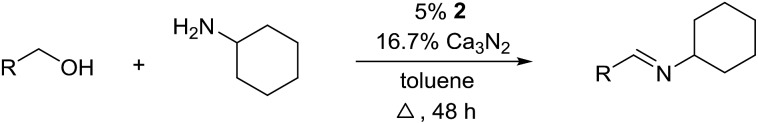			
Entry	Alcohol	Imine	Yield^*b*^ (%)
1	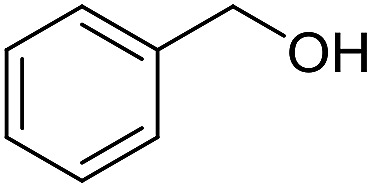	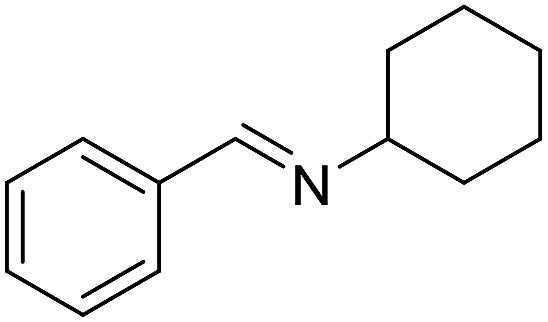	89
2	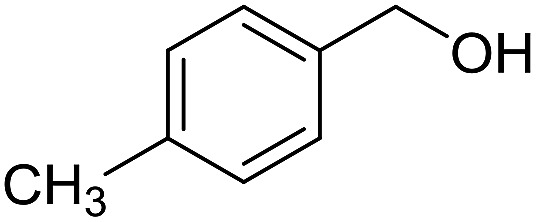	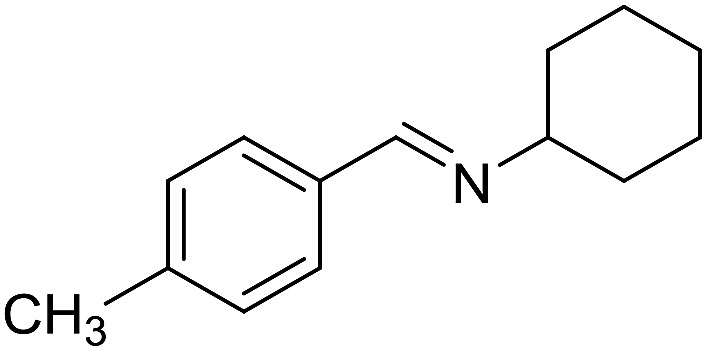	70
3	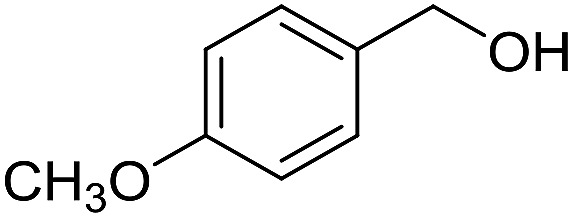	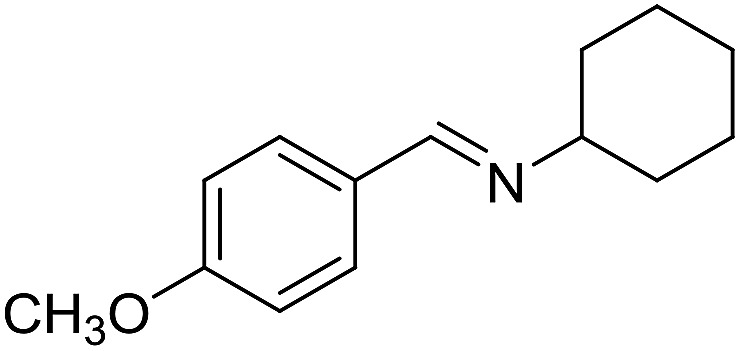	83
4	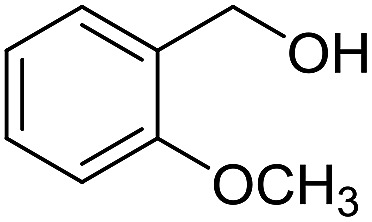	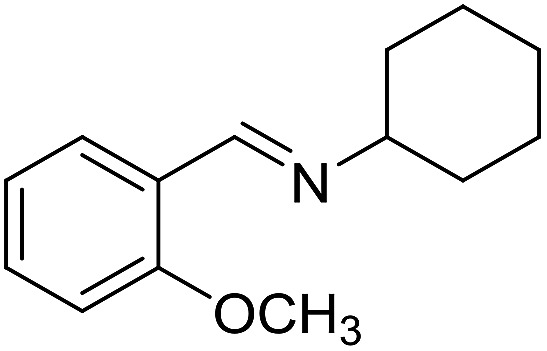	90
5	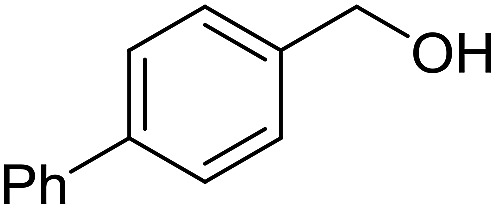	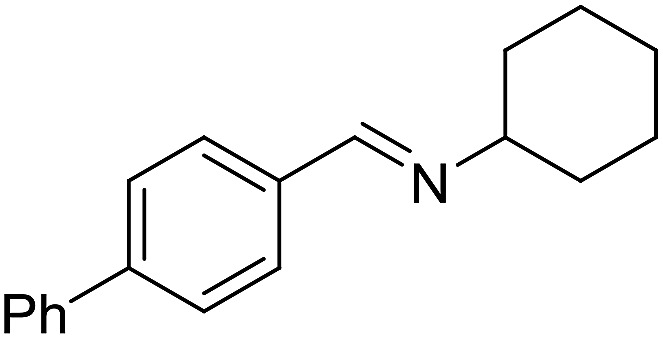	60
6	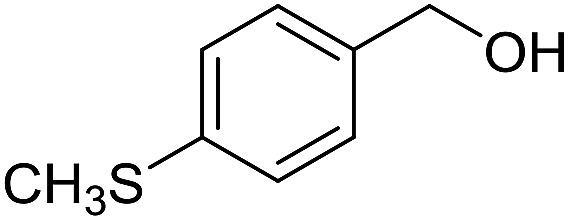	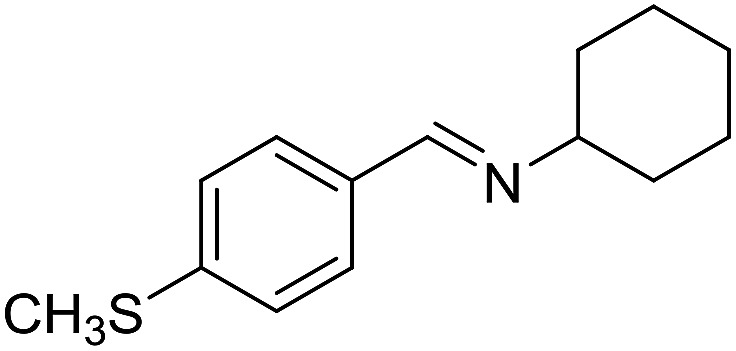	60
7	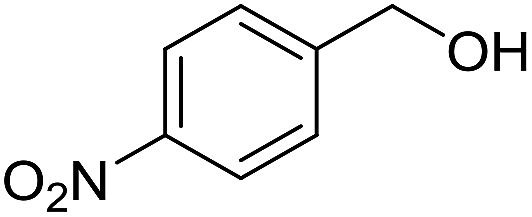	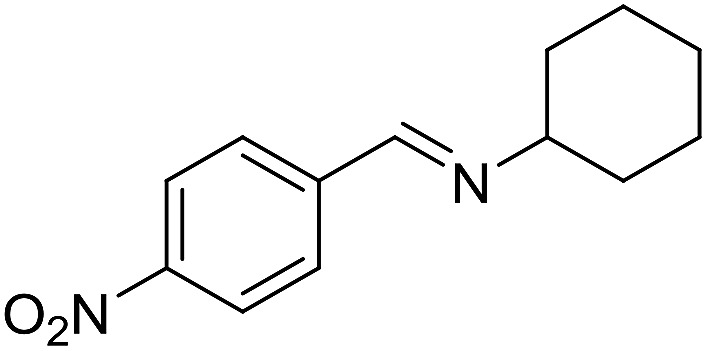	70
8	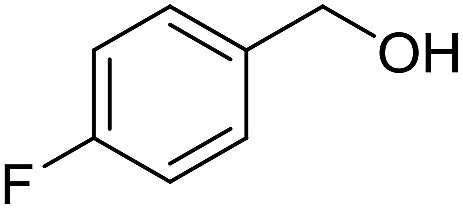	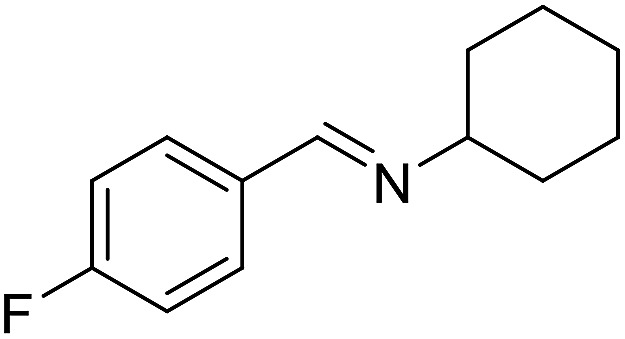	73
9	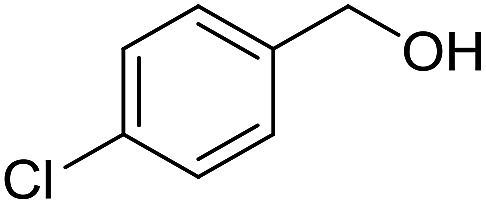	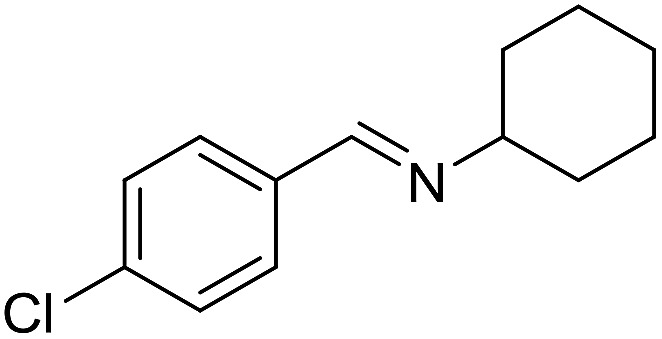	85
10^*c*^	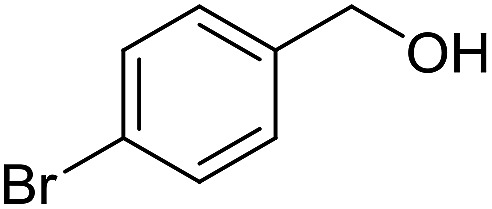	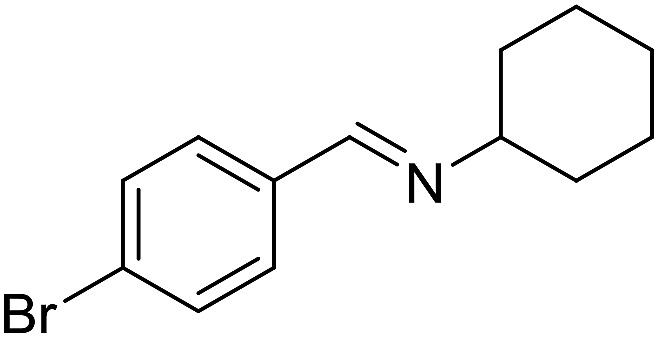	61
11	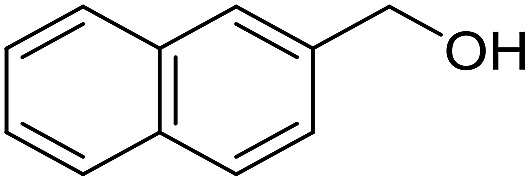	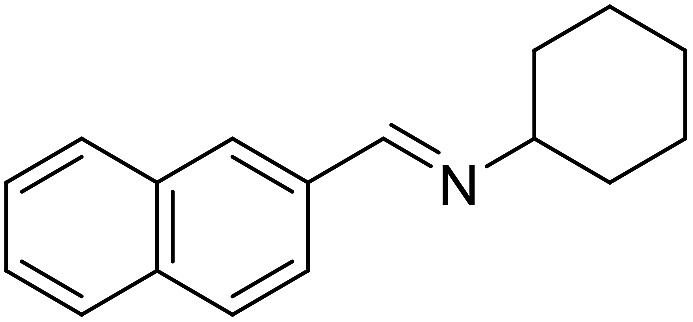	71
12	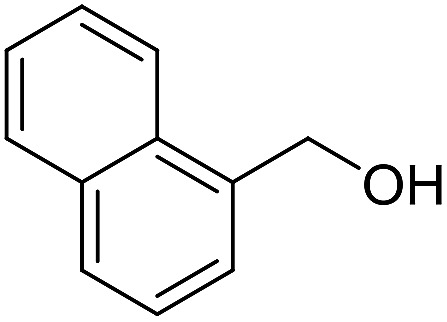	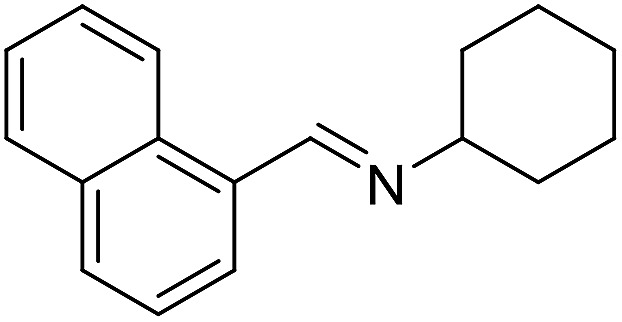	60

^*a*^Conditions: alcohol (1 mmol), CyNH_2_ (1 mmol), **2** (0.05 mmol), Ca_3_N_2_ (0.167 mmol), toluene (4 mL), reflux, 48 h.

^*b*^Isolated yield.

^*c*^Reaction time 72 h.

The influence of the amine was then investigated by reacting benzyl alcohol with different primary amines (Table 4). Octylamine gave essentially the same result as cyclohexylamine (entry 1). The more hindered amines *tert*-octylamine and 1-adamantylamine also gave full conversion of benzyl alcohol and furnished the imine in 69 and 88% yield, respectively (entries 2 and 3). Similar yields were obtained with the two optically pure amines (*R*)-1-phenylethylamine and (*R*)-1-(1-naphthyl)ethylamine although 10–15% of benzyl alcohol remained in both cases (entries 4 and 5). No sign of any racemization was observed in the two transformations indicating that dehydrogenation of the amine is not occurring under the reaction conditions. Benzhydrylamine gave the imine in 73% yield while tritylamine only furnished 19% yield (entries 6 and 7). In the latter case, 48% of the intermediate benzaldehyde was also observed indicating a poor conversion into the imine with the very hindered amine. The inferior reactivity is most likely caused by Ca_3_N_2_ since a control experiment with benzaldehyde, tritylamine and Ca_3_N_2_ also showed incomplete imine formation.^15^ Furthermore, the imination could be performed with anilines where 74%, 63% and 62% yield were obtained with aniline, *p*-anisidine and *p*-trifluoromethylaniline, respectively (entries 8–10). In entries 9 and 10 as well as in entries 5 and 6 about 10–30% of unreacted benzyl alcohol was also detected.

**Table 4:** Imination of amines with benzyl alcohol^*a*^

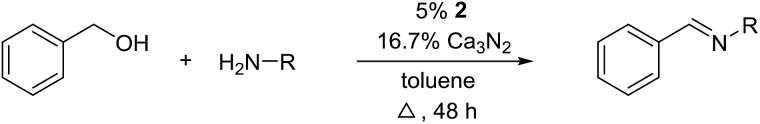			
Entry	Amine	Imine	Yield^*b*^ (%)
1	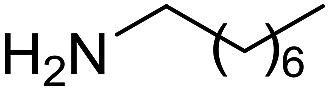	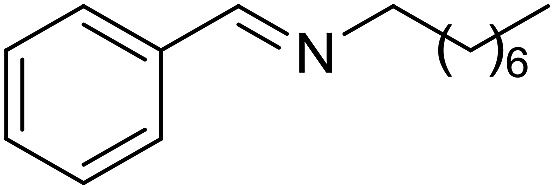	90
2	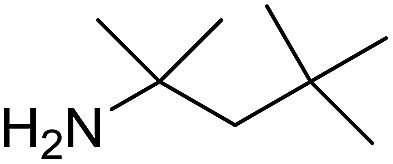	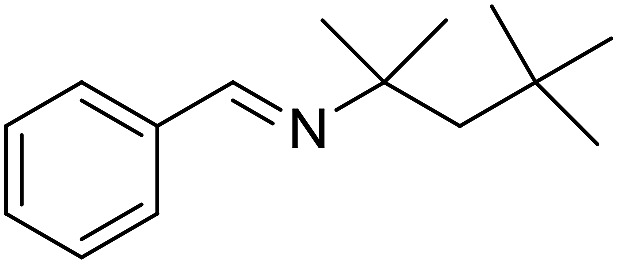	69
3	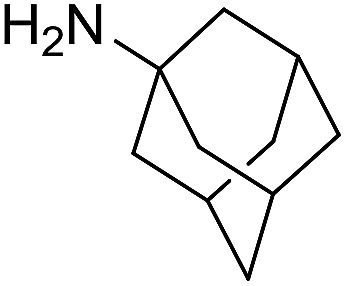	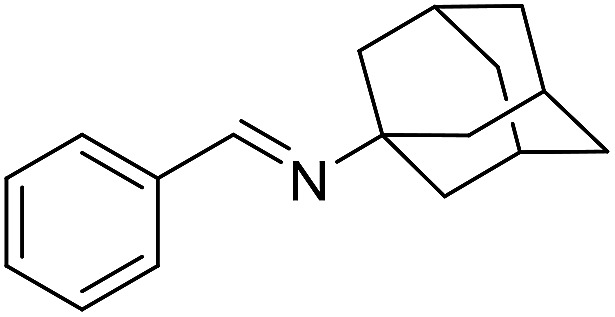	88
4	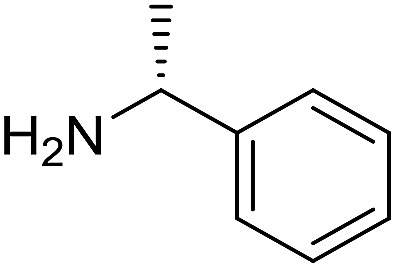	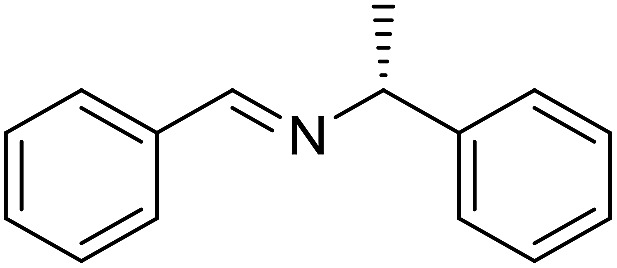	66
5	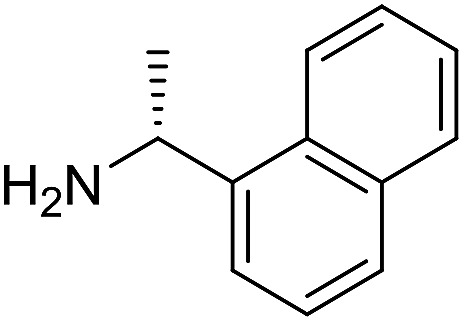	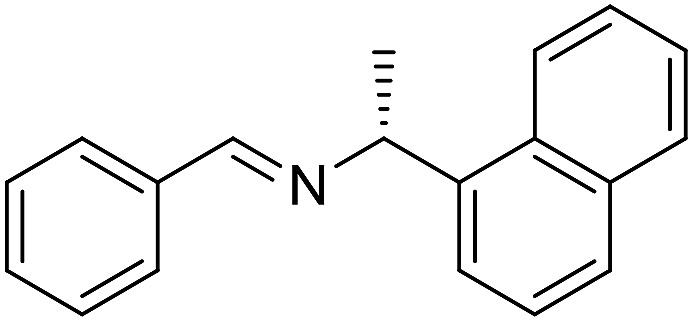	83
6	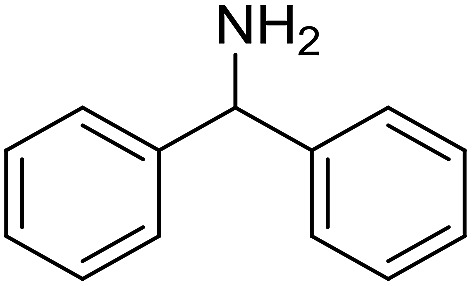	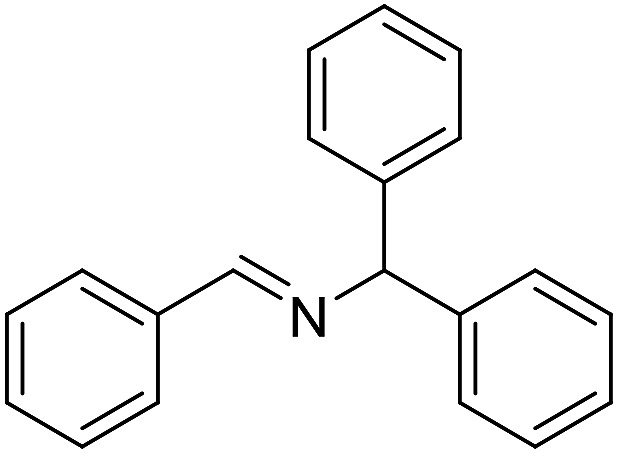	73
7	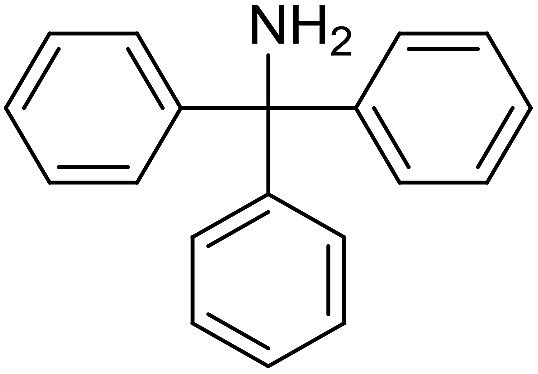	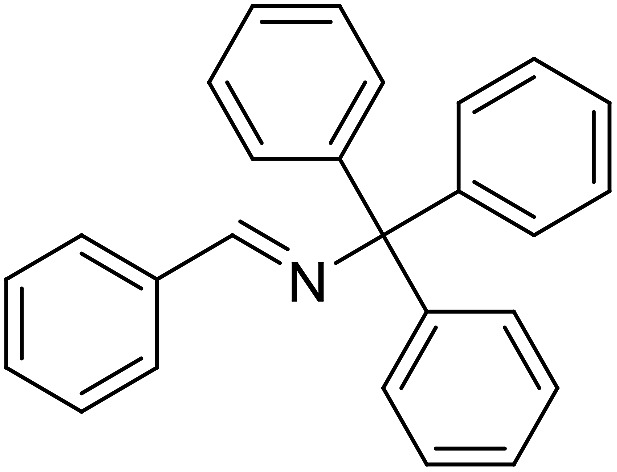	19
8	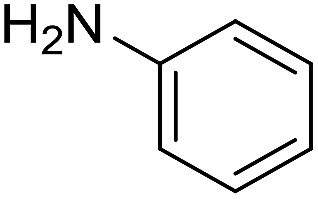	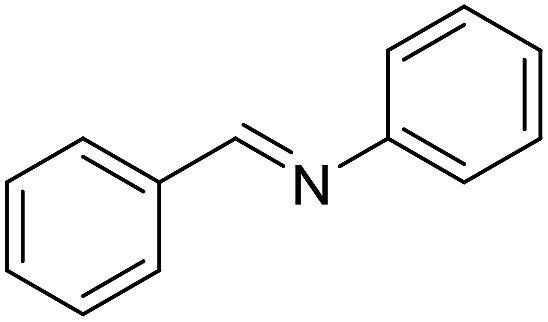	74
9	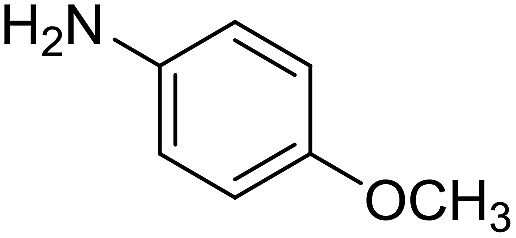	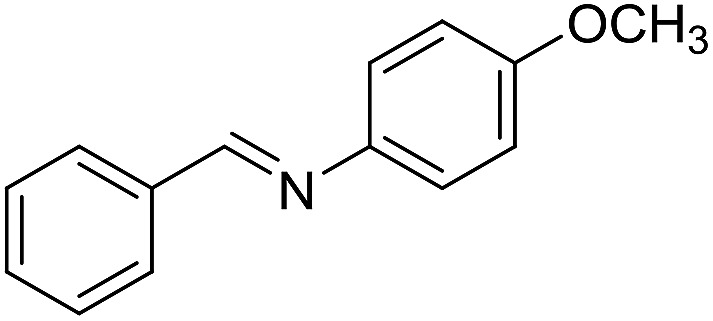	63
10	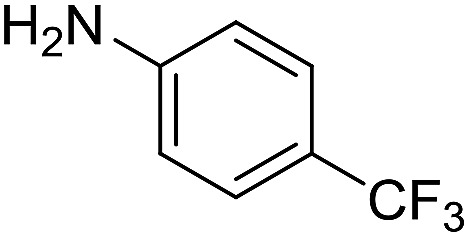	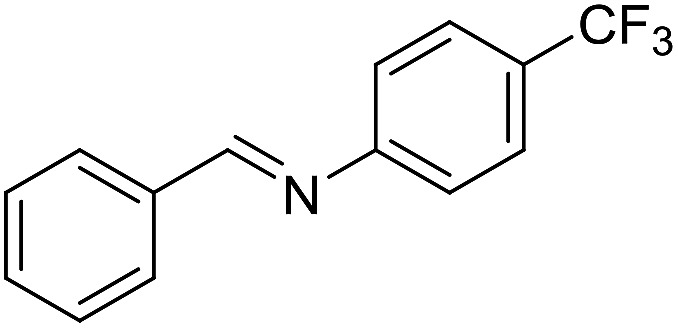	62

^*a*^Conditions: BnOH (1 mmol), amine (1 mmol), **2** (0.05 mmol), Ca_3_N_2_ (0.167 mmol), toluene (4 mL), reflux, 48 h.

^*b*^Isolated yield.

**Scheme 1:**
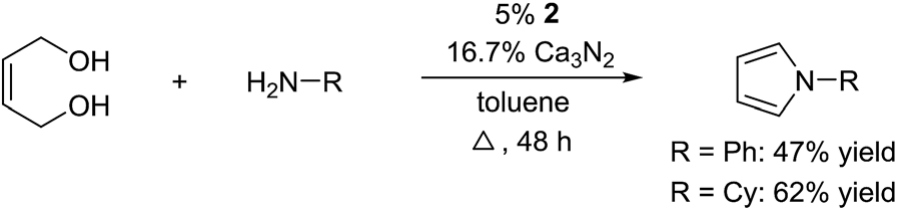
Pyrrole synthesis from but-2-ene-1,4-diol and amines.

The reaction could be extended to the synthesis of pyrroles by reacting *cis*-but-2-ene-1,4-diol with primary amines.^16^ The transformation could be carried out with both aniline and cyclohexylamine to afford the desired product in moderate yield^17^ (Scheme 1).

The gas evolution was measured in a separate experiment under the optimized conditions in Table 2, entry 16, but without adding Ca_3_N_2_ (resulting in a slightly lower 78% imine yield). One equivalent was collected and the gas was identified as dihydrogen, which confirms the acceptorless dehydrogenative pathway. Essentially no imine was formed when the reaction was conducted in the absence of complex **2**. The possibility for trace metal impurities is a serious concern in the development of reactions with new metal catalysts^18^ and studies have shown that some coupling reactions with manganese catalysts are most likely mediated by traces of other elements.^19^ For that reason, complex **2** was analyzed by inductively coupled plasma mass spectrometry (ICP-MS) for traces of other metals known to perform alcohol dehydrogenations. However, none of these elements could be detected beyond their detection limit and it is therefore highly unlikely that another metal is responsible for the observed results.

As mentioned above, complex **2** dissolves completely in toluene upon heating the mixture to reflux. However, after the imination has gone to completion and the reaction is cooled to room temperature, complex **2** precipitates out again and 95% can be recovered after addition of a small amount of hexane. Furthermore, the retrieved complex can be subjected to a new catalytic reaction which gave 82% yield of the imine with a 4.5% catalyst loading.

When the optimized reaction in Table 2, entry 16 was performed with PhCD_2_OH instead of PhCH_2_OH, the product was exclusively PhCD=NCy with no hydrogen incorporation into the benzylic position. This may imply that the dehydrogenation takes place by a monohydride pathway and no metal-dihydride species is formed.

To investigate whether the hydride abstraction takes place in the rate-limiting step, the primary kinetic isotope effect (KIE) was measured. The initial rate was determined with both PhCD_2_OH and PhCH_2_OH in the reaction with cyclohexylamine which gave a KIE of 2.00. This somewhat modest value shows that breakage of the C–H bond is one of several slow steps in the transformation.

**Fig. 3:**
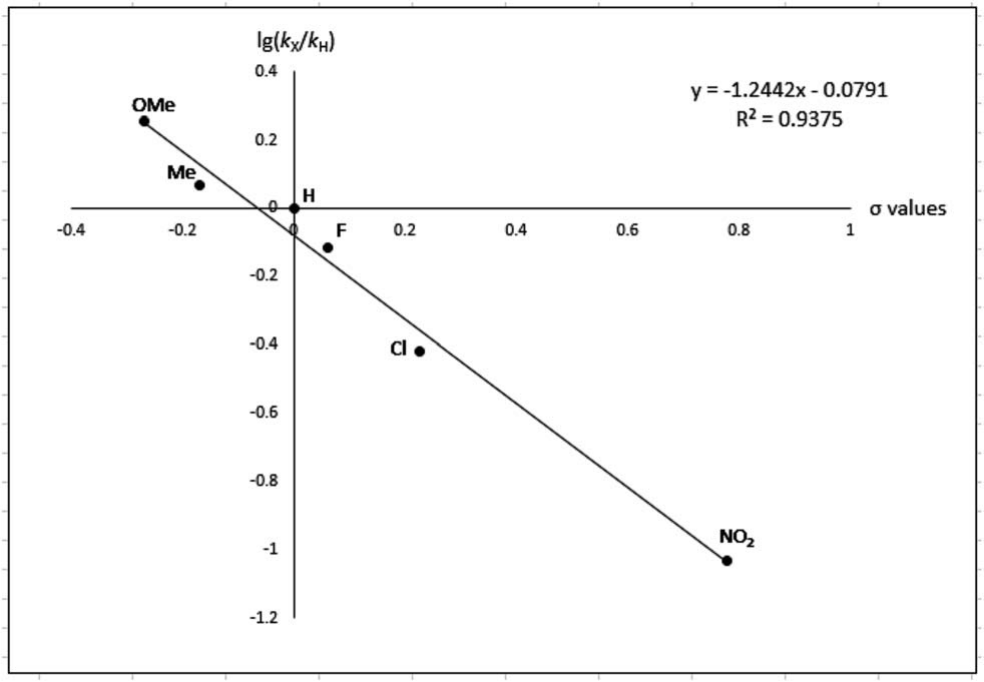
Hammett plot for the imination with *para*-substituted benzyl alcohols.

To gain more experimental information about the reaction pathway, the studies were also supplemented by a Hammett study where the change in charge at the benzylic position between the starting material and the transition state can be determined. We have previously used Hammett studies with *para*-substituted benzyl alcohols to analyze the rate-limiting step in dehydrogenations catalyzed by ruthenium and iridium complexes.^20^ Thus, five *para*-substituted benzyl alcohols (X = OCH_3_, CH_3_, F, Cl and NO_2_) were allowed to compete with the parent benzyl alcohol in the imination with cyclohexylamine. The reactions were monitored by GC, which allowed for determining the consumption of each alcohol. Assuming a first order reaction in the alcohol, their relative reactivities (*k*_X_/*k*_H_) can be determined as the slope of the line when ln(*c*_0_/*c*) for one *para*-substituted benzyl alcohol is plotted against the same values for benzyl alcohol. These plots gave straight lines for all five *para*-substituted benzyl alcohols and made it possible to use the Hammett equation lg(*k*_X_/*k*_H_) = *σρ* to construct a Hammett plot (Fig. 3). A good correlation was obtained with the standard *σ* values^21^ giving a straight line with a *ρ* value of –1.24. This negative slope shows that substrates with electron-donating groups react faster and that a partial positive charge is built up at the benzylic carbon in the rate-limiting step consistent with a hydride transfer from the alcohol. Attempts to use different sets of *σ* values^22^ gave a poor correlation and radical intermediates are therefore not involved in the catalytic cycle. This was also confirmed by conducting the imination in the presence of one equiv. of the radical trapping agents cyclohexa-1,4-diene and 2,4-diphenyl-4-methylpent-1-ene which in both cases had no influence on the imine yield.

**Fig. 4:**
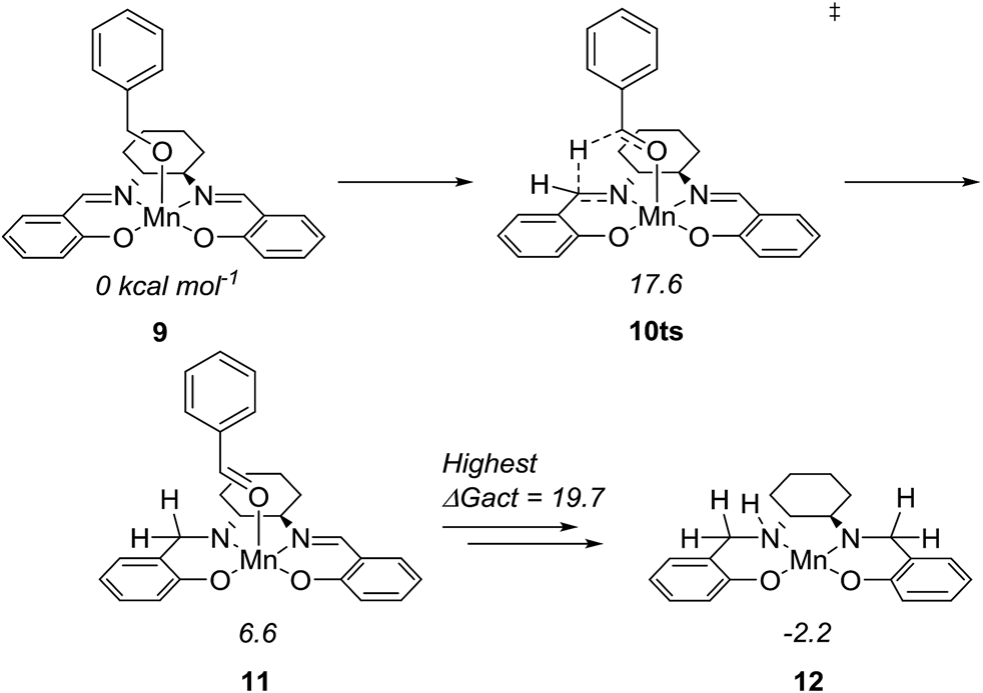
Activation of Mn(iii)(salen)OBn to form the active amido complex.

To further understand the mechanism, we used density functional theory (DFT) calculations to estimate the Gibbs free energies of possible intermediates and transition states. The starting point was the Mn(salen)OBn complex **9**, where Cl has been replaced by a benzylic alkoxide (Fig. 4). This transformation involves the elimination of HCl, which is possible under the basic conditions. In fact, an experiment in the absence of a base (complex **2** and benzyl alcohol) gave no conversion at all while the absence of the amine (complex **2**, benzyl alcohol and Ca_3_N_2_) resulted in 20% of benzyl benzoate.

The initial idea was that complex **9** undergoes β-hydride elimination. However, the activation energy for this process was found to be prohibitively high (at 37.9 kcal mol^–1^ relative to **9**) which is partly due to the lack of an available coordination site. Instead, we found an alternative reaction where the hydride is transferred from the benzylic carbon to the imine carbon of the salen ligand. The activation energy was merely 17.6 kcal mol^–1^ and the product complex **11** is at 6.6 kcal mol^–1^. A similar pathway has been identified in the activation of (PNNP)Fe(ii) eneamido complexes with isopropanol.^23^ The product benzaldehyde is then replaced by benzyl alcohol, from which a proton is transferred to the amide nitrogen of the reduced salen ligand. The resulting complex where one imine of the salen is hydrogenated is at –5.2 kcal mol^–1^ relative to **9**. The lowest activation energy found from the hydrogenated intermediate was for another hydride transfer to the second imine of the salen, with a transition state at 14.5 kcal mol^–1^ (maximum Δ*G*_act_ = 19.7 kcal mol^–1^, see ESI† for more details).

**Fig. 5:**
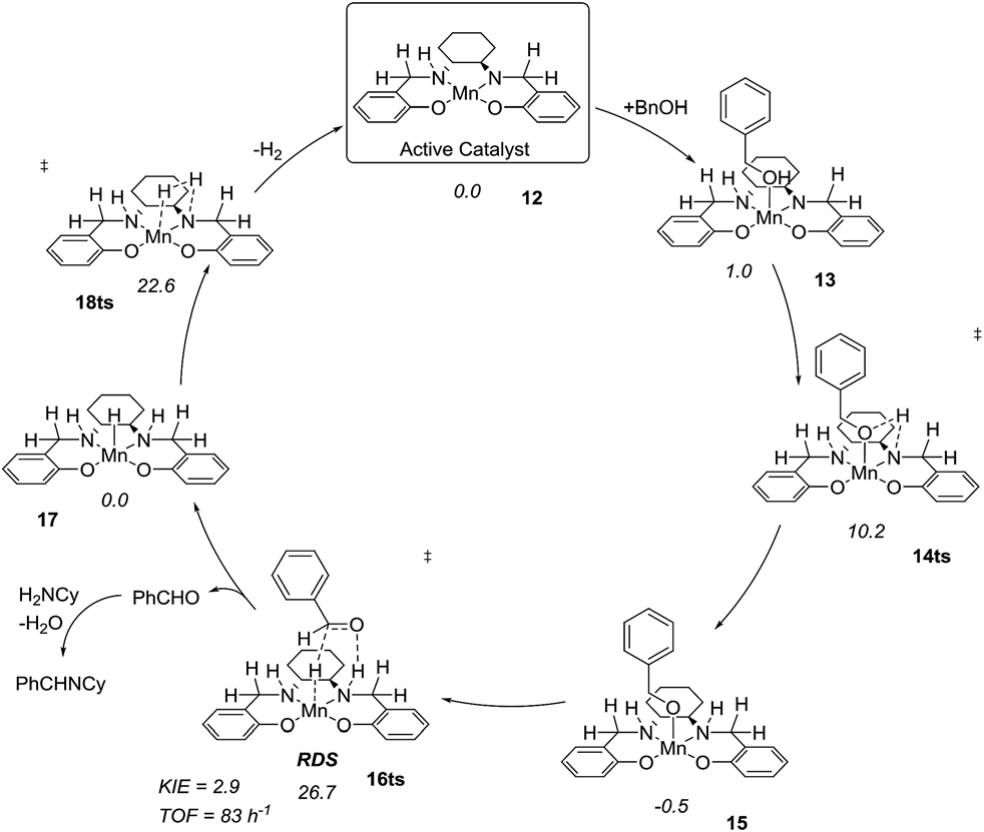
Proposed catalytic cycle and relative Gibbs free energies.

**Fig. 6:**
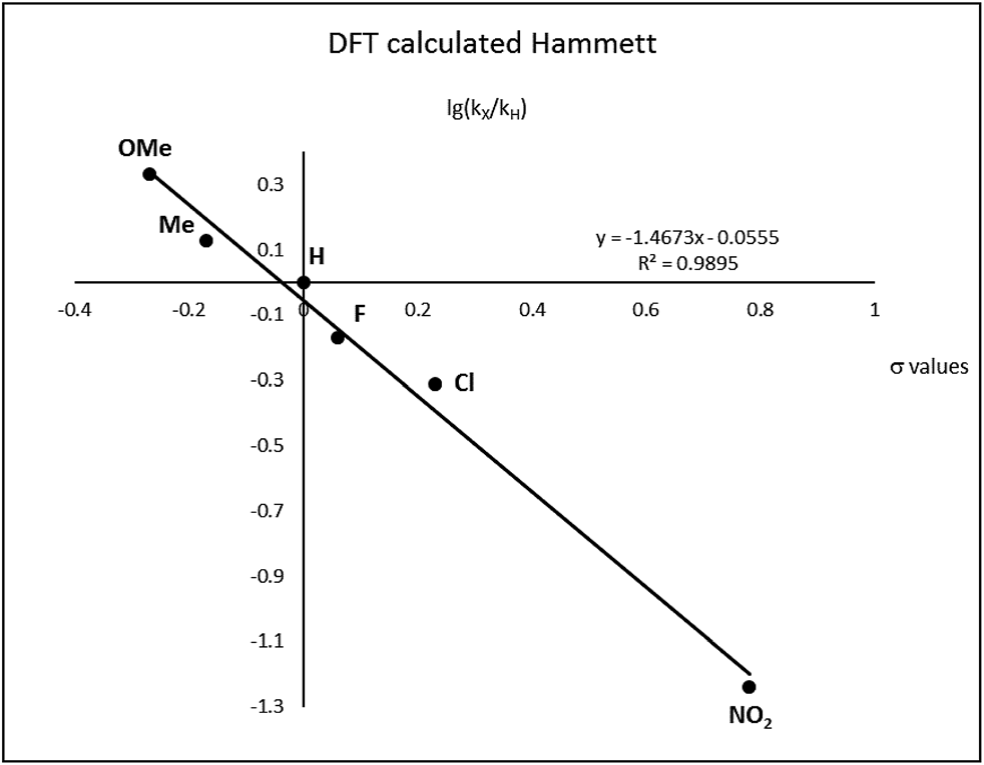
Hammett plot calculated by DFT.

After complete dissociation of benzaldehyde a key species **12** is formed, where one imine is hydrogenated to the amine and the other is reduced to an amide ligand. This species resembles intermediates from alcohol dehydrogenations with (PNP)Ru(ii), (PNP)Mn(i), (PNNP)Fe(ii) and (PNP)Ir(iii) catalysts^24^ which have an amide ligand that can act as a Brønsted base and a metal that can serve as a hydride acceptor. They have all been proposed to react *via* an outer-sphere hydrogen transfer mechanism.^24^ The main difference is the metal and the oxidation state, which in the current case is manganese(iii). The same outer-sphere hydrogen transfer was identified here and the activation energy is 26.7 kcal mol^–1^ relative to **12** and 27.2 kcal mol^–1^ relative to **15**, which corresponds to a TOF of 83 h^–1^ at the reaction conditions (Fig. 5). The calculated KIE of this reaction is 2.9, which is in reasonable agreement with the experimental value of 2.0. Finally, we calculated the relative rates of the *para*-substituted benzyl alcohols used in the experimental study. The relative rates were calculated from the prereactive complex **15** and a good agreement was found with the experimental results giving a very similar *ρ* value (Fig. 6). After the formation of the manganese(iii) hydride intermediate **17**, benzaldehyde is assumed to react irreversibly with the amine. From complex **17** the formation of hydrogen gas requires an activation energy of 22.6 kcal mol^–1^, in a step that regenerates the active catalyst.

The proposed mechanism indicates that the Schiff base functionality of the salen ligand is crucial for the reactivity. This was also confirmed by isolating the salen complex after the imination reaction with PhCD_2_OH and cyclohexylamine. The isolated complex showed the incorporation of one deuterium atom on each of the Schiff base carbons as would be expected from the proposed mechanism. The involvement of the Schiff base was also observed in the optimization where both the salen complex **2** and the salan complex **8** catalyzed the dehydrogenation although the latter in a low yield (Table 1). When the imination with 5% of the salan complex **8** was repeated with KO*t*Bu as the base, a 56% yield was obtained of *N*-benzylidene cyclohexylamine. This result can be explained by elimination of HCl from **8** to afford the catalytically active species **12**.^25^ Similar eliminations of hydrogen halides under basic conditions have been described with (PNP)Mn(i), (PNP)Ru(ii) and (PNNP)Fe(ii) complexes to form the corresponding amido compounds.4b,^26^ Notably, a post analysis by LCMS of the imination with salan complex **8** showed the formation of salen complex **2** as the main manganese species together with minor amounts of some unidentified complexes. None of the starting complex **8** could be detected after the imination. These observations show that a salan complex can be converted into the corresponding salen complex under the reaction conditions and explains why salen complex **2** is almost fully recovered after the reaction. So far, however, we have not been able to identify a pathway by which hydrogen is eliminated from a salan complex to afford the salen compound.

## Conclusions

In summary, we have described a new catalyst for the acceptorless dehydrogenation of alcohols. The manganese(iii) salen complex **2** mediates the formation of imines from alcohols and amines with the liberation of hydrogen gas. The reaction can be performed with different alcohols and amines and can be extended to the synthesis of pyrroles. Complex **2** can be recovered from the reaction and used again without significantly affecting the catalytic activity. The mechanism is believed to involve a bifunctional pathway where both the metal and the ligand participates in the dehydrogenation reaction. We envision the discoveries will spur much interest in the development of new transformations with hydrogen gas and manganese(iii) catalysts.

## Conflicts of interest

There are no conflicts to declare.

## Supplementary Material

Supplementary information
