# Supplementary material for: Development and mechanistic investigation of the manganese(iii) salen-catalyzed dehydrogenation of alcohols
Source: Chem Sci. 2018 Nov 13;10(4):1150–7. doi: 10.1039/c8sc03969k (PMC6349018; doi:10.1039/c8sc03969k)
Supplement: Supplementary file 1 [file SC-010-C8SC03969K-s001.pdf]

Electronic Supplementary Material (ESI) for Chemical Science.
This journal is © The Royal Society of Chemistry 2018

SUPPLEMENTARY INFORMATION

Development and mechanistic investigation of the manganese(III) salen-catalyzed dehydrogenation of alcohols

Simone V. Samuelsen,[a] Carola Santilli,[a] Mårten S. G. Ahlquist,[b] and Robert Madsen*[a]

[a]*Department of Chemistry, Technical University of Denmark, 2800 Kgs. Lyngby, Denmark*

[b]*Department of Theoretical Chemistry & Biology, School of Engineering Sciences in Chemistry Biotechnology and Health, KTH Royal Institute of Technology, 10691 Stockholm, Sweden*

Table of Contents

	Page
General experimental methods	2
General procedure for imine synthesis	2
Gas development	2
Deuterium labelling study	3
Determination of deuterium isotope effect	3
Hammett study	4
Procedures for ligand synthesis	7
Procedures for manganese(III) catalyst synthesis	9
Characterization data for imines	13
Computational details	22
Activation of catalyst and catalytic cycle	23
Energies of complexes	24
Cartesian coordinates of complexes	24
References	43
^{1}H and ^{13}C NMR spectra	44

General experimental methods

NMR spectra were recorded at 400 MHz for ^{1}H-NMR and 101 MHz for ^{13}C-NMR on a Bruker Ascend 400 MHz spectrometer. Chemical shift values (δ) are reported in ppm relative to the residual solvent signal in $CDCl_3$ (δ_H 7.26 ppm, δ_C 77.2 ppm) while coupling constants (*J*) are given in Hz. High Resolution mass spectra were recorded using ESI with TOF detection. GCMS was carried out on a Shimadzu GCMS-QP2010S instrument fitted with an Equity 5, 30m×0.25mm×0.25µm column. Ionisation was performed by electronic impact (EI, 70eV) and helium as the carrier gas. Flash column chromatography was performed using silica gel 60 (0.035-0.070 mm particle size) saturated with Et_3N. Mesitylene was dried over molecular sieves (4 Å) while toluene was obtained by using a Pure Solv™ Micro solvent purification system. The water content of the solvents and liquid reagents was measured on a Karl-Fischer apparatus. All experiments were carried out under a nitrogen flow using Schlenk flask techniques except from the synthesis of catalysts.

General procedure for imine synthesis

Manganese complex **2** (20.6 mg, 0.05 mmol) and Ca_3N_2 (24.8 mg, 0.167 mmol) were placed in an oven-dried tube, where after it was placed in a carousel. Vacuum was applied and the flask was then filled with nitrogen gas (repeated three times). Anhydrous toluene (4 mL) was added and the reaction mixture was heated to reflux. Alcohol (1 mmol), amine (1 mmol) and tetradecane (0.5 mmol as internal standard) were added by a syringe, and the reaction was refluxed with stirring under a flow of nitrogen for 48 h. The mixture was cooled to room temperature and the solvent removed in vacuo. The crude product was purified by silica gel column chromatography (hexane with 2% Et_3N) to afford the desired imine or pyrrole.

Gas development

Manganese complex **2** (20.6 mg, 0.05 mmol) was placed in an oven-dried Schlenk tube. The tube was subjected to vacuum and then filled with nitrogen gas (repeated three times). Freshly degassed, anhydrous toluene (4 mL) was added and the reaction mixture was heated to reflux. Benzyl alcohol (108 mg, 1 mmol), cyclohexylamine (99.0 mg, 1 mmol) and tetradecane (99.0 mg, 0.5 mmol, internal standard) were then added and the reaction tube was connected to a burette filled with water. The bottom of the burette was further connected to a water reservoir. The reaction mixture was refluxed for 48 h after which 18 mL

(0.73 mmol) of gas was collected in the burette. A GC sample of the reaction mixture showed 78% yield of the imine and full conversion of the alcohol and the amine. The identity of the gas was established from a ^{1}H NMR spectrum in toluene-d_8.

Deuterium labelling study

Benzyl alcohol-α,α-d_2 (110 mg, 1.0 mmol) and cyclohexylamine (99.0 mg, 1.0 mmol) were placed in an oven-dried tube and subjected to the imination reaction following the general procedure for imine synthesis. After purification of the product imine, examination of the ^{1}H-NMR revealed that the product imine was obtained as a pure deuterium-labeled imine and no hydrogen/deuterium scrambling had occurred.

The transformation was repeated and the salen catalyst isolated after the reaction. LCMS showed the same retention time as complex **2** and the molecular mass had increased by 2. The re-isolated catalyst was hydrolyzed with an excess amount of aqueous hydrochloric acid to yield the parent aldehyde of the ligand. Examination by ^{1}H-NMR revealed that the obtained aldehyde was salicylaldehyde-α-d_1 and the re-isolated catalyst is therefore the following:

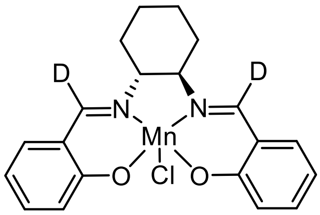

Determination of deuterium isotope effect

Benzyl alcohol (108 mg, 1.0 mmol) and cyclohexylamine (99 mg, 1.0 mmol) were placed in an oven-dried tube and subjected to the imination reaction following the general procedure for imine synthesis. For 5 h, a sample of 50 µL was taken out every 30 minutes, transferred to a GC vial, diluted to 1 mL with diethyl ether and then subjected to GCMS analysis to follow the formation of *N*-benzylidenecyclohexylamine and determine the initial rate (r). The same procedure was repeated using benzyl alcohol-α,α-d_2 (110 mg, 1.0 mmol) instead of non-deuterated benzyl alcohol. The initial rate for the transformation of benzyl alcohol was r_H = 1.00 10^{-5}. The initial rate for the reaction of benzyl alcohol-α,α-d_2 was r_D = 5.00 10^{-6}. The isotope effect was k_H/k_D = 2.00.

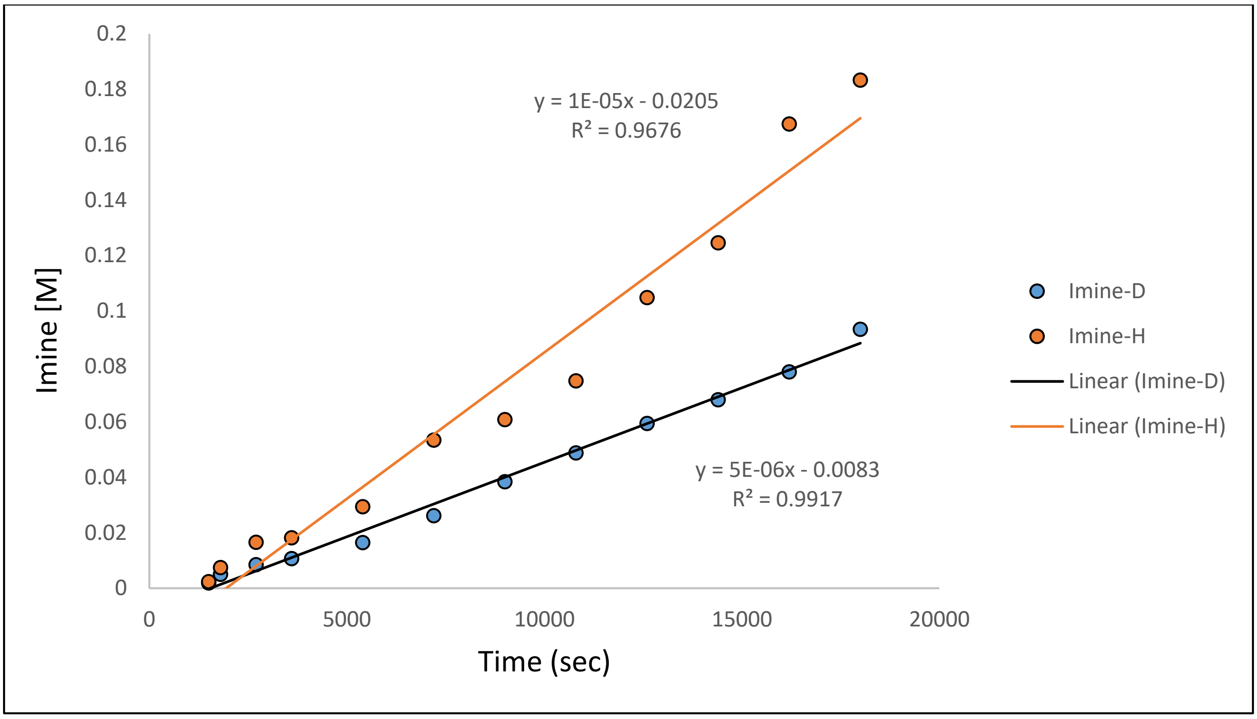

Hammett study

Benzyl alcohol (54 mg, 0.5 mmol), 4-substituted benzyl alcohol (0.5 mmol) and cyclohexylamine (99 mg, 1.0 mmol) were placed in an oven-dried tube and subjected to the imination reaction following the general procedure for imine synthesis. For 5 h, a sample of 50 µL was taken out every 30 minutes, transferred to a GC vial, diluted to 1 mL with diethyl ether and then subjected to GCMS analysis to follow the formation of *N*-benzylidenecyclohexylamine and the 4-substituted *N*-benzylidenecyclohexylamine to determine k_{rel}.

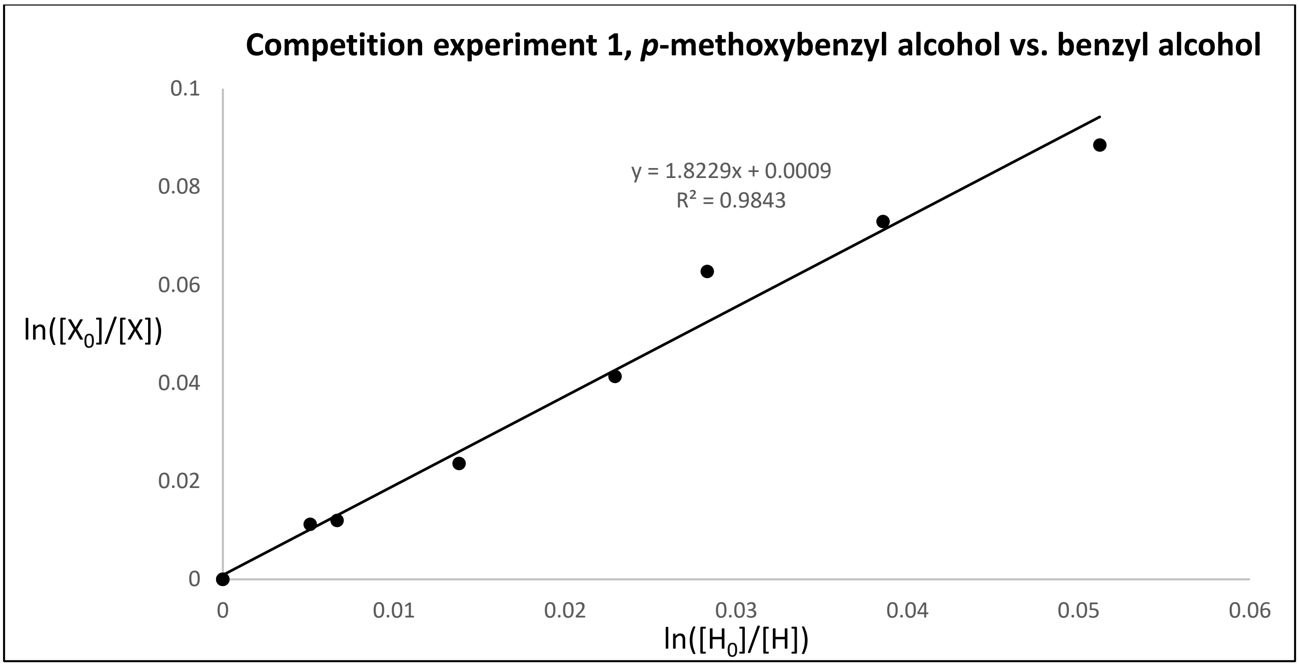
Competition experiment 1, p-methoxybenzyl alcohol vs. benzyl alcohol
y = 1.8229x + 0.0009
R² = 0.9843
ln([X0]/[X])
ln([H0]/[H])
0
0.02
0.04
0.06
0.08
0.1
0.01
0.03
0.05
0.06

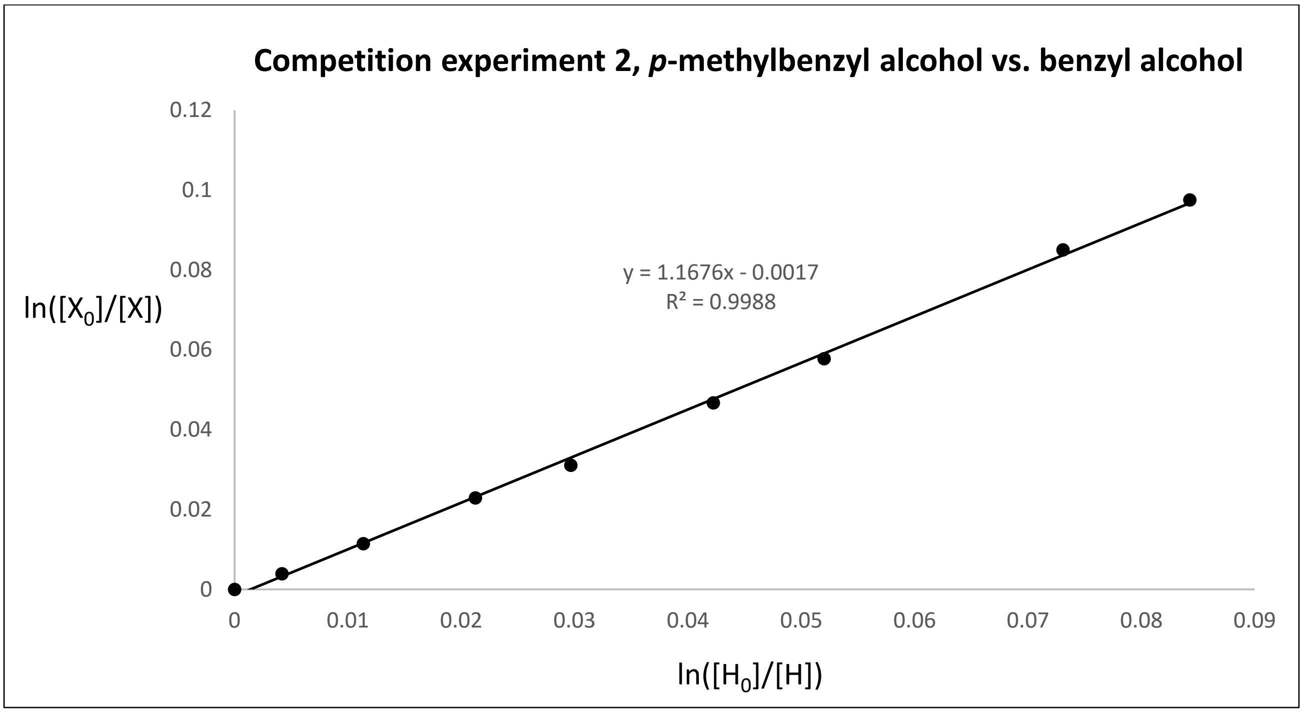
Competition experiment 2, p-methylbenzyl alcohol vs. benzyl alcohol
y = 1.1676x - 0.0017
R² = 0.9988
ln([X0]/[X])
ln([H0]/[H])
0
0.02
0.04
0.06
0.08
0.1
0.12
0.01
0.03
0.05
0.07
0.09

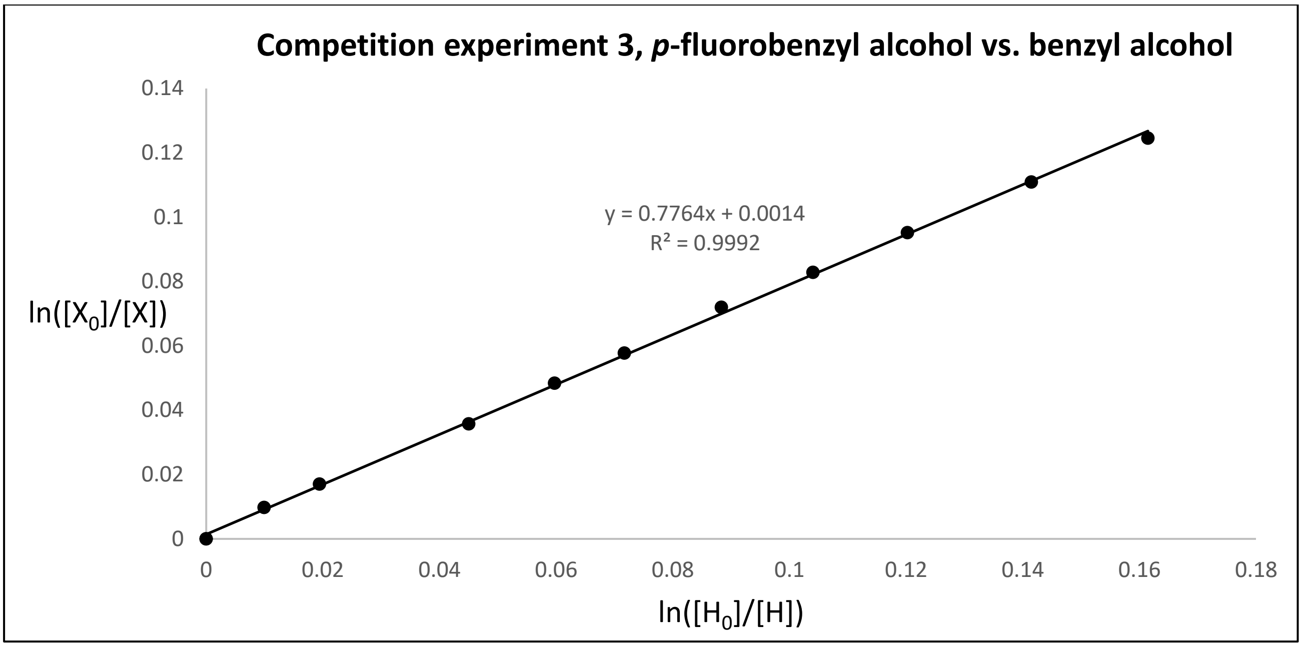

Competition experiment 3, p-fluorobenzyl alcohol vs. benzyl alcohol
y = 0.7764x + 0.0014
R² = 0.9992
ln([X0]/[X])
ln([H0]/[H])
0
0.02
0.04
0.06
0.08
0.1
0.12
0.14
0.16
0.18

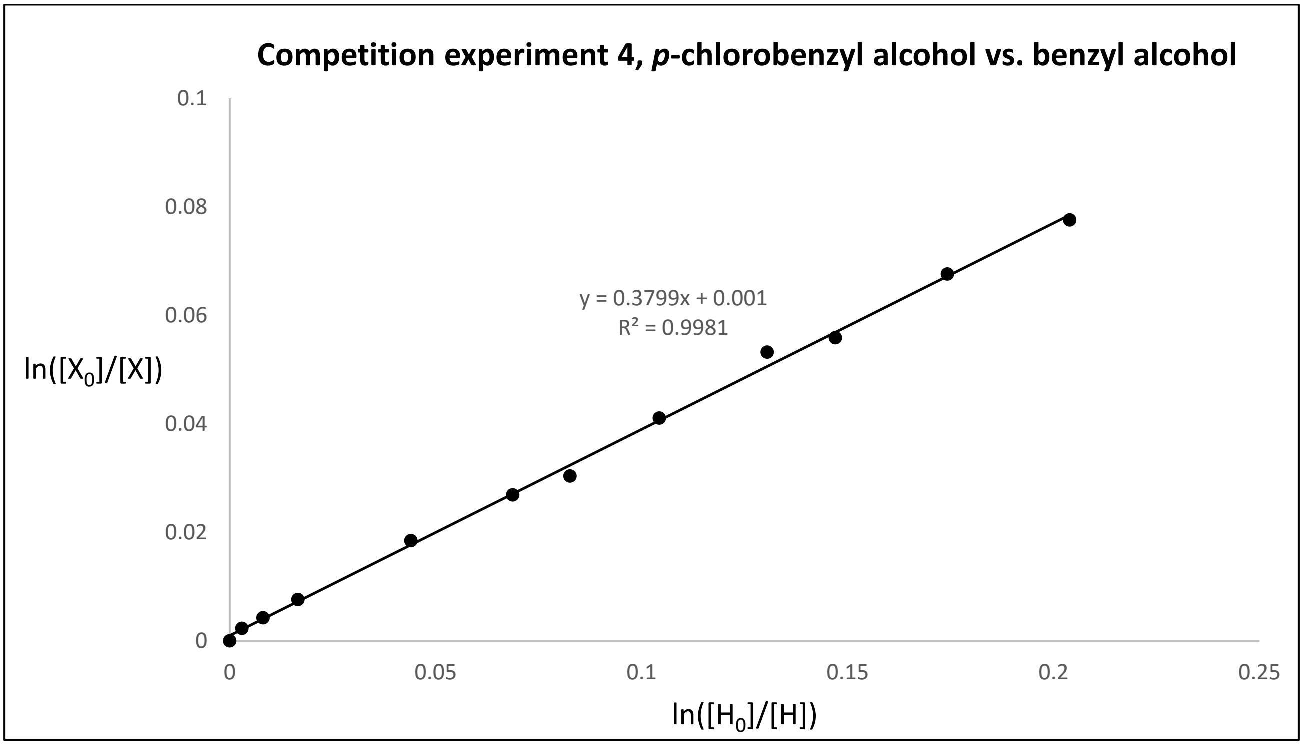

Competition experiment 4, p-chlorobenzyl alcohol vs. benzyl alcohol
y = 0.3799x + 0.001
R² = 0.9981
ln([X0]/[X])
ln([H0]/[H])
0
0.02
0.04
0.06
0.08
0.1
0.05
0.15
0.2
0.25

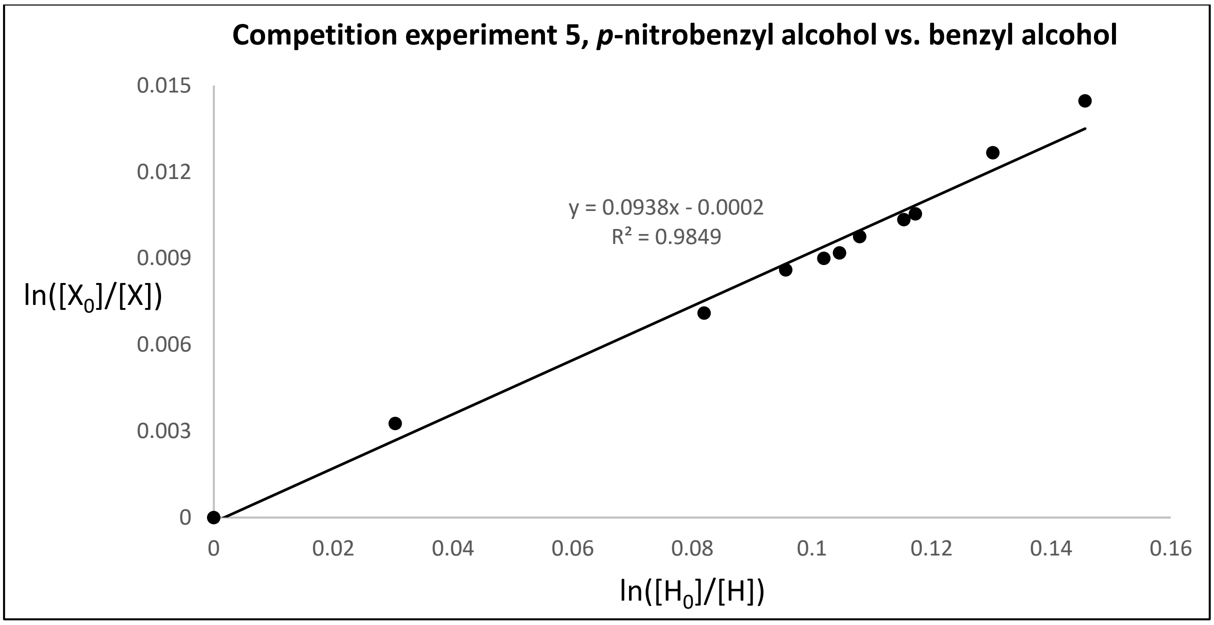

Procedures for ligand synthesis

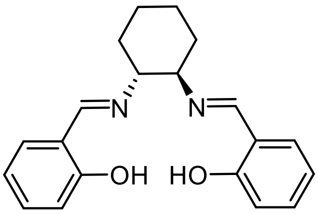

(1*R*,2*R*)-*N*,*N'*-Bis(salicylidene)-1,2-cyclohexanediamine

A mixture of (1*R*, 2*R*)-(+)-1,2-diaminocyclohexane L-tartrate (1.0 g, 3.8 mmol), K_2CO_3 (525 mg, 3.8 mmol) and water (2.5 mL) was stirred until complete dissolution, then methanol (20 mL) was added. The reaction mixture was heated to reflux and a solution of salicylaldehyde (0.80 mL, 7.6 mmol) in methanol (8 mL) was added over 30 min. The reaction mixture was refluxed for an additional 4 h and was cooled to room temperature. The mixture was concentrated in vacuo and the residue was dissolved in ethyl acetate (15 mL), washed with water (8 mL), dried (Na_2SO_4) and concentrated in vacuo to give the desired ligand as a yellow oil. Yield: 1.21 g (99%). ^{1}H-NMR (400 MHz, $CDCl_3$) δ ppm: 8.26 (s, 2H), 7.26-7.21 (m, 2H), 7.15 (dd, *J* = 7.7, 1.7 Hz, 2H), 6.88 (dd, *J* = 8.3, 1.0 Hz, 2H), 6.79 (td, *J* = 7.6, 1.1 Hz, 2H), 3.37-3.25 (m, 2H), 1.98-1.84 (m, 4H), 1.78-1.67 (m, 2H), 1.54-1.41 (m, 2H). ^{13}C-NMR (101 MHz, $CDCl_3$) δ ppm: 164.1, 161.1, 132.3, 131.6, 118.8, 118.7, 116.9, 72.8, 33.2, 24.3. NMR data are in accordance with literature values.[1]

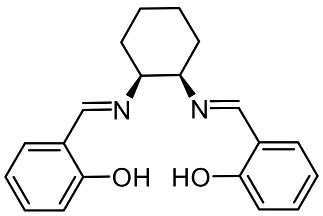

(1*S*,2*R*)-*N*,*N'*-Bis(salicylidene)-1,2-cyclohexanediamine

Cis-1,2-diaminocyclohexane (500 mg, 4.38 mmol) was refluxed with salicylaldehyde (0.93 mL, 8.76 mmol) in ethanol (12 mL) for 2 h. The reaction mixture was kept in the refrigerator overnight, and the precipitated yellow crystals were collected by filtration and dried under vacuum. Yield: 1.15 g (82%). ^{1}H-NMR (400 MHz, $CDCl_3$) δ ppm: 8.27 (s, 2H), 7.23-7.18 (m, 2H), 7.16 (dd, J = 7.6, 1.7 Hz, 2H), 6.84 (dd, J = 8.3, 1.0 Hz, 2H), 6.77 (td, J = 7.5, 1.1 Hz, 2H), 3.55-3.53 (m, 2H), 1.97-1.77 (m, 4H), 1.74-1.65 (m, 2H), 1.56-1.46 (m, 2H). ^{13}C-NMR (101 MHz, $CDCl_3$) δ ppm: 164.3, 161.4, 132.4, 131.6, 118.9, 118.6, 117.1, 69.5, 30.8, 22.6. NMR data are in accordance with literature values.[2]

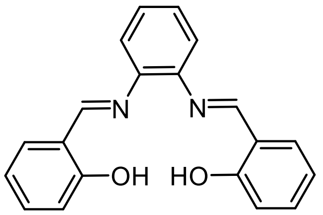

***N*,*N'*-Bis(salicylidene)-1,2-benzenediamine**

o-Phenylenediamine (1.08 g, 10 mmol) was dissolved in ethanol (25 mL). Salicylaldehyde (2.44 g, 20 mmol) dissolved in ethanol (25 mL) was added dropwise to the solution over 5 min and the reaction was heated to reflux for 2½ h. The mixture was allowed to cool to room temperature, and the precipitated orange crystals were collected by filtration, washed with cold ethanol and dried under vacuum. Yield: 822 mg (26%). ^{1}H-NMR (400 MHz, $CDCl_3$) δ ppm: 8.65 (s, 2H), 7.41-7.33 (m, 6H), 7.27-7.24 (m, 2H), 7.07 (d, J = 8.2, 1.0 Hz, 2H), 6.93 (td, J = 7.5, 1.1 Hz, 2H). ^{13}C-NMR (101 MHz, $CDCl_3$) δ ppm: 163.9, 161.5, 142.5, 133.7, 132.6, 127.9, 119.9, 119.3, 119.2, 117.7. NMR data are in accordance with literature values.[3]

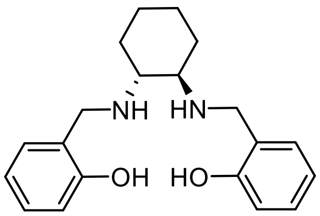

(1*R*,2*R*)-*N*,*N′*-Bis(2-hydroxybenzyl)-1,2-cyclohexanediamine

Sodium borohydride (247 mg, 6.5 mmol) was added over 30 min to a solution of (1*R*,2*R*)-*N*,*N′*-bis(salicylidene)-1,2-cyclohexanediamine (1.0 g, 3.1 mmol) in methanol (12 mL) at room temperature and the reaction mixture was stirred under reflux for 1 h. After cooling to room temperature, water (15 mL) was added and the mixture was extracted with dichloromethane (3 × 12 mL). The combined organic layers were evaporated to dryness and the residue purified by column chromatography (0-10% EtOAc/hexane) to afford the desired ligand as a yellow oil. Yield: 515 mg (51%). ^{1}H-NMR (400 MHz, $CDCl_3$) δ ppm: 7.17 (td, *J* = 7.8, 1.7 Hz, 2H), 6.98 (dd, *J* = 7.4, 1.6 Hz, 2H), 6.85-6.75 (m, 4H), 4.10-3.87 (m, 4H), 2.49-2.40 (m, 2H), 2.21-2.10 (m, 2H), 1.79-1.65 (m, 2H), 1.28-1.16 (m, 4H). ^{13}C-NMR (101 MHz, $CDCl_3$) δ ppm: 158.0, 129.0, 128.5, 122.9, 119.4, 116.6, 59.8, 49.7, 30.5, 24.3. NMR data are in accordance with literature values.[1]

Procedures for manganese(III) catalyst synthesis

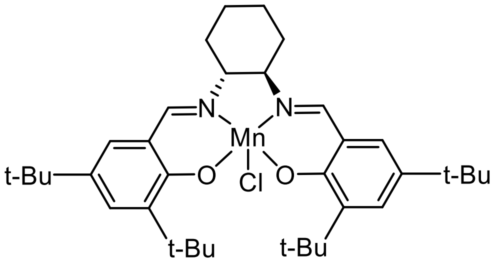

(1*R*,2*R*)-*N*,*N′*-Bis(3,5-di-*tert*-butylsalicylidene)-1,2-cyclohexanediaminomanganese(III) chloride (1)

$Mn(OAc)_2{\bullet}4H_2O$ (2.5 g, 10 mmol) was dissolved in EtOH (20 mL) and the mixture was heated to reflux temperature. A solution of (1*R*,2*R*)-*N*,*N′*-bis(3,5-di-*tert*-butylsalicylidene)-1,2-cyclohexanediamine (1.86 g, 3.33 mmol) in toluene (10 mL) is added in a slow stream over 45 min. The reaction mixture was stirred at reflux temperature for 3 h in the presence of air. A solution of saturated aqueous NaCl (4 mL) was added and the reaction mixture was allowed to cool to room temperature. The phases were separated and the organic solution was

washed with water (3x25 mL) followed by saturated aqueous NaCl (20 mL). The organic layer was dried (Na_2SO_4) and concentrated in vacuo. The dark brown solid was dissolved in dichloromethane (15 mL). Heptane (15 mL) was added and dichloromethane was removed by reduced pressure. After complete removal of dichloromethane, the brown slurry was stirred for 1 h at 5 ^{o}C. The brown solid was collected by filtration and dried under vacuum at 60 ^{o}C to yield the desired product as a dark brown powder. Yield: 1.79 g (85%). ESI-HRMS, *m/z* = 600.3456, $[C_{36}H_{53}MnN_2O_2+H]^+$, calc. 600.3482. FTIR, *v*/cm^{-1}: 2950 s, 1606 vs, 1534 m, 1432 w, 1388 w, 1312 m, 1251 m, 1174 m, 837 w, 749 w, 542 w.[4]

General procedure for synthesis of manganese complex 2-8

$MnCl_2{\bullet}4H_2O$, $MnBr_2$ or $Mn(OAc)_2{\bullet}4H_2O$ (3.30 mmol) and ligand (3.00 mmol) in EtOH (100 mL) were refluxed for 2 h in the presence of air. The reaction mixture was allowed to cool to room temperature and concentrated under reduced pressure yielding a dark brown solid, which was recrystallized from EtOH and dried under vacuum to give the desired product.

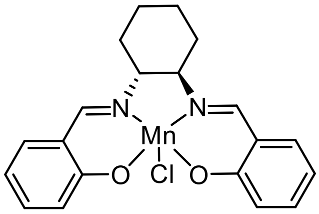

(1*R*,2*R*)-*N*,*N'*-Bis(salicylidene)-1,2-cyclohexanediaminomanganese(III) chloride (2)

Following the general procedure for synthesis of manganese complexes with $MnCl_2{\bullet}4H_2O$, the catalyst was obtained as black crystals. Yield: 688 mg (53%). ESI-HRMS, *m/z* = 433.0477 $[M+Na]^+$, calc. 433.0594. FTIR, *v*/cm^{-1}: 2912 m, 1617 vs, 1597 vs, 1549 s, 1469 m, 1435 s, 1302 s, 1247 s, 1219 s, 1080 m, 982 m, 846 w, 730 w. Anal. calc.: C, 58.48; H, 4.91; Cl, 8.63; Mn, 13.37; N, 6.82. Found: C, 58.30; H, 4.83; Cl, 8.61; Mn, 13.29; N, 6.49.[5] Trace metal analysis: Ag <2; Co <0.5; Cr <3; Cu <10; Fe <20; Mo <0.4; Ni <4; Zn <20; Au <1; Pd <1; Pt <1; Ir <1; Rh <1; Ru <1; Os <1 ppm.

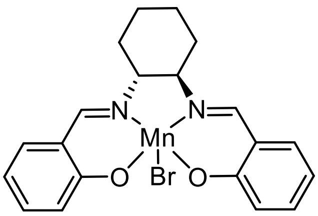

(1*R*,2*S*)-*N*,*N'*-Bis(salicylidene)-1,2-cyclohexanediaminomanganese(III) bromide (3)

Following the general procedure for synthesis of manganese complexes with $MnBr_2$, the catalyst was obtained as a brown powder. Yield: 137 mg (10%). ESI-HRMS, *m/z* = 376.0937, $[C_{20}H_{21}MnN_2O_2+H]^+$, calc. 376.0978. FTIR, ν/cm^{-1}: 2938 m, 2852 m, 1614 vs, 1540 m, 1441 m, 1306 w, 1202 m, 1149 m, 904 w, 858 w, 811 w, 752 m, 624 m, 568 m, 424 w.[6]

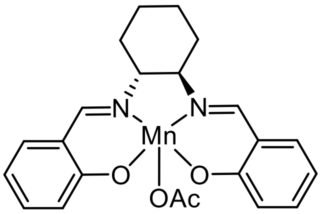

(1*R*,2*S*)-*N*,*N'*-Bis(salicylidene)-1,2-cyclohexanediaminomanganese(III) acetate (4)

Following the general procedure for synthesis of manganese complexes with $Mn(OAc)_2{\bullet}4H_2O$, the catalyst was obtained as a brown powder. Yield: 520 mg (40%). ESI-HRMS, *m/z* = 375.0935 $[C_{20}H_{20}MnN_2O_2]^+$, calc. 375.0905. FTIR, ν/cm^{-1}: 2933 w, 2862 w, 1619 s, 1537 vs, 1441 s, 1308 s, 1199 m, 1147 w, 907 w, 809 w, 760 m, 622 w, 567 w, 425 w.[7]

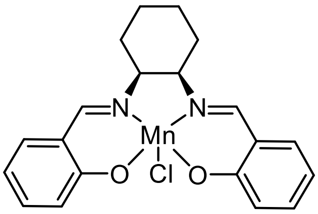

(1*S*,2*R*)-*N*,*N'*-Bis(salicylidene)-1,2-cyclohexanediaminomanganese(III) chloride (5)

Following the general procedure for synthesis of manganese complexes with $MnCl_2{\bullet}4H_2O$, the catalyst was obtained as black crystals. Yield: 650 mg (50%). ESI-HRMS, *m/z* = 433.0520 $[M+Na]^+$, calc. 433.0594. FTIR, ν/cm^{-1}: 2925 m, 1619 vs, 1599 vs, 1543 s, 1445 s, 1400 w, 1318 s, 1279 s, 1196 w, 1151 w, 902 w, 814 w, 747 s, 603 m, 432 w. Anal. calc.: C, 58.48; H, 4.91; Cl, 8.63; Mn, 13.37; N, 6.82. Found: C, 57.94; H, 4.97; Cl, 8.47; Mn, 13.51; N, 6.53.

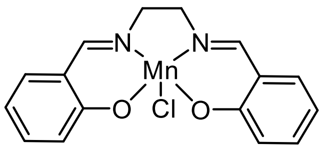

_N,N′_-Bis(salicylidene)ethylenediaminomanganese(III) chloride (6)

Following the general procedure for synthesis of manganese complexes with $MnCl_2$•$4H_2O$, the catalyst was obtained as black crystals. Yield: 438 g (41%). ESI-HRMS, *m/z* = 322.0471, $[C_{16}H_{15}MnN_2O_2+H]^+$, calc. 322.0509. FTIR, *v*/cm^{-1}: 1617 vs, 1595 vs, 1533 s, 1442 s, 1389 m, 1322 m, 1300 w, 1274 s, 1197 m, 1147 m, 1125 m, 886 w, 796 w, 756 s, 621 w, 458 w.[5]

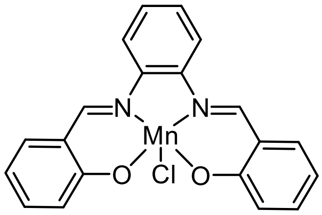

_N,N′_-Bis(salicylidene)-1,2-benzenediaminomanganese(III) chloride (7)

N,N′-Bis(salicylidene)-1,2-benzenediamine (500 mg, 1.60 mmol) was dissolved in EtOH (15 mL). $Mn(OAc)_2$•$4H_2O$ (780 mg, 4.50 mmol) was added to the solution and the mixture was refluxed for 1 h. LiCl (200 mg, 4.7 mmol) was added and the reaction was refluxed for an additional 30 min. The mixture was allowed to cool to room temperature and the precipitated brown solid was collected, washed with cold EtOH and dried under vaccum. Yield: 226 mg (35%). ESI-HRMS, *m/z* = 370.0471 $[C_{20}H_{15}MnN_2O_2+H]^+$, calc. 370.0508. FTIR, *v*/cm^{-1}: 1605 vs, 1580 s, 1538 vs, 1462 s, 1438 s, 1383 m, 1290 s, 1179 m, 1153 m, 1035 w, 914 w, 750 s, 531 w.

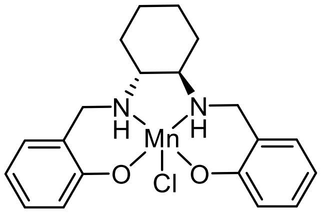

(1*R*,2*R*)-*N,N′*-Bis(2-hydroxybenzyl)-1,2-cyclohexanediaminomanganese(III) chloride (8)

Following the general procedure for synthesis of manganese complexes with $MnCl_2$•$4H_2O$, the catalyst was obtained as a brown powder. Yield: 633 mg (51%). FTIR, *v*/cm^{-1}: 3344 s, 2937 m, 1602 vs, 1479 m, 1448 s, 1261 s, 1239 m, 754 m.

Characterization data for imines

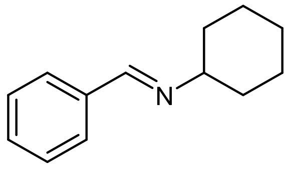

N-Benzylidenecyclohexylamine (Table 3, entry 1)

Following the general procedure for imine synthesis, the product was isolated as a yellow liquid. Yield: 166 mg (89%). ^{1}H-NMR (400 MHz, $CDCl_3$) δ ppm: 8.32 (s, 1H), 7.74-7.72 (m, 2H), 7.41-7.38 (m, 3H), 3.24-3.17 (m, 1H), 1.85-1.28 (m, 10H). ^{13}C-NMR (101 MHz, $CDCl_3$) δ ppm: 158.4, 136.4, 130.1, 128.3, 127.9, 69.8, 34.2, 25.5, 24.7. MS: *m/z* = 187 $[M]^+$. NMR data are in accordance with literature values.[8]

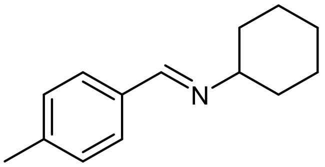

N-(4-Methylbenzylidene)-cyclohexylamine (Table 3, entry 2)

Following the general procedure for imine synthesis, the product was isolated as a yellow liquid. Yield: 141 mg (70%). ^{1}H-NMR (400 MHz, $CDCl_3$) δ ppm: 8.28 (s, 1H), 7.62 (d, *J* = 7.9 Hz, 2H), 7.20 (d, *J* = 7.9 Hz, 2H), 3.21-3.14 (m, 1H), 2.37 (s, 3H), 1.86-1.24 (m, 10H). ^{13}C-NMR (101 MHz, $CDCl_3$) δ ppm: 158.7, 140.7, 134.0, 129.3, 128.2, 70.1, 34.5, 25.8, 25.0, 21.6. MS: *m/z* = 201 $[M]^+$. NMR data are in accordance with literature values.[9]

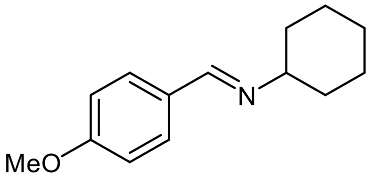

N-(4-Methoxybenzylidene)-cyclohexylamine (Table 3, entry 3)

Following the general procedure for imine synthesis, the product was isolated as a yellow liquid. Yield: 180 mg (83%). ^{1}H-NMR (400 MHz, $CDCl_3$) δ ppm: 8.24 (s, 1H), 7.66 (d, *J* = 8.7 Hz, 2H), 6.90 (d, *J* = 8.8 Hz, 2H), 3.82 (s, 3H), 3.18-3.11 (m, 1H), 1.86-1.16 (m, 10H). ^{13}C-NMR (101 MHz, $CDCl_3$) δ ppm: 161.5, 158.0, 132.0, 129.7, 114.0, 70.0, 55.4, 34.6, 25.8, 25.0. MS: *m/z* = 217 $[M]^+$. NMR data are in accordance with literature values.[8]

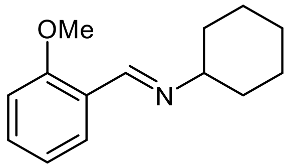

N-(2-Methoxybenzylidene)-cyclohexylamine (Table 3, entry 4)

Following the general procedure for imine synthesis, the product was isolated as a yellow liquid. Yield: 195 mg (90%). ^{1}H-NMR (400 MHz, $CDCl_3$) δ ppm: 8.74 (s, 1H), 7.94 (dd, *J* = 7.7, 1.8 Hz, 1H), 7.45-7.30 (m, 1H), 7.02-6.82 (m, 2H), 3.86 (s, 3H), 3.25-3.17 (m, 1H), 1.83-1.22 (m, 10H). ^{13}C-NMR (101 MHz, $CDCl_3$) δ ppm: 158.7, 154.7, 131.6, 127.5, 125.2, 120.9, 111.0, 70.4, 55.6, 34.6, 25.8, 25.0. MS: *m/z* = 217 $[M]^+$. NMR data are in accordance with literature values.[10]

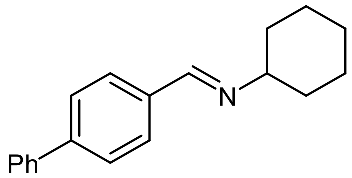

N-(4-Phenylbenzylidene)-cyclohexylamine (Table 3, entry 5)

Following the general procedure for imine synthesis, the product was isolated as a pale yellow solid. Yield: 158 mg (60%). ^{1}H-NMR (400 MHz, $CDCl_3$) δ ppm: 8.36 (s, 1H), 7.87-7.77 (m, 2H), 7.67-7.56 (m, 4H), 7.49-7.30 (m, 3H), 3.27-3.18 (m, 1H), 1.87-1.23 (m, 10H). ^{13}C-NMR (101 MHz, $CDCl_3$) δ ppm: 158.3, 143.2, 140.7, 135.7, 128.9, 128.6, 127.8, 127.4, 127.3, 70.2, 34.5, 25.8, 25.0. MS: *m/z* = 263 $[M]^+$. NMR data are in accordance with literature values.[11]

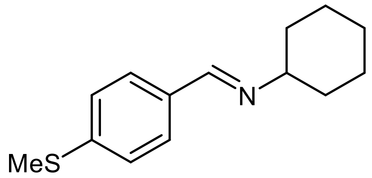

N-(4-Methylthiobenzylidene)-cyclohexylamine (Table 3, entry 6)

Following the general procedure for imine synthesis, the product was isolated as a pale yellow solid. Yield: 140 mg (60%). ^{1}H-NMR (400 MHz, $CDCl_3$) δ ppm: 8.25 (s, 1H), 7.65 (d, *J* = 8.4 Hz, 2H), 7.24 (m, 2H), 3.21-3.14 (m, 1H), 2.50 (s, 3H), 1.86-1.19 (m, 10H). ^{13}C-NMR (101 MHz,

$CDCl_3$) δ ppm: 158.2, 129.0, 128.6, 126.0, 123.8, 70.0, 34.5, 25.8, 25.0, 15.5. MS: *m/z* = 234 $[M]^+$. NMR data are in accordance with literature values.[11]

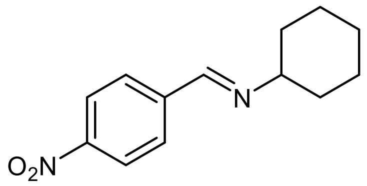

N-(4-Nitrobenzylidene)-cyclohexylamine (Table 3, entry 7)

Following the general procedure for imine synthesis, the product was isolated as a pale yellow solid. Yield: 162 mg (70%). ^{1}H-NMR (400 MHz, $CDCl_3$) δ ppm: 8.38 (s, 1H), 8.25 (d, *J* = 8.8 Hz, 2H), 7.89 (d, *J* = 8.8 Hz, 2H), 3.31-3.24 (m, 1H), 1.88-1.24 (m, 10H). ^{13}C-NMR (101 MHz, $CDCl_3$) δ ppm: 156.4, 149.0, 142.3, 128.8, 123.9, 70.3, 34.3, 25.7, 24.7. MS: *m/z* = 232 $[M]^+$. NMR data are in accordance with literature values.[12]

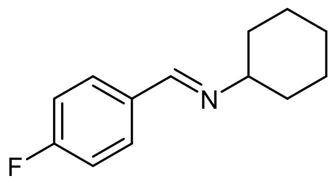

N-(4-Fluorobenzylidene)-cyclohexylamine (Table 3, entry 8)

Following the general procedure for imine synthesis, the product was isolated as a yellow liquid. Yield: 150 mg (73%). ^{1}H-NMR (400 MHz, $CDCl_3$) δ ppm: 8.27 (s, 1H), 7.72 (dd, *J* = 8.7, 5.6 Hz, 2H), 7.07 (t, *J* = 8.7 Hz, 2H), 3.22-3.14 (m, 1H), 1.84-1.24 (m, 10H). ^{13}C-NMR (101 MHz, $CDCl_3$) δ ppm: 165.5 (d, *J* = 253 Hz), 157.3, 133.0, 130.1 (d, *J* = 10 Hz), 115.8 (d, *J* = 20 Hz) 70.0, 34.5, 25.8, 24.9. MS: *m/z* = 205 $[M]^+$. NMR data are in accordance with literature values.[8]

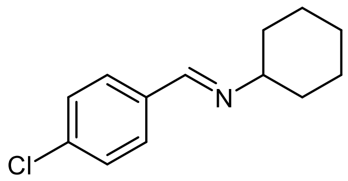

N-(4-Chlorobenzylidene)-cyclohexylamine (Table 3, entry 9)

Following the general procedure for imine synthesis, the product was isolated as a white solid. Yield: 186 mg (85%). ^{1}H-NMR (400 MHz, $CDCl_3$) δ ppm: 8.27 (s, 1H), 7.66 (d, *J* = 8.5 Hz, 2H),

7.36 (d, J = 8.5 Hz, 2H), 3.23-3.16 (m, 1H), 1.85-1.24 (m, 10H). ^{13}C-NMR (101 MHz, $CDCl_3$) δ ppm: 157.4, 136.4, 135.2, 129.4, 128.9, 70.1, 34.4, 25.8, 24.9. MS: *m/z* = 222 $[M]^+$. NMR data are in accordance with literature values.[13]

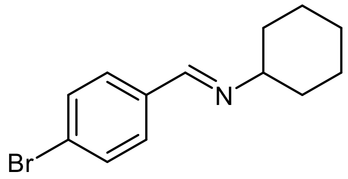

N-(4-Bromobenzylidene)-cyclohexylamine (Table 3, entry 10)

Following the general procedure for imine synthesis, the product was isolated as a pale yellow solid. Yield: 162 mg (61%). ^{1}H-NMR (400 MHz, $CDCl_3$) δ ppm: 8.25 (s, 1H), 7.64-7.46 (m, 4H), 3.22-3.15 (m, 1H), 1.83-1.24 (m, 10H). ^{13}C-NMR (101 MHz, $CDCl_3$) δ ppm: 157.4, 135.6, 131.8, 129.6, 124.7, 70.1, 34.4, 25.7, 24.9. MS: *m/z* = 265 $[M]^+$. NMR data are in accordance with literature values.[11]

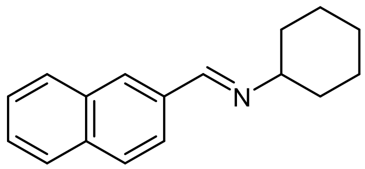

N-(2-Naphthalenylmethylene)-cyclohexylamine (Table 3, entry 11)

Following the general procedure for imine synthesis, the product was isolated as a white solid. Yield: 168 mg (71%). ^{1}H-NMR (400 MHz, $CDCl_3$) δ ppm: 8.47 (s, 1H), 8.04-7.99 (m, 2H), 7.90-7.83 (m, 3H), 7.55-7.44 (m, 2H), 3.30-3.23 (m, 1H), 1.88-1.63 (m, 7H), 1.46-1.25 (m, 3H). ^{13}C-NMR (101 MHz, $CDCl_3$) δ ppm: 158.7, 134.6, 134.2, 133.1, 129.5, 128.6, 128.4, 127.8, 126.9, 126.4, 124.1, 70.1, 34.4, 25.7, 24.9. MS: *m/z* = 237 $[M]^+$. NMR data are in accordance with literature values.[14]

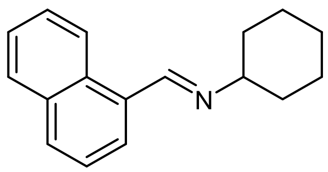

N-(1-Naphthalenylmethylene)-cyclohexylamine (Table 3, entry 12)

Following the general procedure for imine synthesis, the product was isolated as a yellow liquid. Yield: 142 mg (60%). ^{1}H-NMR (400 MHz, $CDCl_3$) δ ppm: 9.00 (s, 1H), 8.91 (d, *J* = 8.4 Hz, 1H), 7.93-7.86 (m, 3H), 7.63-7.49 (m, 3H), 3.36-3.29 (m, 1H), 1.95-1.66 (m, 7H), 1.50-1.30 (m, 3H). ^{13}C-NMR (101 MHz, $CDCl_3$) δ ppm: 158.0, 133.9, 132.2, 131.4, 130.7, 128.7, 128.4, 127.0, 126.0, 125.4, 124.4, 71.0, 34.7, 25.8, 24.9. MS: *m/z* = 237 $[M]^+$. NMR data are in accordance with literature values.[14]

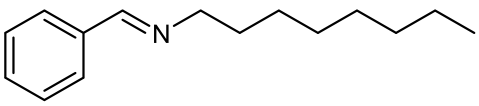

***N*-Benzylidene-octylamine (Table 4, entry 1)**

Following the general procedure for imine synthesis, the product was isolated as a yellow liquid. Yield: 195 mg (90%). ^{1}H-NMR (400 MHz, $CDCl_3$) δ ppm: 8.27 (s, 1H), 7.78-7.68 (m, 2H), 7.42-7.39 (m, 3H), 3.61 (td, *J* = 7.1, 1.4 Hz, 2H), 1.72-1.68 (m, 2H), 1.37-1.27 (m, 10H), 0.91-0.82 (m, 3H). ^{13}C-NMR (101 MHz, $CDCl_3$) δ ppm: 160.8, 136.5, 130.6, 128.7, 128.1, 62.0, 32.0, 31.1, 29.6, 29.4, 27.5, 22.8, 14.3. MS: *m/z* = 216 $[M-H]^+$. NMR data are in accordance with literature values.[15]

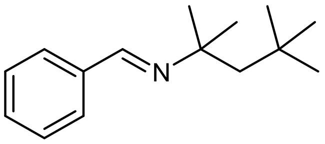

***N*-Benzylidene-*tert*-octylamine (Table 4, entry 2)**

Following the general procedure for imine synthesis, the product was isolated as a clear liquid. Yield: 139 mg (69%). ^{1}H-NMR (400 MHz, $CDCl_3$) δ ppm: 8.25 (s, 1H), 7.83-7.68 (m, 2H), 7.44-7.38 (m, 3H), 1.71 (s, 2H), 1.34 (s, 6H), 0.97 (s, 9H). ^{13}C-NMR (101 MHz, $CDCl_3$) δ ppm: 154.6, 137.5, 130.1, 128.7, 128.1, 61.2, 56.7, 32.2, 32.0, 29.8. MS: *m/z* = 216 $[M-H]^+$. NMR data are in accordance with literature values.[16]

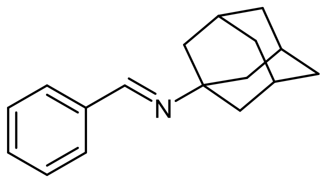

N-Benzylidene-1-adamantanylamine (Table 4, entry 3)

Following the general procedure for imine synthesis, the product was isolated as a white solid. Yield: 210 mg (88%). ^{1}H-NMR (400 MHz, $CDCl_3$) δ ppm: 8.29 (s, 1H), 7.77 (bs, 2H), 7.48-7.34 (m, 3H), 2.18 (s, 3H), 1.84 (s, 6H), 1.74 (m, 6H). ^{13}C-NMR (101 MHz, $CDCl_3$) δ ppm: 155.1, 137.2, 130.3, 128.6, 128.0, 57.6, 43.2, 36.7, 29.7. MS: *m/z* = 239 $[M]^+$. NMR data are in accordance with literature values.[17]

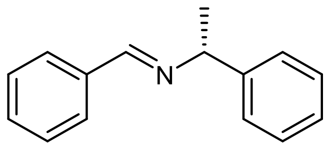

(*R*)-*N*-Benzylidene-1-phenylethylamine (Table 4, entry 4)

Following the general procedure for imine synthesis, the product was isolated as a clear liquid. Yield: 138 mg (66%). $[\alpha]_D^{20}$ = -68.2 (*c* = 1.58, $CHCl_3$) (ref.[18] $[\alpha]_D^{27}$ = -64.7 (c = 1.0, $CHCl_3$). ^{1}H-NMR (400 MHz, $CDCl_3$) δ ppm: 8.26 (s, 1H), 7.70-7.67 (m, 2H), 7.40-7.06 (m, 8H), 4.44 (q, *J* = 6.6 Hz, 1H), 1.50 (d, *J* = 6.7 Hz, 3H). ^{13}C-NMR (101 MHz, $CDCl_3$) δ ppm: 159.5, 145.3, 136.5, 130.6, 128.6, 128.5, 128.3, 126.9, 126.7, 69.8, 25.0. MS: *m/z* = 209 $[M]^+$. NMR data are in accordance with literature values.[18]

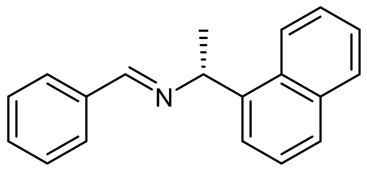

(*R*)-*N*-Benzylidene-1-(1-naphthyl)ethylamine (Table 4, entry 5)

Following the general procedure for imine synthesis, the product was isolated as a white solid. Yield: 215 mg (83%). $[\alpha]_D^{20}$ = -232.6 (*c* = 1.00, $CHCl_3$) (ref.[18] $[\alpha]_D^{27}$ = -250.3 (c = 1.04, $CHCl_3$). ^{1}H-NMR (400 MHz, $CDCl_3$) δ ppm: 8.45 (s, 1H), 8.28 (d, *J* = 8.6 Hz, 1H), 7.92-7.74 (m, 5H), 7.60-7.38 (m, 6H), 5.38 (q, *J* = 6.6 Hz, 1H), 1.76 (d, *J* = 6.6 Hz, 3H). ^{13}C-NMR (101 MHz, $CDCl_3$) δ ppm: 159.8, 141.3, 136.6, 134.1, 130.8, 130.7, 129.1, 128.7, 128.4, 127.5, 125.9, 125.8, 125.4,

124.2, 123.8, 65.7, 24.7. MS: *m/z* = 259 $[M]^+$. NMR data are in accordance with literature values.[18]

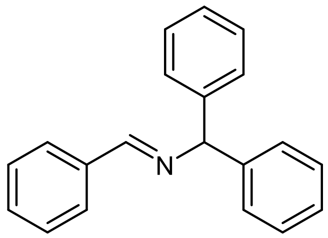

N-Benzylidene-1,1-diphenylmethylamine (Table 4, entry 6)

Following the general procedure for imine synthesis, the product was isolated as a white solid. Yield: 198 mg (73%). ^{1}H-NMR (400 MHz, $CDCl_3$) δ ppm: 8.35 (s, 1H), 7.82-7.67 (m, 2H), 7.36-7.29 (m, 7H), 7.26-7.22 (m, 4H), 7.18-7.13 (m, 2H), 5.52 (s, 1H). ^{13}C-NMR (101 MHz, $CDCl_3$) δ ppm: 160.9, 144.0, 136.5, 130.9, 128.7, 128.6, 128.6, 127.8, 127.1, 78.0. MS: *m/z* = 271 $[M]^+$. NMR data are in accordance with literature values.[19]

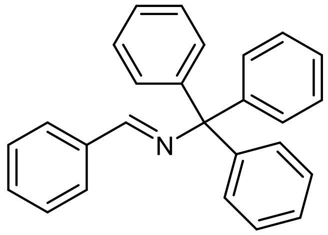

N-Benzylidene-1,1,1-triphenylmethylamine (Table 4, entry 7)

Following the general procedure for imine synthesis, the product was isolated as a white solid. Yield: 66 mg (19%). ^{1}H-NMR (400 MHz, $CDCl_3$) δ ppm: 7.80-7.73 (m, 2H), 7.36-7.33 (m, 2H), 7.26-7.10 (m, 17H). ^{13}C-NMR (101 MHz, $CDCl_3$) δ ppm: 159.8, 146.0, 136.9, 130.9, 129.9, 128.7, 128.7, 127.9, 126.9, 78.4. MS: *m/z* = 347 $[M]^+$. NMR data are in accordance with literature values.[20]

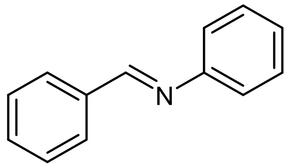

N-Benzylideneaniline (Table 4, entry 8)

Following the general procedure for imine synthesis, the product was isolated as a white solid. Yield: 134 mg (74%). ^{1}H-NMR (400 MHz, $CDCl_3$) δ ppm: 8.38 (s, 1H), 7.86-7.82 (m, 2H), 7.42-7.38 (m, 3H), 7.34-7.30 (m, 2H), 7.18-7.12 (m, 3H). ^{13}C-NMR (101 MHz, $CDCl_3$) δ ppm: 160.6, 131.6, 129.9, 129.3, 129.1 129.0, 128.9, 126.1, 121.0. MS: *m/z* = 187 $[M]^+$. NMR data are in accordance with literature values.[21]

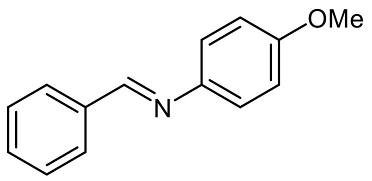

N-Benzylidene-p-anisidine (Table 4, entry 9)

Following the general procedure for imine synthesis, the product was isolated as a white solid. Yield: 133 mg (63%). ^{1}H-NMR (400 MHz, $CDCl_3$) δ ppm: 8.40 (s, 1H), 7.84-7.78 (m, 2H), 7.41-7.35 (m, 3H), 7.17 (d, J = 8.8 Hz, 2H), 6.86 (d, J = 8.9 Hz, 2H), 3.75 (s, 3H). ^{13}C-NMR (101 MHz, $CDCl_3$) δ ppm: 158.6, 158.4, 145.0, 136.5, 131.2, 128.9, 128.7, 122.3, 114.5, 55.6. MS: *m/z* = 211 $[M]^+$. NMR data are in accordance with literature values.[21]

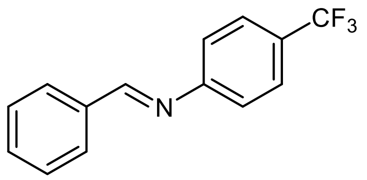

N-Benzylidene-4-(trifluoromethyl)aniline (Table 4, entry 10)

Following the general procedure for imine synthesis, the product was isolated as a white solid. Yield: 154 mg (62%). ^{1}H-NMR (400 MHz, $CDCl_3$) δ ppm: 8.44 (s, 1H), 7.94-7.92 (m, 2H), 7.66-7.64 (m, 2H), 7.54-7.48 (m, 3H), 7.28-7.26 (m, 2H). ^{13}C-NMR (101 MHz, $CDCl_3$) δ ppm: 162.2, 155.2, 135.7, 132.2, 129.3, 129.1, 127.8 (q, J = 33 Hz), 126.5 (q, J = 4 Hz), 125.8 (q, J = 270 Hz), 121.1. MS: *m/z* = 248 $[M-H]^+$.

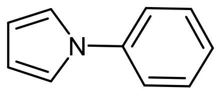

1-Phenylpyrrole (Scheme 1)

Following the general procedure for pyrrole synthesis, the product was isolated as a white solid. Yield: 67 mg (47%). ^{1}H-NMR (400 MHz, $CDCl_3$) δ ppm: 7.49-7.43 (m, 4H), 7.31-7.27 (m, 1H), 7.14 (t, J = 2.2 Hz, 2H), 6.40 (t, J = 2.2 Hz, 2H). ^{13}C-NMR (101 MHz, $CDCl_3$) δ ppm: 140.9, 129.7, 125.7, 120.7, 119.4, 110.5. MS: m/z = 143 $[M]^+$. NMR data are in accordance with literature values.[22]

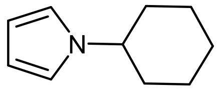

1-Cyclohexylpyrrole (Scheme 1)

Following the general procedure for pyrrole synthesis, the product was isolated as a yellow liquid. Yield: 92 mg (62%). ^{1}H-NMR (400 MHz, $CDCl_3$) δ ppm: 6.76 (t, J = 2.1 Hz, 2H), 6.16 (t, J = 2.2 Hz, 2H), 3.87-3.79 (m, 1H), 2.15-2.10 (m, 2H), 1.93-1.88 (m, 2H), 1.79-1.19 (m, 6H). ^{13}C-NMR (101 MHz, $CDCl_3$) δ ppm: 118.4, 107.5, 58.8, 34.8, 25.9, 25.5. MS: m/z = 149 $[M]^+$. NMR data are in accordance with literature values.[23]

Computational details

All calculations were performed with the Jaguar 9.9 program package by Schrodinger LLC. Geometry optimizations were performed using the empirically corrected B3LYP-D3 functional and the LACVP** basis set and core potential on Mn, which employs the 6-31G** basis on all other atoms. All calculations were performed using the unrestricted formalism, and the multiplicity of all Mn complexes was assumed to be the high spin quintet. Analytical Hessians were computed for all intermediates and transition states to confirm that they have none and one imaginary vibrational frequency, respectively. The solvation free energies of all Mn containing structures were calculated using the PBF solver in Jaguar with standard parameters for benzene, using the same basis set and functional as in the geometry optimization. For the molecules that did not contain Mn, we used the SM8 solvation model, and the basis set was slightly smaller, 6-31G*, since this will enable CM4 charges which generate higher accuracy solvation free energies. Finally, the electronic energy was calculated using the larger LACV3P**++ for all structures. The final free energies were calculated as the sum G = E(LACV3P**++) + G_{solv} + ZPE + ΔH + TS where the temperature was set to the boiling point of toluene (383 K). The solvation energies were calculated at standard state (1 atm). The Hammett study was performed by calculating the difference in G = E(LACV3P**++) + G_{solv} between structure **15** and **16ts** ($\Delta G^{\ddagger}$), where the ratio of the rate constants k_x/k_H was calculated by k_x/k_H = EXP[($\Delta G^{\ddagger}$(H) - $\Delta G^{\ddagger}$(x))/RT] where T was 383 K and R is the ideal gas constant. The kinetic isotope effect was calculated using G = E(LACV3P**++) + G_{solv} + ZPE + ΔH + TS for **15** and **16ts**, where the weight for the benzylic hydrogen atoms were set to 1 and 2 in the calculation of the vibrational frequencies, to represent hydrogen and deuterium, respectively. This gave giving $\Delta G^{\ddagger}$(prot) and giving $\Delta G^{\ddagger}$(deut) and the ratio k_{deut}/k_{prot} = EXP[($\Delta G^{\ddagger}$(deut) - $\Delta G^{\ddagger}$(prot))/RT] at 383 K was used to calculate the kinetic isotope effect. This approximates the proton and deuterium to be classical particles, in accordance with the experimental result, which did not give any indication of non-classical behavior. The turnover frequency is the reaction rate (v) divided by the catalyst concentration. For the reaction mechanism in this report we expect the turnover limiting step to be from **15** to **16ts**, for which the rate is expected to be $v_{15\text{-}16ts} = k_r$[**15**]. Given that the reactants are present in great excess we assume that [**15**] = [Mn], from which we get that TOF = v/[Mn] = $v_{15\text{-}16ts}$ /[Mn]= kr. By applying transition state theory, we calculate $k_r = k_BT/h$ EXP[$\Delta G^{\ddagger}$/RT], where k_B = Boltzmann's constant and h = Planck's constant.

Activation of catalyst and catalytic cycle

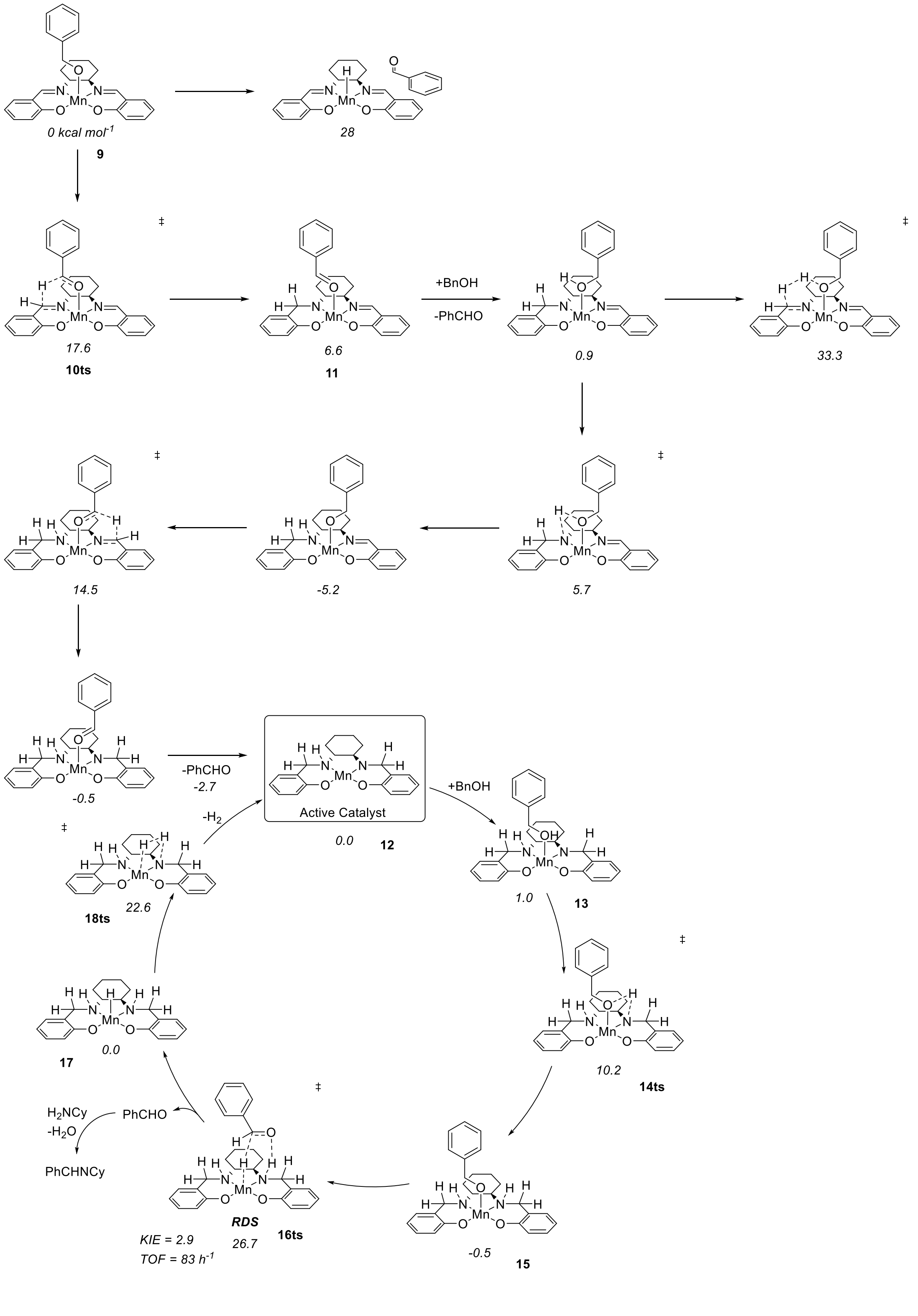

Energies of complexes

	E Lacv3p**++	ZPE	H	S	Solv
**BzOH**	-346.8833855	83.641	5.096	84.812	-4.494
**PhCHO**	-345.6767939	69.174	4.519	79.302	-4.014
**9**	-1484.805438	306.734	17.709	184.73	-2.936
**10ts**	-1484.77053	304.402	17.663	184.468	-4.9275
**11**	-1484.788023	306.589	18.401	193.097	-4.4387
**12**	-1140.298265	251.491	13.699	155.526	-5.9771
**13**	-1487.222379	336.947	18.821	194.122	-4.6369
**14ts**	-1487.207963	336.947	18.821	194.122	-4.4784
**15**	-1487.228134	337.537	18.567	191.927	-3.7412
**16ts**	-1487.178719	333.874	18.396	190.993	-4.0447
**17**	-1141.488196	264.049	14.007	155.271	-6.0202
**18ts**	-1141.447075	260.195	13.875	154.361	-5.5868

Cartesian coordinates of complexes

9

C1	-0.1177589712	0.2506909521	0.8216093611
C2	-1.5610935887	0.4618598960	0.3352035125
C3	-2.4044945177	1.2150061093	1.3742315250
C4	-2.3957414414	0.4877911392	2.7288854624
C5	-0.9580604430	0.3124306834	3.2239188920
C6	-0.0932150664	-0.4614308730	2.1813599814
H7	0.3944684346	1.2169104497	0.9240608993
H8	0.4370229035	-0.3302087236	0.0765374863
H9	-2.0215915380	-0.5164666188	0.1371853790
H10	-1.5557641989	1.0034315931	-0.6174112276
H11	-2.0019553724	2.2286509724	1.5054294203
H12	-3.4350725525	1.3279709360	1.0193532329
H13	-2.9805162025	1.0491935997	3.4671074361
H14	-2.8725913242	-0.4963080601	2.6152842378
H15	-0.4933197953	1.2970632450	3.3648899746
H16	-0.5520544396	-1.4575597425	2.0757941272
N17	1.2254122423	-0.6556190367	2.7928567910
N18	-0.7785839845	-0.3940411080	4.4946795690
Mn19	1.1866613874	-0.4831892185	4.9533337574

C20	2.3148191467	-0.7757775424	2.1181792121
H21	2.2878640945	-0.7562369558	1.0222961943
C22	-1.7307403229	-0.9417258133	5.1772877643
H23	-2.7695511037	-0.7536273400	4.8928299946
C24	-1.2631792736	-3.7749671747	8.2825248909
C25	-0.1650583406	-3.4474235254	7.5104958113
C26	-0.2466151959	-2.4540106000	6.5010471303
C27	-1.5253744428	-1.8406056155	6.2785697471
C28	-2.6328297399	-2.2147655646	7.0739523003
C29	-2.5124442688	-3.1549549476	8.0775668411
H30	-1.1599756228	-4.5266815062	9.0616110635
H31	-3.5943742954	-1.7420826090	6.8819272187
C32	6.2826055164	-1.2983974594	3.5600010877
C33	5.2734619774	-1.1050104512	4.4855537759
C34	3.9243713818	-0.9252867791	4.0898633166
C35	3.6369310566	-0.9445204574	2.6829259983
C36	4.6989076060	-1.1354504848	1.7681846455
C37	6.0049676698	-1.3147064315	2.1819861507
H38	7.3032886184	-1.4390221931	3.9073464775
H39	4.4649518868	-1.1447055030	0.7051122339
O40	3.0385818973	-0.7624769143	5.0444041533
O41	0.8239639211	-2.1673846878	5.8088473297
H42	0.8003932160	-3.9186317352	7.6628739190
H43	5.4780850428	-1.0922145590	5.5514406004
H44	6.7996756510	-1.4652359721	1.4584657650
H45	-3.3672997573	-3.4240196745	8.6898750255
O46	1.2657837451	1.3573730450	5.0331348488
C47	1.8875910002	1.8643723001	6.1995563280
H48	1.6907417641	2.9458616857	6.2465544918
H49	2.9778678658	1.7194292324	6.1484378855
C50	0.1852090073	-0.2706734104	9.5582780762
C51	1.4340419473	-0.6368553973	9.0571377980
C52	2.0076754878	0.0822101071	8.0054316555
C53	1.3392986172	1.1770749105	7.4406302564

C54	0.0832356819	1.5350738200	7.9510811259
C55	-0.4892026790	0.8204947286	9.0024740518
H56	-0.2683933077	-0.8394690181	10.3649684684
H57	1.9563688302	-1.4949732174	9.4708827680
H58	2.9575348887	-0.2305490323	7.5810353934
H59	-0.4480892932	2.3741394634	7.5072878049
H60	-1.4647073871	1.1080672345	9.3862411633

10ts

C95	0.0000000000	0.0000000000	0.0000000000
H99	0.0000000000	0.0000000000	1.3746486309
C23	0.7201766111	0.0000000000	2.5129226361
C1	2.1052967868	4.5456086576	1.4654573754
C2	0.8149654238	5.0700663050	2.1201727109
C3	0.4911324465	4.3135570692	3.4161233491
C4	0.3956964302	2.7975101312	3.1759534713
C5	1.6949610566	2.2752683265	2.5481030869
C6	1.9868536304	3.0370449807	1.2380472161
H7	2.9699725425	4.7580974311	2.1099011918
H8	2.2702698152	5.0682237056	0.5161336214
H9	-0.0175625964	4.9475928405	1.4136244634
H10	0.9078209210	6.1441288951	2.3160050991
H11	1.2756402622	4.5132602271	4.1593859244
H12	-0.4480959991	4.6808672960	3.8445947967
H13	0.1993741539	2.2837906378	4.1236609332
H14	-0.4464372327	2.5824818496	2.5037427375
H15	2.5217134906	2.5035523485	3.2403280306
H16	1.1387990563	2.8355882048	0.5730450333
N17	3.1280091889	2.3538552859	0.6038314500
N18	1.7716059122	0.8322156956	2.2693135388
Mn19	2.8925392699	0.3596501378	0.7338645515
C21	4.0635716925	2.9858319967	-0.0341480180
H22	4.0585408581	4.0777936476	-0.0192707342
H24	-0.0515595288	0.3720433108	3.1906336530

C25	1.2966153954	-4.2258244321	2.8913930984
C26	2.1573521087	-3.4900700054	2.0862644186
C27	1.9969851215	-2.0972291823	1.9427313474
C28	0.9523235225	-1.4588496662	2.6587941806
C29	0.0904357784	-2.2235545103	3.4565859734
C30	0.2524917155	-3.5993436967	3.5840741383
H31	1.4363883926	-5.3004524641	2.9778635959
H33	-0.7153087067	-1.7207126564	3.9884865142
C35	7.1419690774	1.3170137275	-2.4034686762
C36	6.2313975345	0.4642872637	-1.8087525189
C37	5.1891977931	0.9648230508	-0.9877780563
C38	5.1129532031	2.3832280603	-0.8002063092
C39	6.0639662355	3.2268765966	-1.4248493862
C40	7.0705795009	2.7133242533	-2.2164610106
H41	7.9264091465	0.9004569450	-3.0306367660
H43	5.9838704668	4.3015663986	-1.2730494069
O45	4.3507666196	0.1206434551	-0.4507055989
O46	2.8173156711	-1.4433996972	1.1257228132
H44	2.9636433043	-3.9636146758	1.5350458420
H45	6.2783052285	-0.6103024303	-1.9517555327
H46	7.7918415586	3.3697969384	-2.6918247348
H47	-0.4189107746	-4.1761806822	4.2125066102
H97	-0.0365082373	-1.1038058077	-0.0502854237
O99	1.0982061952	0.5895774997	-0.3450564077
C98	-3.7537744488	1.9904977426	-0.6300354641
C99	-2.5565781365	2.6441766743	-0.9315559095
C100	-1.3371469458	1.9929361661	-0.7479136207
C101	-1.3052877691	0.6818871903	-0.2562013612
C102	-2.5095954787	0.0279738106	0.0382747751
C103	-3.7273751928	0.6779581467	-0.1483392385
H104	-4.7038089202	2.4974420277	-0.7758108617
H105	-2.5752478258	3.6594591764	-1.3194377029
H106	-0.3977009597	2.4718651147	-1.0047296561
H107	-2.4840709475	-0.9909819993	0.4200434800

H108	-4.6571099131	0.1635444262	0.0796796354

11

C95	0.4961305159	-0.3310480687	-0.5627878649
H99	0.1153603309	0.8892025330	2.0837836119
C23	0.8908806663	0.4278933858	2.7303437704
C1	2.4299537533	4.8054656834	1.8200576075
C2	1.5085896949	5.3184429400	2.9404564963
C3	1.6674680098	4.4931777431	4.2262723263
C4	1.3888138314	3.0040198513	3.9663185801
C5	2.3207444428	2.4700890750	2.8690503007
C6	2.1935437555	3.3109747850	1.5791559833
H7	3.4807758788	4.9562965023	2.1051280379
H8	2.2569387782	5.3875094381	0.9068093068
H9	0.4642580674	5.2608642795	2.6023075408
H10	1.7173692395	6.3770127516	3.1332466318
H11	2.6902407327	4.6126986904	4.6102539486
H12	0.9936458604	4.8714171456	5.0037420831
H13	1.5262427802	2.4174831194	4.8814161843
H14	0.3415195932	2.8863163782	3.6596345689
H15	3.3529408206	2.6221989470	3.2245052863
H16	1.1777568253	3.1523962671	1.1867293723
N17	3.1057337073	2.6442224280	0.6291595169
N18	2.1934855725	1.0443483026	2.5265762088
Mn19	3.0683221382	0.6420867440	0.9037199572
C21	3.7453779942	3.2871838809	-0.2968964256
H22	3.6290968017	4.3722403100	-0.3554719733
H24	0.5553553869	0.5947597302	3.7624032014
C25	0.6011666166	-3.8519562988	2.0953343956
C26	1.6784555444	-3.1629964499	1.5539612749
C27	1.8256519582	-1.7708988526	1.7380641316
C28	0.8591210300	-1.0744570077	2.5053546663
C29	-0.2150253669	-1.7968293829	3.0405879998
C30	-0.3641026829	-3.1672395101	2.8425546377

H31	0.5130726962	-4.9239483220	1.9357210648
H33	-0.9595054392	-1.2585495121	3.6255392114
C35	6.3104164072	1.7000133865	-3.2643688535
C36	5.7475779932	0.8316709810	-2.3481937537
C37	4.8744117817	1.2999708298	-1.3327057043
C38	4.6079765606	2.7083484136	-1.2853312344
C39	5.2070343738	3.5689461365	-2.2364536971
C40	6.0456171323	3.0839951981	-3.2193358600
H41	6.9688529847	1.3062006183	-4.0350551331
H43	4.9907348793	4.6344854129	-2.1827224057
O45	4.3596577026	0.4431869902	-0.4944665624
O46	2.8471577286	-1.1769507065	1.1152264819
H44	2.4373089235	-3.6680233743	0.9638045323
H45	5.9465347982	-0.2347097131	-2.3776250901
H46	6.4940638395	3.7543108017	-3.9455814425
H47	-1.2113370516	-3.6946882443	3.2705849734
H97	1.0964249707	-1.2494207292	-0.6455651507
O99	1.0548808057	0.7434883068	-0.3317606260
C98	-3.7070413181	-0.7865343785	-0.9597662411
C99	-3.1549146057	0.4995596693	-0.9117137263
C100	-1.7809631056	0.6578198496	-0.7814095305
C101	-0.9535569126	-0.4742977157	-0.7124382142
C102	-1.5090106386	-1.7611812453	-0.7546010396
C103	-2.8879664247	-1.9149521192	-0.8747471682
H104	-4.7822686559	-0.9069628373	-1.0611526870
H105	-3.8011533692	1.3701953961	-0.9761279958
H106	-1.3221821704	1.6406115437	-0.7373143374
H107	-0.8590602056	-2.6276891019	-0.6631773351
H108	-3.3253127700	-2.9084350708	-0.8996741429

12

H99	-0.3520191750	-0.4471206793	2.0387213552
C22	0.4181511063	-0.2511130501	2.8127265965
H1	4.5620462708	-3.3388653406	-0.4647547999

C2	4.4005860014	-2.2830849943	-0.2226433135
C3	1.4794621188	-2.9806373174	3.7970948585
C4	1.4863971225	-4.5149715481	3.6988514209
C5	2.7503038150	-5.0318875750	2.9961409823
C6	2.9332415370	-4.3768431468	1.6156515948
C7	2.9219001321	-2.8499255627	1.7512772264
C8	1.6331519523	-2.3335457671	2.4036826015
H9	2.3065695450	-2.6306566107	4.4289342953
H10	0.5534414729	-2.6442036031	4.2721820189
H11	0.6004099732	-4.8451506594	3.1382518782
H12	1.4036373790	-4.9544044968	4.6993506085
H13	3.6284732263	-4.8049139915	3.6165335001
H14	2.7151683994	-6.1217620496	2.8891199000
H15	3.8681591548	-4.7175464649	1.1582836874
H16	2.1158518922	-4.6828505537	0.9465688912
H17	3.7621036841	-2.5330844710	2.3843353865
H18	0.7816972444	-2.6867326543	1.7806416394
N19	1.6866308710	-0.8736506311	2.4115100942
N20	3.0841441780	-2.1010622131	0.4717058332
Mn21	2.6841297761	-0.0831667060	1.0077065804
H23	0.0446373936	-0.7194355545	3.7318173857
H24	5.1588699622	-1.9724047955	0.5058685297
C25	4.6531755803	0.2604014004	-3.6727753986
C26	4.4297766134	0.8045540126	-2.4106128232
C27	4.3328134694	-0.0333611129	-1.2857824331
C28	4.4751111611	-1.4326075739	-1.4575896851
C29	4.6923281188	-1.9605064806	-2.7314883075
C30	4.7829373186	-1.1223323656	-3.8437308036
H31	4.7209371296	0.9197384177	-4.5342872458
H32	4.7985426851	-3.0372846699	-2.8513425827
C33	0.6066016545	4.0191116156	3.4143903096
C34	1.2366717434	3.4394466584	2.3158671993
C35	1.1977984209	2.0485804468	2.1294775828
C36	0.5166142501	1.2340032967	3.0584858977

C37	-0.0952669882	1.8357353819	4.1600903784
C38	-0.0596373355	3.2190184466	4.3457758071
H39	0.6437202250	5.0971031573	3.5478409437
H40	-0.6147471603	1.2085785774	4.8822921354
O41	1.8114816694	1.5213000337	1.0543689286
O42	4.1149728383	0.4737581771	-0.0690996159
H43	4.3207108104	1.8738946953	-2.2601521935
H44	1.7686106452	4.0373696402	1.5823236539
H45	-0.5429809138	3.6656873178	5.2097962015
H46	4.9515900108	-1.5409126851	-4.8313349373
H62	2.3476560320	-2.4006595168	-0.1745476557

13

C49	0.0520699783	-0.3365737082	-0.7082500398
H50	-0.4574176154	-0.9315782258	0.0738460981
Mn23	0.6008646478	0.0328683062	2.7010709940
O52	1.4465666468	-0.5919729196	-0.7389202550
H53	1.8147053350	-0.2279002910	0.1007693598
N21	2.3540009572	-0.0866026321	1.9218090751
H1	3.7492476469	0.8644355490	3.1899848121
C2	3.3083153191	1.0036386321	2.1833144761
H3	-0.3508316933	-3.8897714852	2.7853121223
C4	-0.5073400095	-2.8075803883	2.7249179947
C5	4.0892587753	-1.7064899818	1.0236317747
C6	4.6384354979	-3.1319003623	1.1943180239
C7	3.5215432499	-4.1801594001	1.0939444657
C8	2.4001493171	-3.8936738643	2.1065210326
C9	1.8687023292	-2.4683528685	1.9197805665
C10	2.9767440861	-1.4151896783	2.0542610387
H11	3.6701808241	-1.5805674091	0.0178735749
H12	4.9029194177	-0.9834051021	1.1318144858
H13	5.1306057131	-3.2184417639	2.1737600229
H14	5.4086806677	-3.3251010394	0.4391299050
H15	3.0990611058	-4.1637969227	0.0801848689

H16	3.9203554106	-5.1878921199	1.2556175106
H17	1.5918045807	-4.6237737949	1.9917212615
H18	2.7885571632	-4.0001658870	3.1306551285
H19	1.4369881472	-2.3572864451	0.9184408490
H20	3.4230237780	-1.5197440446	3.0659189862
N22	0.7948019819	-2.0820393027	2.8809109079
H24	4.1420026967	0.9428847494	1.4727184748
H25	-0.8612376577	-2.5644611298	1.7164878746
C26	-3.3232797558	-1.3858597921	5.6377684339
C27	-2.7484100827	-0.5043695751	4.7261237543
C28	-1.8213271030	-0.9731290374	3.7798966244
C29	-1.4900471474	-2.3503929762	3.7635059716
C30	-2.0709771286	-3.2172724378	4.6902650593
C31	-2.9887458280	-2.7442440621	5.6294707527
H32	-4.0351784150	-1.0092675369	6.3681093966
H33	-1.8058797065	-4.2729341839	4.6717136857
C34	1.7235905116	5.0132621050	2.1123660533
C35	1.0173758479	3.9985373010	2.7518046922
C36	1.5067179928	2.6847270077	2.7418254396
C37	2.7228019621	2.3909349138	2.0894069611
C38	3.4030046353	3.4210770232	1.4369553735
C39	2.9158972246	4.7290841591	1.4428679486
H40	1.3328950167	6.0276505028	2.1233099460
H41	4.3352806484	3.1950953297	0.9229541103
O42	0.8004217819	1.7220540851	3.3661919399
O43	-1.2602842530	-0.1363396384	2.9002466431
H44	-2.9899781448	0.5539799879	4.7239327958
H45	0.0765662950	4.1895391529	3.2576706838
H46	3.4607142365	5.5165660369	0.9298900800
H47	-3.4359738734	-3.4245100777	6.3479925870
H48	1.1329392286	-2.2629813326	3.8317098035
H51	-0.3462214581	-0.6895972096	-1.6676958596
C54	-0.9164736553	3.8394684828	-0.1658013438
C55	0.2316250758	3.4680142587	-0.8695457705

C56	0.5438757879	2.1209539881	-1.0365661151
C57	-0.2879277298	1.1285083809	-0.5023240361
C58	-1.4278543159	1.5066880641	0.2157041955
C59	-1.7424852874	2.8575317685	0.3793497675
H60	-1.1504580447	4.8911090879	-0.0231038622
H61	0.8945651644	4.2307944054	-1.2677001188
H62	1.4436265030	1.8216153732	-1.5650734132
H63	-2.0454132429	0.7471292730	0.6856999757
H64	-2.6216660463	3.1391792468	0.9523305343

14ts

O52	0.0000000000	0.0000000000	0.0000000000
H53	0.0000000000	0.0000000000	1.2286562378
N21	0.7788609926	0.0000000000	2.2595514666
C49	-0.9291524703	-0.8054802604	-0.7095062116
H50	-0.9305143259	-1.8356232389	-0.3282873025
Mn23	2.0089981357	-0.4123121570	0.6813829989
H1	1.5198582711	-1.2759268842	3.7689951407
C2	0.5776166747	-1.0935063764	3.2276801383
H3	2.0677337067	3.2687760704	-0.7115451823
C4	2.1554677427	2.1762564010	-0.7008434137
C5	-0.0895242646	1.8786344738	3.7169868678
C6	0.2122074561	3.3134990241	4.1804138464
C7	0.4659919383	4.2523804307	2.9917392643
C8	1.5797753118	3.7058233638	2.0828413792
C9	1.2427465528	2.2818284793	1.6269472413
C10	1.0384165437	1.3333185139	2.8191153091
H11	-1.0266969196	1.8510851656	3.1441140773
H12	-0.2278764560	1.2300406105	4.5873044357
H13	1.0997650448	3.3035683248	4.8288656630
H14	-0.6163631993	3.6891739468	4.7912276373
H15	-0.4568301460	4.3540320294	2.4042281589
H16	0.7288333258	5.2563278632	3.3432579692
H17	1.7240849830	4.3613931656	1.2169428186

H18	2.5314081063	3.6886367146	2.6348251117
H19	0.3112524782	2.2821964954	1.0497676755
H20	1.9725407782	1.3092083877	3.4111967818
N22	2.2653952270	1.6880532364	0.7125275111
H24	-0.1803636246	-0.8151980711	3.9693116153
H25	1.2165942151	1.7511673103	-1.0723880282
C26	5.4561452233	0.9313878670	-3.1635037909
C27	4.8095684365	0.0018031531	-2.3566613101
C28	3.7412656357	0.3879370188	-1.5253003877
C29	3.3348594628	1.7464276025	-1.5286048852
C30	4.0097318934	2.6662290959	-2.3367685278
C31	5.0642923639	2.2734876176	-3.1584639065
H32	6.2756904647	0.6072666454	-3.7998899381
H33	3.6913921148	3.7072881121	-2.3266623701
C34	-0.6496446016	-4.6252502695	1.0849529570
C35	0.5461866653	-3.9863853102	0.7647950499
C36	0.9427754550	-2.8368062763	1.4674425481
C37	0.1269600311	-2.3408713349	2.5099670792
C38	-1.0743462349	-2.9861527661	2.8043109142
C39	-1.4699731413	-4.1271525642	2.1013868178
H40	-0.9514822717	-5.5079401098	0.5269121933
H41	-1.7109655967	-2.5872890814	3.5914060147
O42	2.0813670198	-2.2054592902	1.1307486826
O43	3.1541116861	-0.5449664346	-0.7717854914
H44	5.1019681083	-1.0434638834	-2.3468112671
H45	1.1868083618	-4.3422779358	-0.0360519020
H46	-2.4090496345	-4.6179236300	2.3399761551
H47	5.5726577981	3.0011762874	-3.7836444521
H48	3.2006322324	1.9231886669	1.0548826254
H51	-0.6137366654	-0.8450505642	-1.7618362531
C54	-4.8357760690	0.9987394907	-0.3373889725
C55	-4.5734554776	-0.2239810478	0.2805660994
C56	-3.3161530976	-0.8200955752	0.1511697611
C57	-2.3125123908	-0.2043030577	-0.6019447301

C58	-2.5842193052	1.0265658935	-1.2152813543
C59	-3.8353539691	1.6254714566	-1.0867580586
H60	-5.8137979313	1.4625260597	-0.2389337369
H61	-5.3476922588	-0.7142998692	0.8651202949
H62	-3.1057354059	-1.7680240009	0.6411427558
H63	-1.8024106027	1.5120125606	-1.7954612640
H64	-4.0355628702	2.5773668379	-1.5726132072

15

Mn1	0.0752823677	-0.1729115864	2.3021664605
H2	2.1140970589	-0.5369654061	0.7137138136
N3	2.0458121918	-0.8168194656	1.6923636884
H4	3.0813896818	-0.5262921398	3.4686329306
C5	3.1433926633	-0.1319950045	2.4480858708
H6	-1.0175712153	-3.5430909063	4.3931040440
C7	-1.0299491173	-2.5622804859	3.8996693425
C8	2.6124989858	-2.9475017904	0.4597891348
C9	2.8248263116	-4.4615548122	0.6363814993
C10	1.5798894335	-5.1510946032	1.2208225118
C11	1.1048100994	-4.4707529617	2.5178163769
C12	0.8455869469	-2.9799706458	2.2557844541
C13	2.1542350890	-2.3137322094	1.7770514458
H14	1.8429956458	-2.7557019433	-0.3006437605
H15	3.5389731073	-2.4686705321	0.1178229862
H16	3.6774244114	-4.6219091824	1.3111553469
H17	3.0956444016	-4.9146531530	-0.3237530377
H18	0.7656122086	-5.1094918064	0.4844488456
H19	1.7866527088	-6.2118343122	1.4011672867
H20	0.1898798586	-4.9496591406	2.8824741356
H21	1.8627710181	-4.5824872791	3.3076671260
H22	0.1093122931	-2.8840128528	1.4501660883
H23	2.9168322027	-2.5174494478	2.5416375566
N24	0.3082450460	-2.1894969805	3.3748113467
H25	4.1198067987	-0.4106768520	2.0296411387

H26	-1.6917595094	-2.6176312264	3.0275975443
C27	-2.3186238694	0.5836766781	6.5433855175
C28	-2.1697770523	0.8277165708	5.1805978019
C29	-1.7695351443	-0.2029705180	4.3164506835
C30	-1.5117862385	-1.4927071098	4.8480789064
C31	-1.6610123848	-1.7147709828	6.2172287997
C32	-2.0660951340	-0.6858695706	7.0705855755
H33	-2.6231319873	1.3928331212	7.2027783853
H34	-1.4569562624	-2.7053531305	6.6201118242
C35	2.5929433517	4.1043500822	2.7770264074
C36	1.6241347900	3.2279718645	3.2565975705
C37	1.7912023650	1.8396976568	3.1119299815
C38	2.9673194806	1.3557323357	2.4859369181
C39	3.9216115978	2.2496250848	1.9986768271
C40	3.7417975650	3.6261891772	2.1380251010
H41	2.4461135613	5.1757603205	2.8912156156
H42	4.8183792062	1.8626049933	1.5176912474
O43	0.8702499380	0.9840121974	3.5700331558
O44	-1.6431438295	0.0166435271	2.9991411913
H45	-2.3433235378	1.8107179389	4.7544492422
H46	0.7202777908	3.5848127624	3.7394884912
H47	4.4881685519	4.3176612789	1.7580688681
H48	-2.1757880167	-0.8700423355	8.1353532157
H49	0.9707358936	-2.1282121208	4.1475739949
O50	-0.5165042620	-0.8384557023	0.6635177152
C51	-0.2648551143	-0.0964005668	-0.5004130679
H52	0.6773963161	-0.4150990816	-0.9931788311
H53	-1.0676416920	-0.3003511937	-1.2276894188
C54	-0.0736578995	4.1561338116	0.2906511760
C55	-1.1431114299	3.3936377013	0.7646175274
C56	-1.2017005351	2.0259359547	0.4998172751
C57	-0.1865512720	1.4022800145	-0.2433066915
C58	0.8893371613	2.1726025859	-0.6979507894
C59	0.9455097060	3.5427619019	-0.4380039778

H60	-0.0237126750	5.2201660404	0.5065394275
H61	-1.9297439731	3.8626655310	1.3497633406
H62	-2.0149522725	1.4248380304	0.8951929968
H63	1.6922116720	1.6962776544	-1.2574157339
H64	1.7947574292	4.1256136404	-0.7828317775

16ts

C49	0.0000000000	0.0000000000	0.0000000000
H50	0.0000000000	0.0000000000	1.4946443426
Mn23	0.9578813927	0.0000000000	2.8949990170
O52	0.4687314220	-1.1046926812	-0.4077041735
H53	1.5562489746	-1.4800330796	0.9032140947
N21	1.7773929162	-1.6650052416	1.9091953798
H1	3.4096938659	-2.1805355288	3.1003302796
C2	3.2570553321	-1.7819387074	2.0905585638
H3	-2.5649132351	-1.5785663628	4.1025624003
C4	-1.7706403744	-0.8676465814	3.8440326573
C5	1.1491099652	-3.9861868790	1.1792471842
C6	0.3725940195	-5.2575459059	1.5504717728
C7	-1.0960217174	-4.9321590622	1.8564384410
C8	-1.2009595737	-3.8721023796	2.9631059441
C9	-0.4266471189	-2.5958759645	2.5977783049
C10	1.0465140531	-2.9166623463	2.2803243479
H11	0.7438802466	-3.5492348354	0.2581753764
H12	2.2018646002	-4.2239860179	0.9919637262
H13	0.8329931754	-5.7261252608	2.4322649119
H14	0.4426904886	-5.9859740665	0.7351315123
H15	-1.5807132373	-4.5523297009	0.9468144299
H16	-1.6398492759	-5.8362758621	2.1520864693
H17	-2.2498744597	-3.6254983725	3.1590981938
H18	-0.7882429709	-4.2764160889	3.9000786454
H19	-0.8602141153	-2.1424606864	1.6985626954
H20	1.5124392982	-3.2882086949	3.2048924321
N22	-0.4625115386	-1.5561804874	3.6559592985

H24	3.6724306192	-2.4955723850	1.3707312191
H25	-2.0138100340	-0.4209736923	2.8724604530
C26	-1.4401505015	2.2178665486	6.8309515746
C27	-0.6751324625	2.2891749322	5.6699913722
C28	-0.7740709966	1.2889658165	4.6877390359
C29	-1.6601633961	0.2004233004	4.9013321619
C30	-2.4093914685	0.1451362213	6.0782010105
C31	-2.3114900723	1.1465575682	7.0450710939
H32	-1.3513637953	3.0023370981	7.5782614961
H33	-3.0866530644	-0.6927709708	6.2331718765
C34	5.1786628914	2.0544080348	1.8989741647
C35	4.1885125028	1.8091399717	2.8467927391
C36	3.5553910154	0.5554884869	2.8970799674
C37	3.9383745647	-0.4480495793	1.9720386914
C38	4.9251967379	-0.1797546806	1.0241822242
C39	5.5520793754	1.0668157547	0.9825020698
H40	5.6561575193	3.0304326506	1.8664094421
H41	5.2040689913	-0.9543125536	0.3125432875
O42	2.5834298092	0.3033774468	3.7942258066
O43	-0.0597141369	1.3980244171	3.5600103268
H44	0.0107983374	3.1110848251	5.4895047239
H45	3.8774896492	2.5680996920	3.5575941152
H46	6.3177059150	1.2688829882	0.2392238400
H47	-2.9050992841	1.0912406809	7.9525559860
H48	-0.1742370043	-1.9590638350	4.5490495379
H51	-1.0999684759	0.1463853381	0.0294137556
C54	2.2294195429	3.6541619240	-0.4118323163
C55	2.7699677174	2.4485421102	-0.8624672766
C56	2.0414620646	1.2662602402	-0.7388161372
C57	0.7681818618	1.2870745888	-0.1631212287
C58	0.2281335498	2.4980181156	0.2908232238
C59	0.9560999710	3.6777127923	0.1659526288
H60	2.7996416009	4.5745705851	-0.5055441708
H61	3.7639187034	2.4272596482	-1.2990405687

H62	2.4419870122	0.3166541006	-1.0783928209
H63	-0.7463464389	2.5014849569	0.7737341908
H64	0.5380039649	4.6139925989	0.5256440417

17

H50	0.1061729051	-0.2968307246	0.9148164027
Mn23	0.5964433876	-0.0825914823	2.4456590079
H53	2.4143215451	-0.8817393861	0.7727407439
N21	2.3522933668	-1.1159229996	1.7633023442
H1	3.5564419379	-1.0147042893	3.4565421682
C2	3.5979350114	-0.6117362357	2.4393818367
H3	-1.5038995393	-3.2185795268	4.0006032663
C4	-1.2274614166	-2.2296691864	3.6126844839
C5	2.7251434245	-3.3603811426	0.6785278908
C6	2.6005486254	-4.8797582800	0.8736093861
C7	1.1542611014	-5.2843825314	1.1977191085
C8	0.6129814617	-4.5074607386	2.4089887685
C9	0.7141493542	-2.9902167846	2.1807697964
C10	2.1810520271	-2.6004865555	1.8945360954
H11	2.1540944992	-3.0530898692	-0.2095729402
H12	3.7700023582	-3.0762330283	0.5046390183
H13	3.2583968137	-5.1899671952	1.6974681794
H14	2.9536944566	-5.4011811821	-0.0228169615
H15	0.5165526630	-5.0784995617	0.3268591614
H16	1.0930134642	-6.3623455679	1.3832953446
H17	-0.4282562905	-4.7835902433	2.6045952921
H18	1.1876229818	-4.7702599236	3.3097956295
H19	0.1105974799	-2.7167922978	1.3049458852
H20	2.7633084742	-2.8925640397	2.7789078062
N22	0.2220915328	-2.1502878978	3.2879261879
H24	4.4872293910	-1.0137274254	1.9377661466
H25	-1.7580642636	-2.0673085759	2.6671220638
C26	-2.2136880406	0.9036127628	6.4018047192
C27	-1.7855282752	1.2209061020	5.1151720921

C28	-1.4696666137	0.2047477582	4.1983533729
C29	-1.5899482373	-1.1504266035	4.6026001314
C30	-2.0118694975	-1.4462319627	5.9001798887
C31	-2.3282176374	-0.4305780938	6.8041202584
H32	-2.4505726135	1.7027669448	7.0997052244
H33	-2.0966530890	-2.4881984473	6.2047316694
C34	3.6166005579	3.6571641977	2.8311389111
C35	2.6162600509	2.8979612764	3.4306824761
C36	2.6004159530	1.4971509370	3.2782934346
C37	3.6276523745	0.8854764391	2.5089129484
C38	4.6177971298	1.6654663704	1.9088625619
C39	4.6195115952	3.0516482142	2.0640088223
H40	3.6140180118	4.7371922356	2.9570955241
H41	5.3976007746	1.1810284777	1.3235810792
O42	1.6440799598	0.7419249170	3.8222605188
O43	-1.0845220856	0.5166971372	2.9498606831
H44	-1.6782053679	2.2503003508	4.7880401545
H45	1.8303169714	3.3546284207	4.0242528399
H46	5.3913175127	3.6545674926	1.5950662531
H47	-2.6546114698	-0.6763095943	7.8104857096
H48	0.7659399755	-2.3068038958	4.1367517573

18ts

N21	0.0000000000	0.0000000000	0.0000000000
H53	0.0000000000	0.0000000000	1.2863403322
H50	0.5555967963	0.0000000000	2.2197521212
Mn23	1.8911332295	0.2253666091	0.6762911881
H1	-0.1551759124	1.2952003352	-1.6497973886
C2	-0.6278396764	1.1571544180	-0.6633902195
H3	2.4341095517	-3.5339821125	1.7163181736
C4	2.6017595009	-2.4523136695	1.6684026578
C5	-1.7222315914	-1.7283586430	-0.6927554711
C6	-1.8668921210	-3.1538529093	-1.2513208844
C7	-1.0782013116	-4.1748151812	-0.4179831649

C8	0.3983312011	-3.7639272696	-0.2891356939
C9	0.5070972269	-2.3473599530	0.2855664710
C10	-0.2394017900	-1.3240183764	-0.5865612413
H11	-2.1711425273	-1.6612309143	0.3076474775
H12	-2.2618851262	-1.0241594519	-1.3327245297
H13	-1.4999383914	-3.1725375994	-2.2871905222
H14	-2.9257393988	-3.4323779618	-1.2904725437
H15	-1.5186181147	-4.2445006599	0.5861697981
H16	-1.1504409293	-5.1730556042	-0.8634306083
H17	0.9410965201	-4.4765450036	0.3413273231
H18	0.8750615400	-3.7828142035	-1.2804506393
H19	0.0585536141	-2.3209003642	1.2856468397
H20	0.1976849381	-1.3539417743	-1.6029421590
N22	1.9109307592	-1.8762988119	0.4692345110
H24	-1.6932636098	0.9665622725	-0.8350019873
H25	2.1136375343	-1.9921740423	2.5369495310
C26	6.8096893495	-1.5692565893	1.6343227252
C27	5.8863655420	-0.5472490482	1.4422588588
C28	4.5049899621	-0.8187177284	1.4361343856
C29	4.0745386576	-2.1550306237	1.6321729437
C30	5.0205613209	-3.1688356058	1.8104691523
C31	6.3857260366	-2.8894722197	1.8171158870
H32	7.8712890754	-1.3352427972	1.6374664419
H33	4.6766017856	-4.1915495083	1.9544746820
C34	-0.1491379335	4.7246062618	1.7094717299
C35	0.9559356412	3.9375817311	1.3964076375
C36	0.7978333120	2.7765565014	0.6247813313
C37	-0.4870304211	2.4071583111	0.1707911300
C38	-1.5830986508	3.2023392101	0.5071881987
C39	-1.4247661915	4.3607635490	1.2697631823
H40	-0.0157567231	5.6205255186	2.3104425993
H41	-2.5747084398	2.9093090970	0.1680733422
O42	1.8756990920	2.0272933563	0.3264936831
O43	3.6544313421	0.1920366895	1.2499995777

H44	6.1983584054	0.4821494258	1.2964145702
H45	1.9541576449	4.1907000538	1.7395182841
H46	-2.2879753582	4.9680605811	1.5256407702
H47	7.1080152330	-3.6871953696	1.9608352656
H48	2.4598224367	-2.1296122950	-0.3566810998

References

1) A. A. Tsygankov, M.S. Chun, A. D. Samoilova, S. Kwon, Y. M. Kreschenova, S. Kim, E. J. Shin, J. Oh, T. V. Strelkova, V. S. Kolesov, F. I. Zubkov, S. E. Semenov, I. V. Fedyanin and D. Chusov, *Synlett*, 2017, **28**, 615-619.
2) A. Hille and R. Gust, *Arch. Pharm. Chem. Life Sci.*, 2009, **342**, 625-631.
3) Y. N. Belokon, J. Fuentes, M. North and J. W. Steed, *Tetrahedron*, 2004, **60**, 3191-3204.
4) J. F. Larrow and E. N. Jacobsen, *Org. Synth*., 1998, **75**, 1-11.
5) A. R. Silva, C. Freire and B. de Castro, *New. J. Chem*., 2004, **28**, 253-260.
6) I. Nemec, R. Herchel, Z. Trávnícek and T. Silha, *RSC Adv*., 2016, **6**, 3074-3083.
7) D. J. Gravert and J. H. Griffin, *Inorg. Chem*., 1996, **35**, 4837-4847.
8) G. Zhang and S. K. Hanson, *Org. Lett*., 2013, **15**, 650-653.
9) B. Saha, S. M. W. Rahaman, P. Daw, G. Sengupta and J. K. Bera, *Chem. Eur. J*., 2014, **20**, 6542-6551.
10) D. Srimani and A. Sarkar, *Tetrahedron Lett*., 2008, **49**, 6304-6307.
11) K. Azizi and R. Madsen, *ChemCatChem*, 2018, **10**, 3703-3708.
12) M.-H. So, Y. Liu, C.-M. Ho and C.-M. Che, *Chem. Asian J.*, 2009, **4**, 1551-1561.
13) M. Okimoto, Y. Takahashi, K. Numata, Y. Nagata and G. Sasaki, *Synth. Commun*., 2005, **35**, 1989-1995.
14) D. L. Comins and J. D. Brown, *J. Org. Chem*., 1984, **49**, 1079-1083.
15) Y. Lan, B. Liao, Y. Liu, S. Peng and S. Liu, *Eur. J. Org. Chem*., 2013, 5160-5164.
16) J. Alender, P. Morgan and J. Timberlake, *J. Org. Chem.*, 1983, **48**, 755-758.
17) I. A. Cliffe, R. Crossley and R. G. Shepherd, *Synthesis*, 1985, 1138-1140.
18) H. Yamada, T. Kawate, A. Nishida and M. Nakagawa, *J. Org. Chem*., 1999, **64**, 8821-8828.
19) D. J. Vyas, R. Fröhlich and M. Oestreich, *Org. Lett.*, 2011, **13**, 2094-2097.
20) A. E. Wendlandt and S. S. Stahl, *Org. Lett*., 2012, **14**, 2850-2853.
21) W. Bao, H. Kossen and U. Schneider, *J. Am. Chem. Soc*., 2017, **139**, 4362-4365.
22) T. Patra, S. Agasti, Akanksha and D. Maiti, *Chem. Commun*., 2013, **49**, 69-71.
23) T. Yan and K. Barta, *ChemSusChem*, 2016, **9**, 2321-2325.

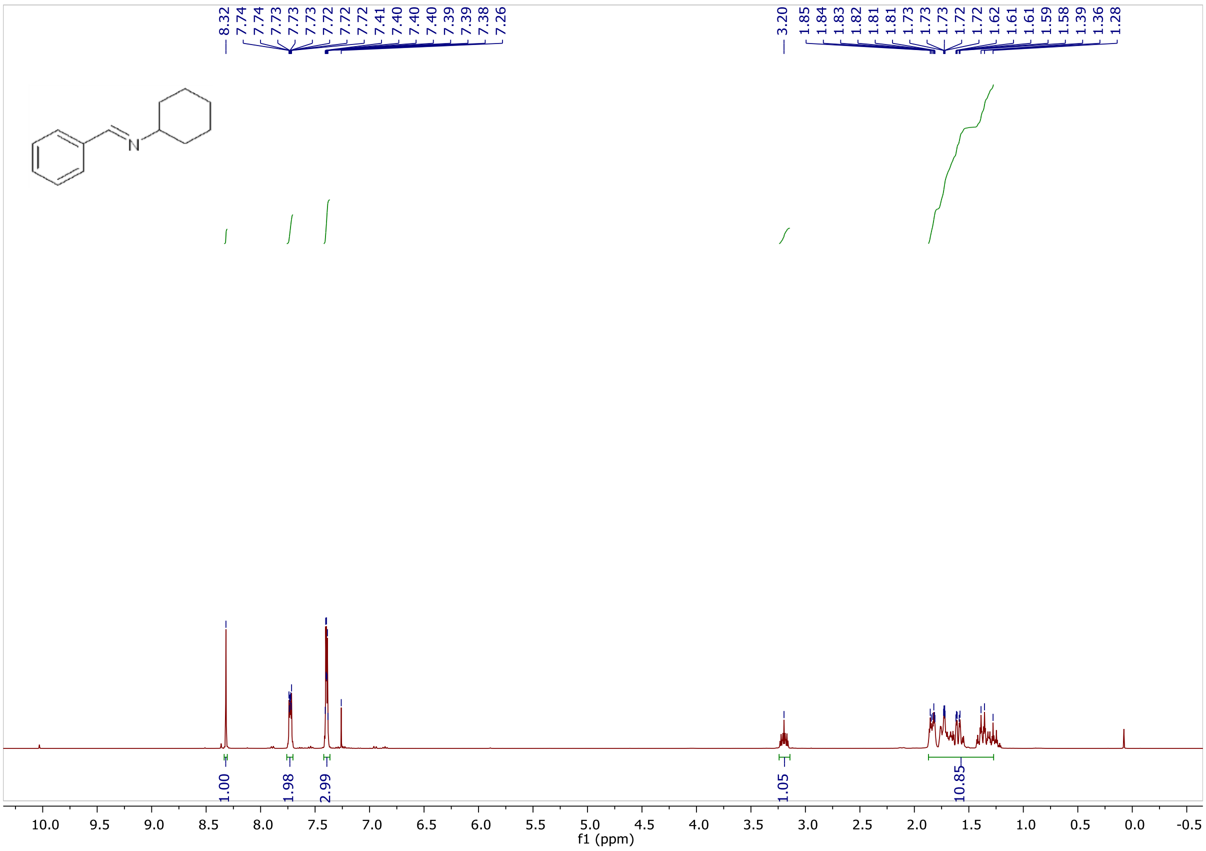

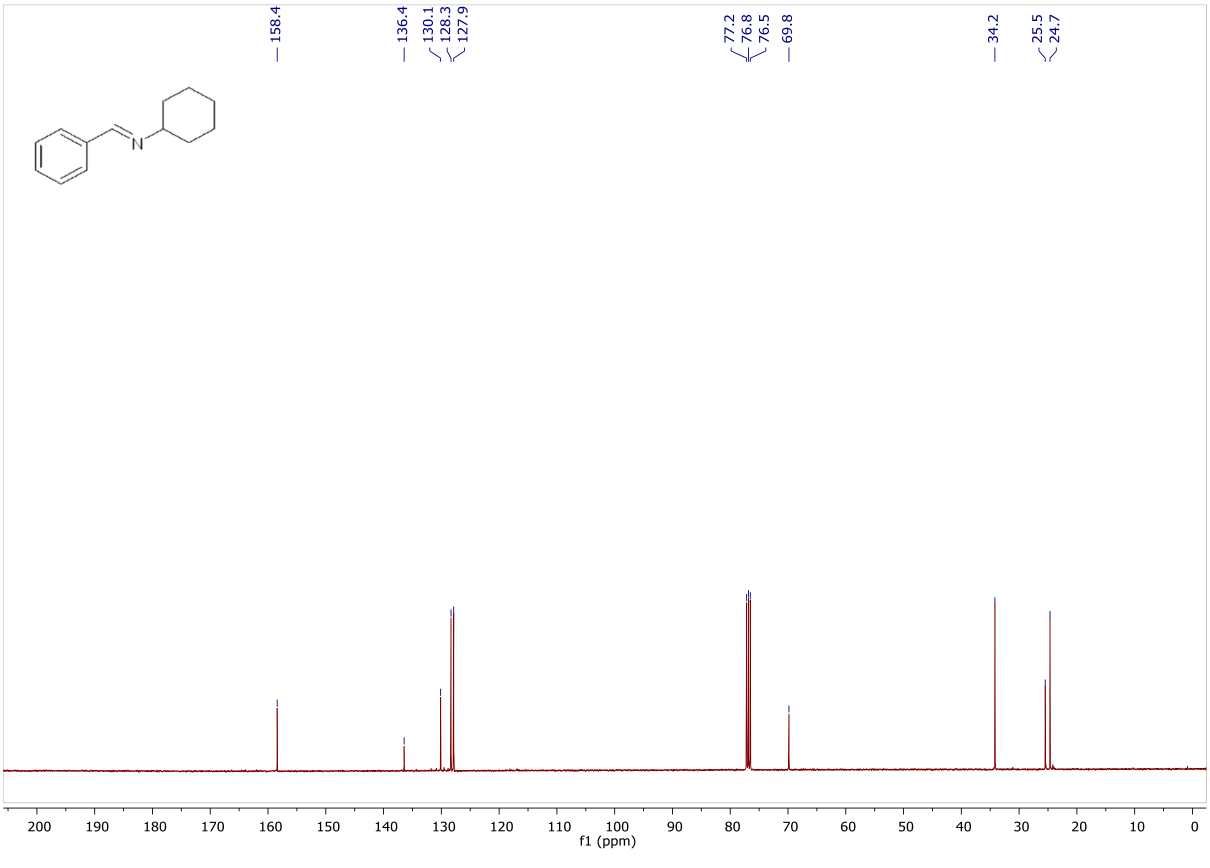

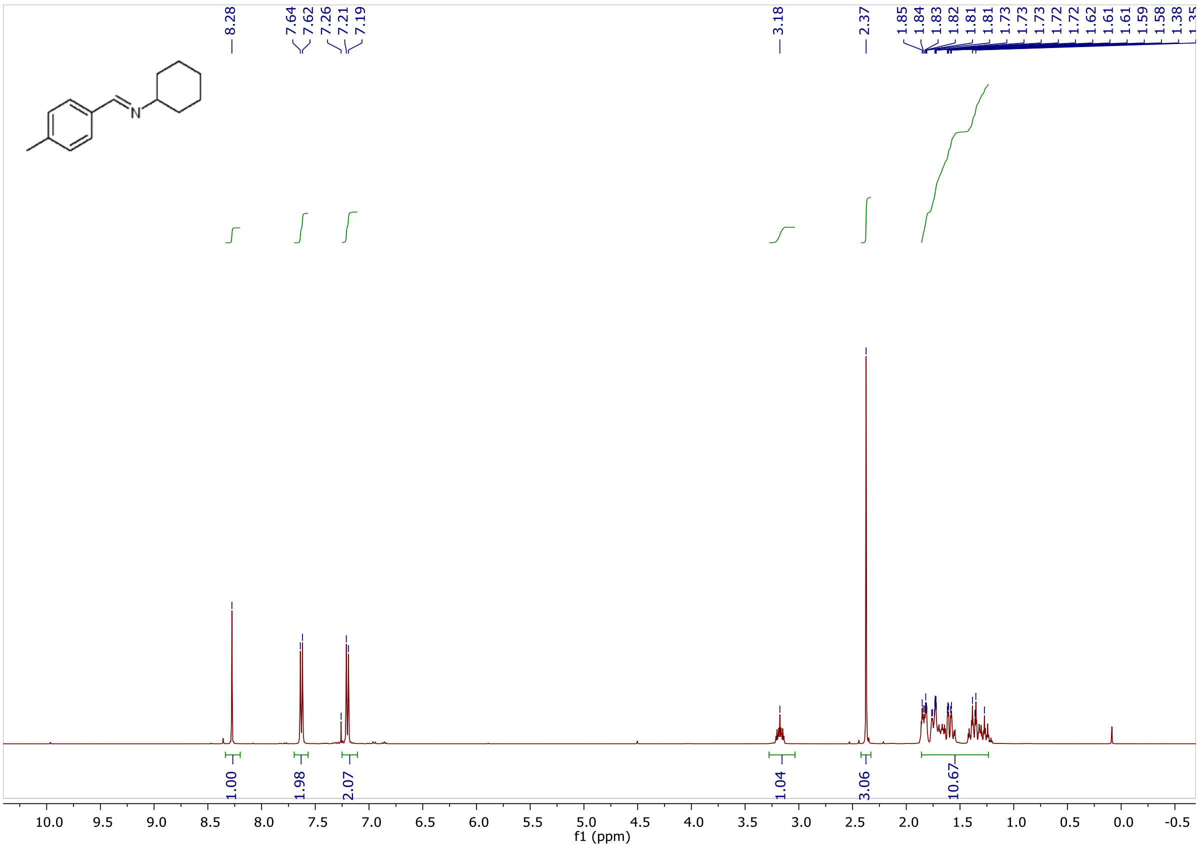
N
8.28
7.64
7.62
7.26
7.21
7.19
3.18
2.37
1.85
1.84
1.83
1.82
1.81
1.81
1.73
1.73
1.73
1.72
1.72
1.62
1.61
1.61
1.59
1.58
1.38
1.00
1.98
2.07
1.04
3.06
10.67
10.0 9.5 9.0 8.5 8.0 7.5 7.0 6.5 6.0 5.5 5.0 4.5 4.0 3.5 3.0 2.5 2.0 1.5 1.0 0.5 0.0 -0.5
f1 (ppm)

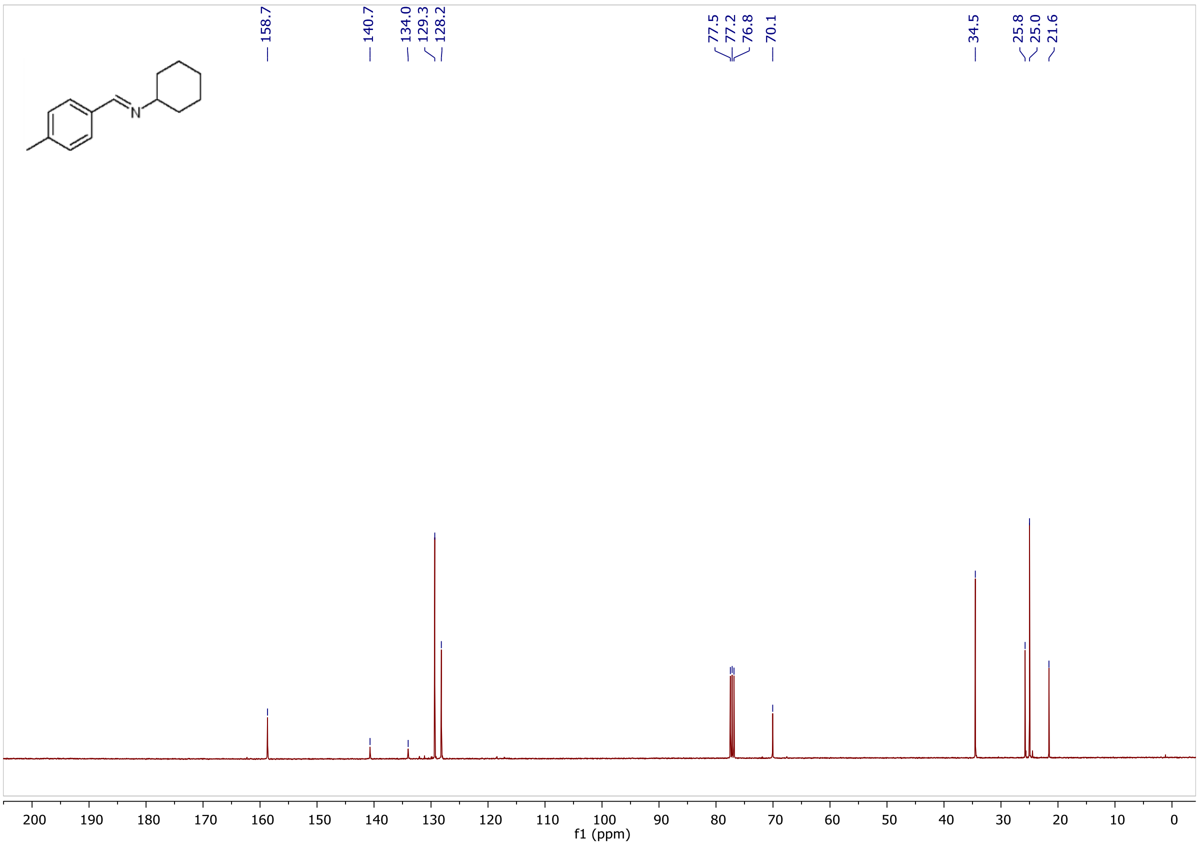
N
158.7
140.7
134.0
129.3
128.2
77.5
77.2
76.8
70.1
34.5
25.8
25.0
21.6
200 190 180 170 160 150 140 130 120 110 100 90 80 70 60 50 40 30 20 10 0
f1 (ppm)

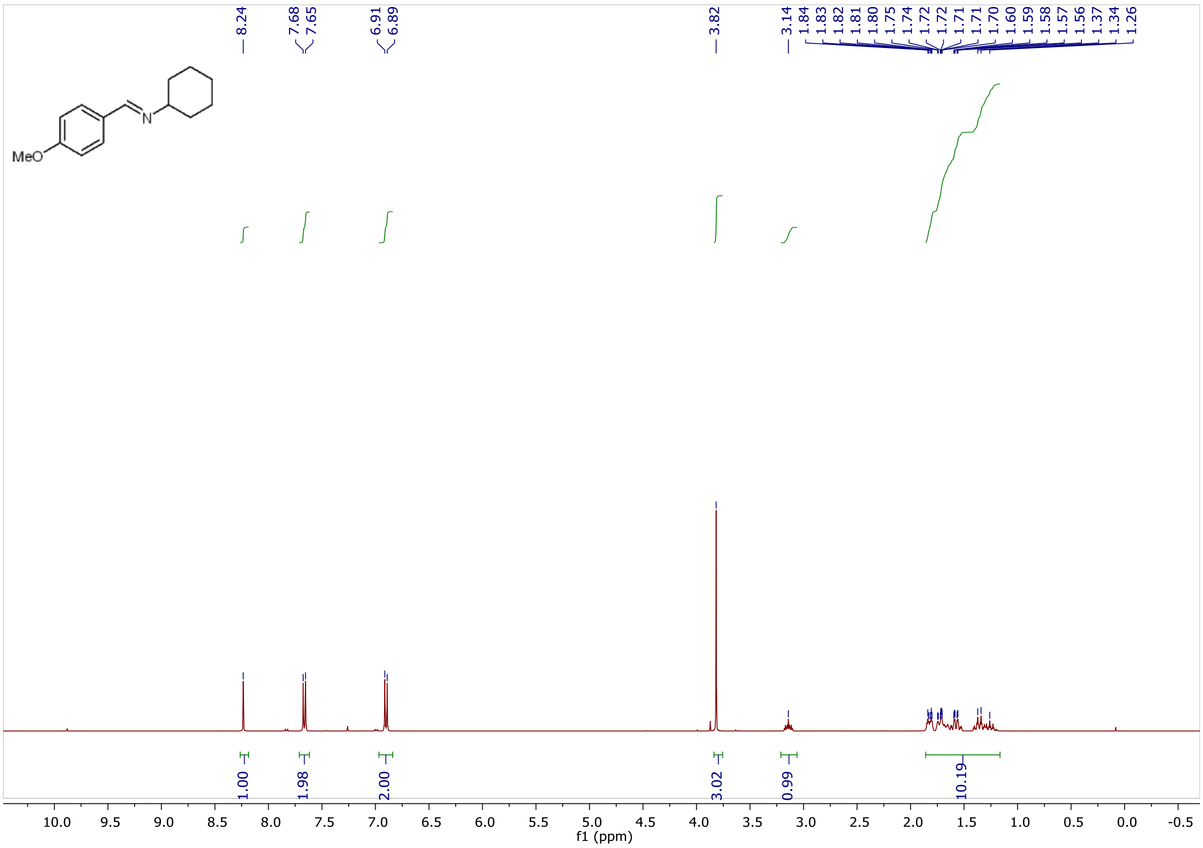

MeO
N
8.24
7.68
7.65
6.91
6.89
3.82
3.14
1.84
1.83
1.82
1.81
1.80
1.75
1.74
1.72
1.72
1.71
1.71
1.70
1.60
1.59
1.58
1.57
1.56
1.37
1.34
1.26
1.00
1.98
2.00
3.02
0.99
10.19
10.0 9.5 9.0 8.5 8.0 7.5 7.0 6.5 6.0 5.5 5.0 4.5 4.0 3.5 3.0 2.5 2.0 1.5 1.0 0.5 0.0 -0.5
f1 (ppm)

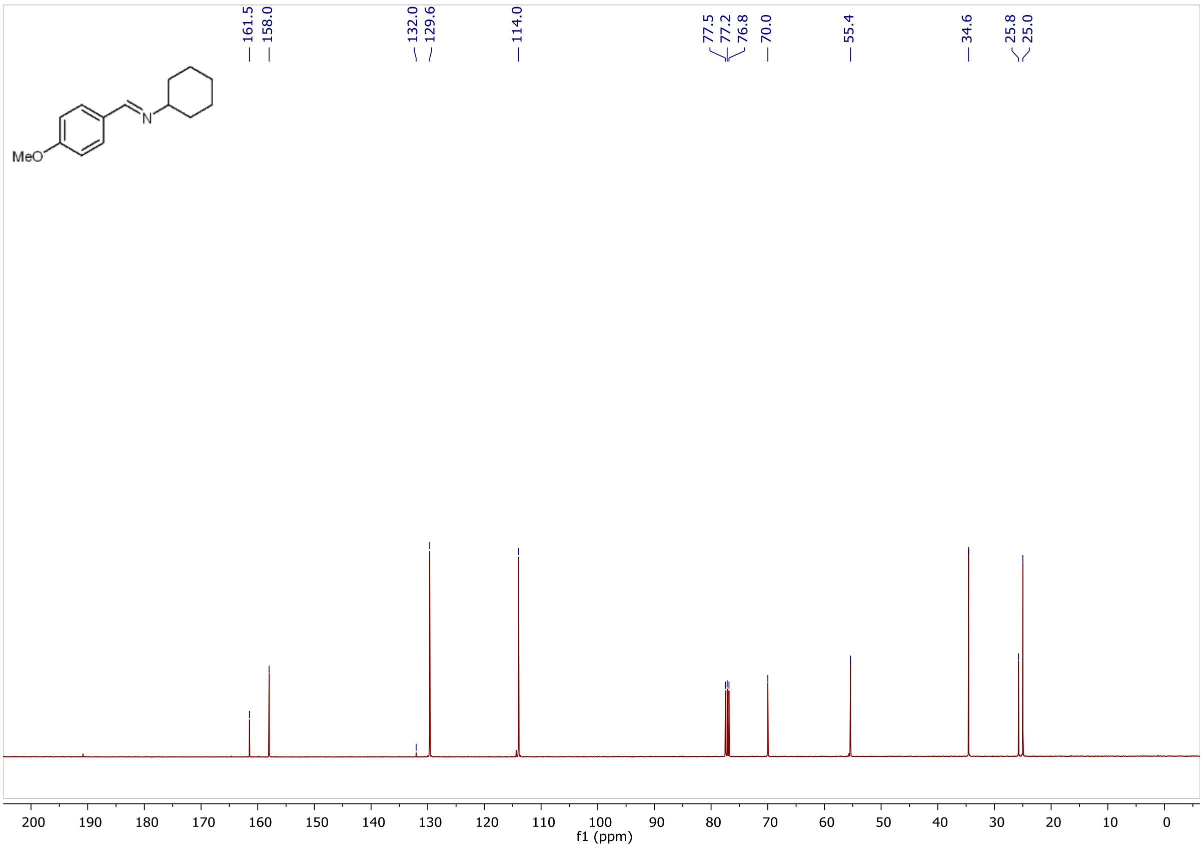

MeO
N
161.5
158.0
132.0
129.6
114.0
77.5
77.2
76.8
70.0
55.4
34.6
25.8
25.0
200 190 180 170 160 150 140 130 120 110 100 90 80 70 60 50 40 30 20 10 0
f1 (ppm)

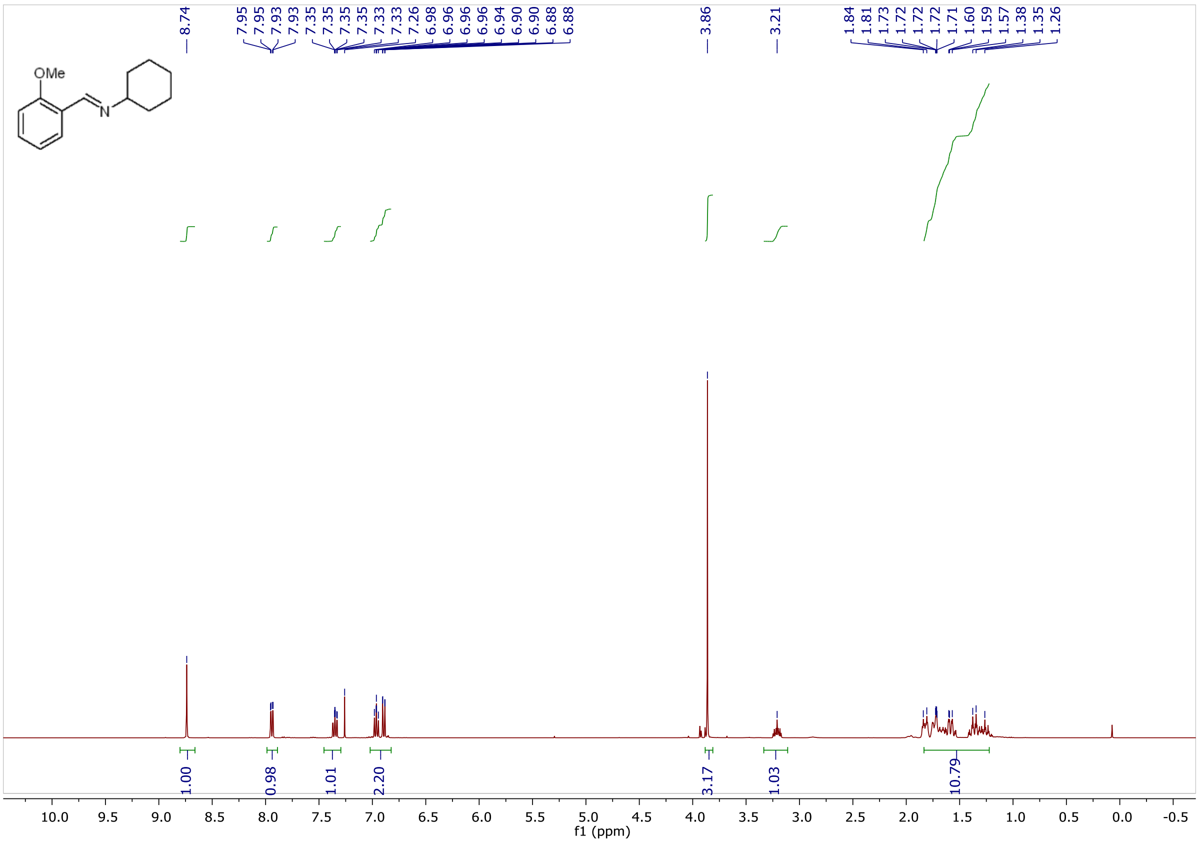

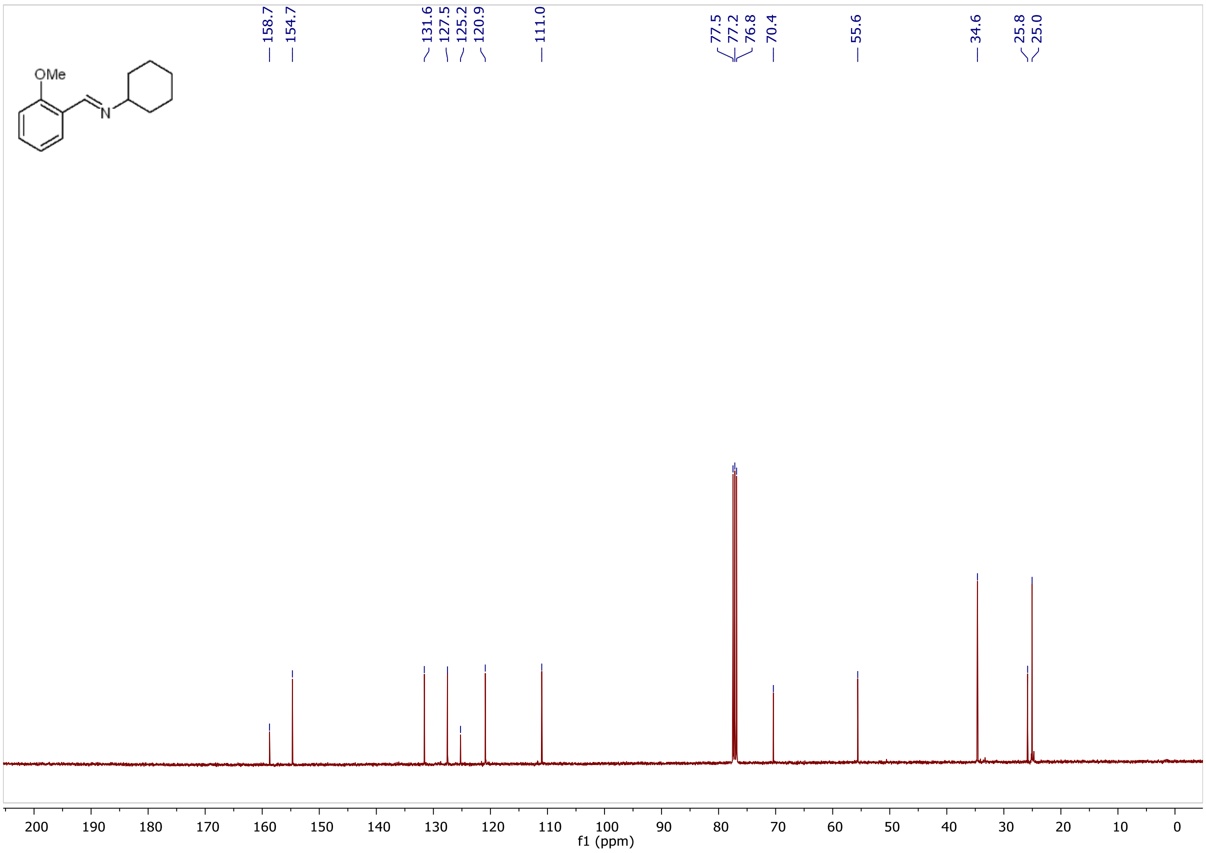

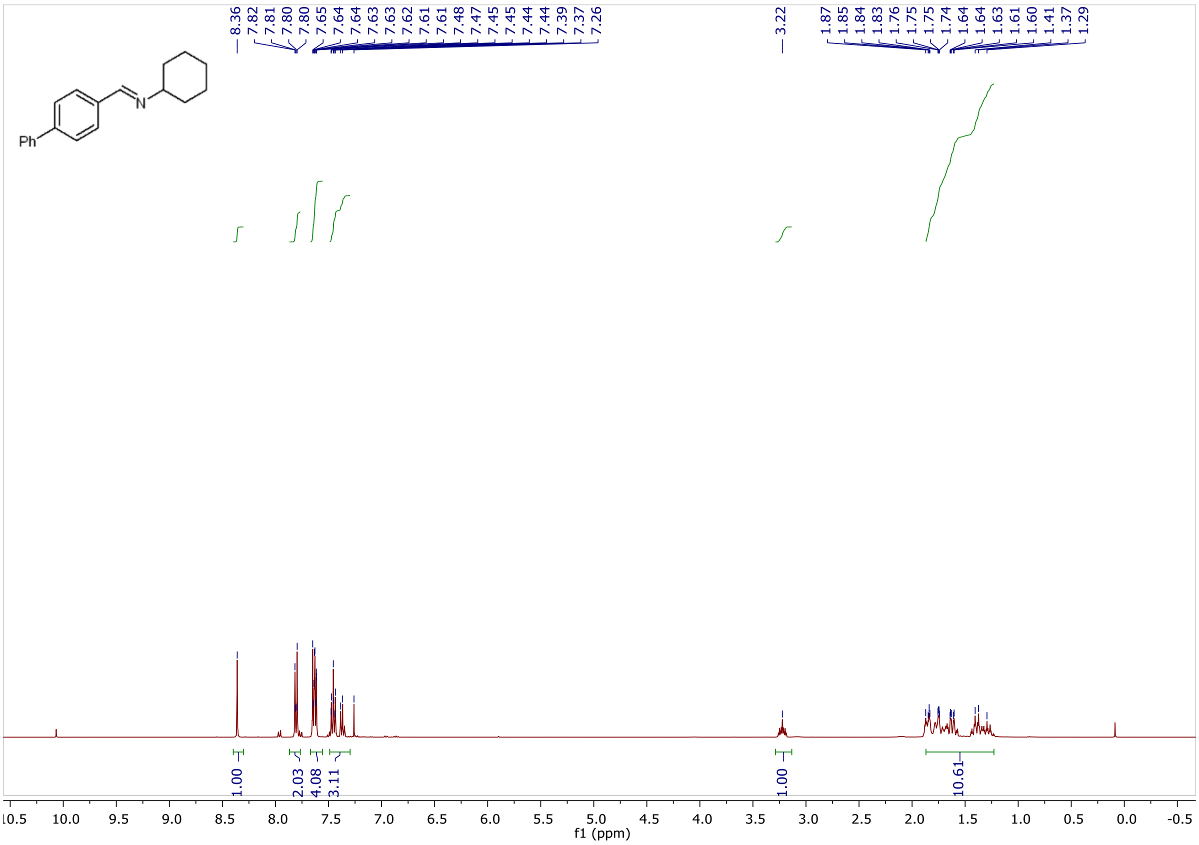
8.36
7.82
7.81
7.80
7.80
7.65
7.64
7.64
7.63
7.63
7.62
7.61
7.61
7.48
7.47
7.45
7.45
7.44
7.44
7.39
7.37
7.26
3.22
1.87
1.85
1.84
1.83
1.76
1.75
1.75
1.74
1.64
1.64
1.63
1.61
1.60
1.41
1.37
1.29
Ph
N
1.00
2.03
4.08
3.11
1.00
10.61
f1 (ppm)

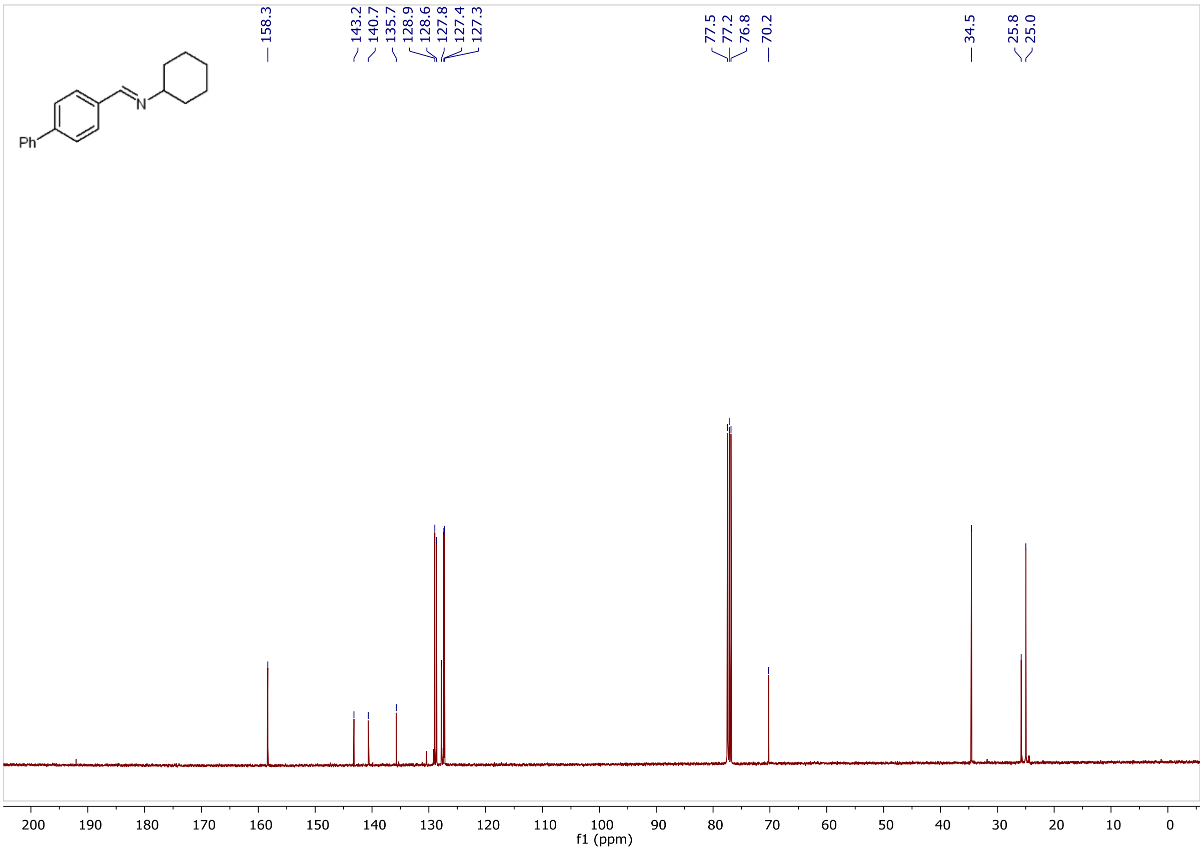
158.3
143.2
140.7
135.7
128.9
128.6
127.8
127.4
127.3
77.5
77.2
76.8
70.2
34.5
25.8
25.0
Ph
N
f1 (ppm)

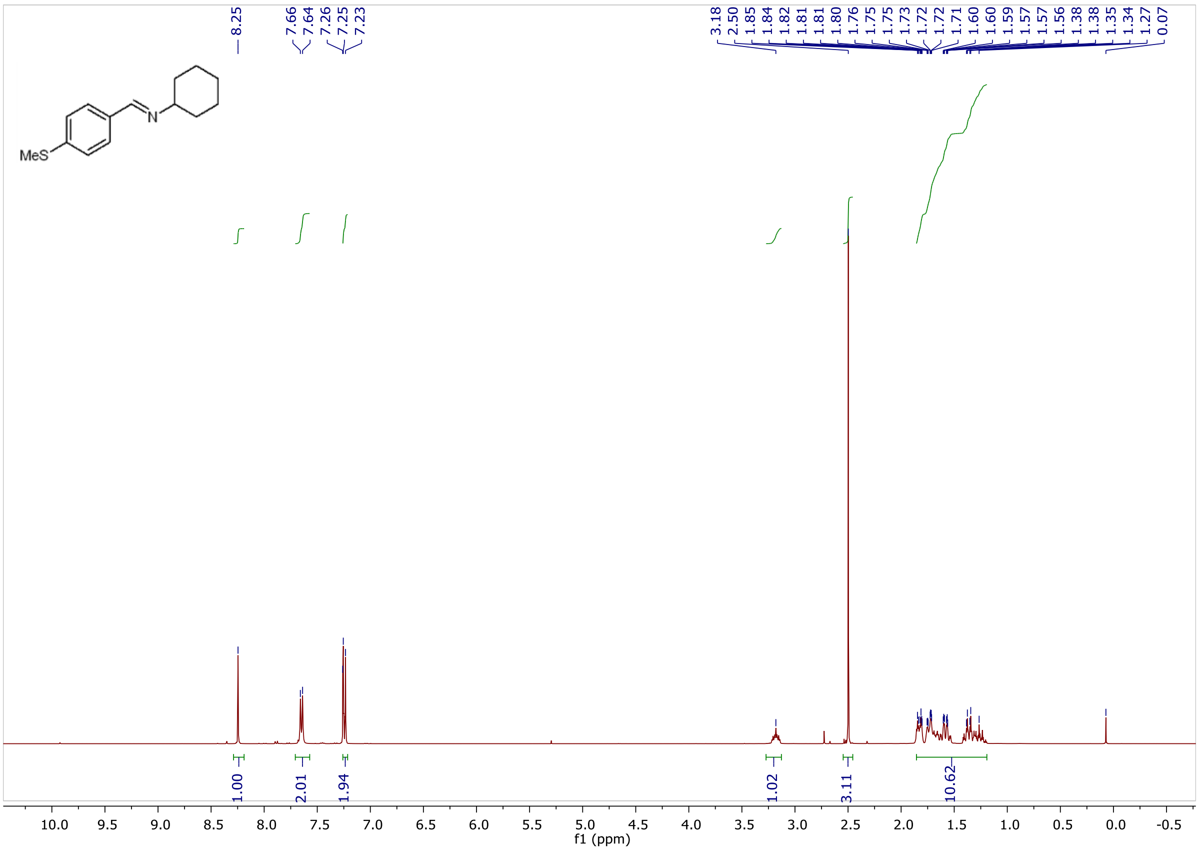
MeS
N
8.25
7.66
7.64
7.26
7.25
7.23
3.18
2.50
1.85
1.84
1.82
1.81
1.81
1.80
1.76
1.75
1.75
1.73
1.72
1.72
1.71
1.60
1.60
1.59
1.57
1.57
1.56
1.38
1.38
1.35
1.34
1.27
0.07
1.00
2.01
1.94
1.02
3.11
10.62
f1 (ppm)

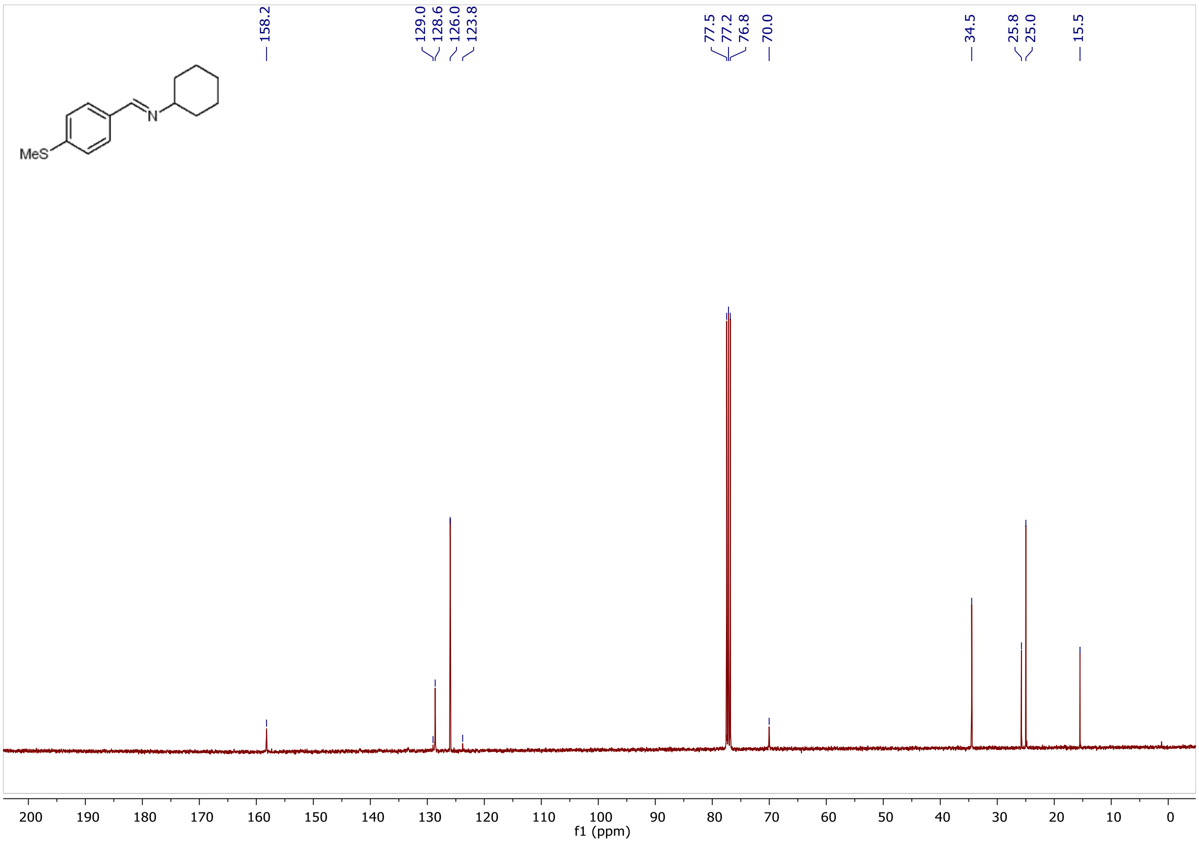
MeS
N
158.2
129.0
128.6
126.0
123.8
77.5
77.2
76.8
70.0
34.5
25.8
25.0
15.5
f1 (ppm)

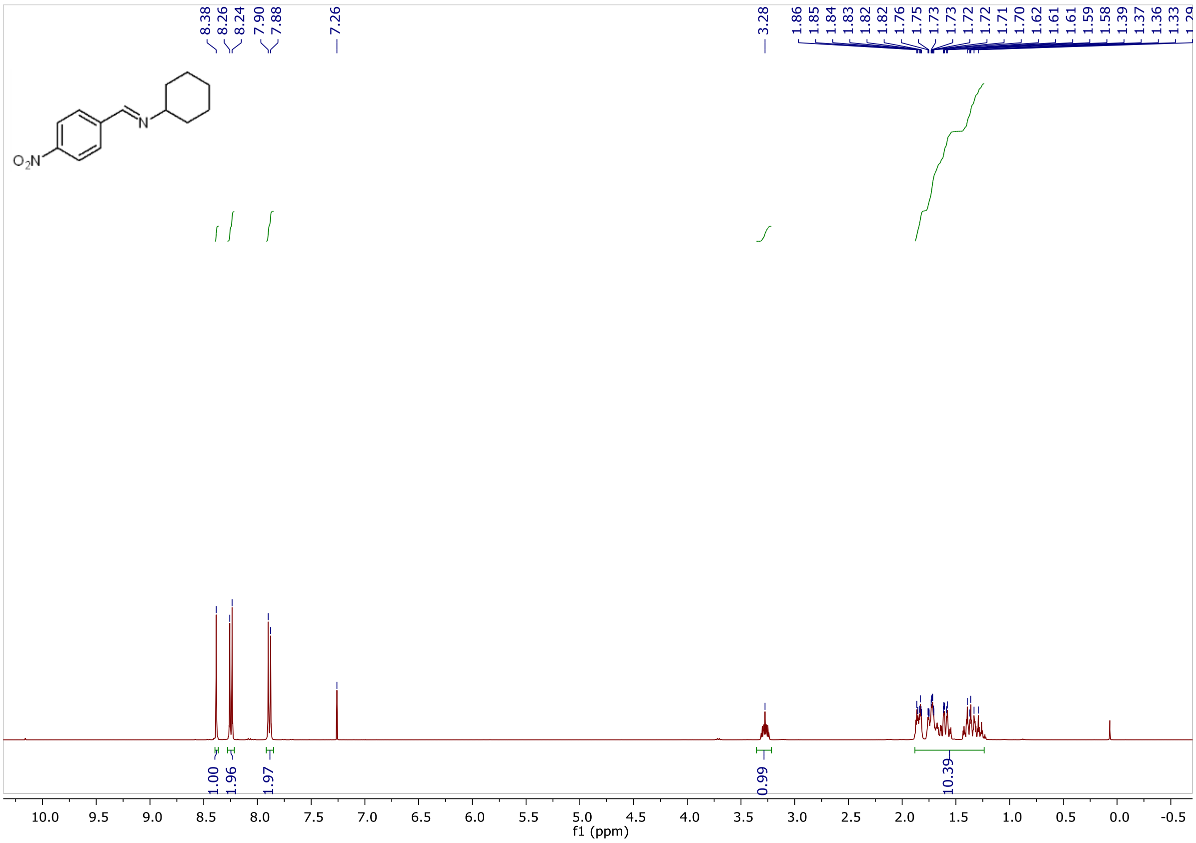

8.38
8.26
8.24
7.90
7.88
7.26
3.28
1.86
1.85
1.84
1.83
1.82
1.82
1.76
1.75
1.73
1.73
1.72
1.72
1.71
1.70
1.62
1.61
1.61
1.59
1.58
1.39
1.37
1.36
1.33
O2N
N
1.00
1.96
1.97
0.99
10.39
10.0 9.5 9.0 8.5 8.0 7.5 7.0 6.5 6.0 5.5 5.0 4.5 4.0 3.5 3.0 2.5 2.0 1.5 1.0 0.5 0.0 -0.5
f1 (ppm)

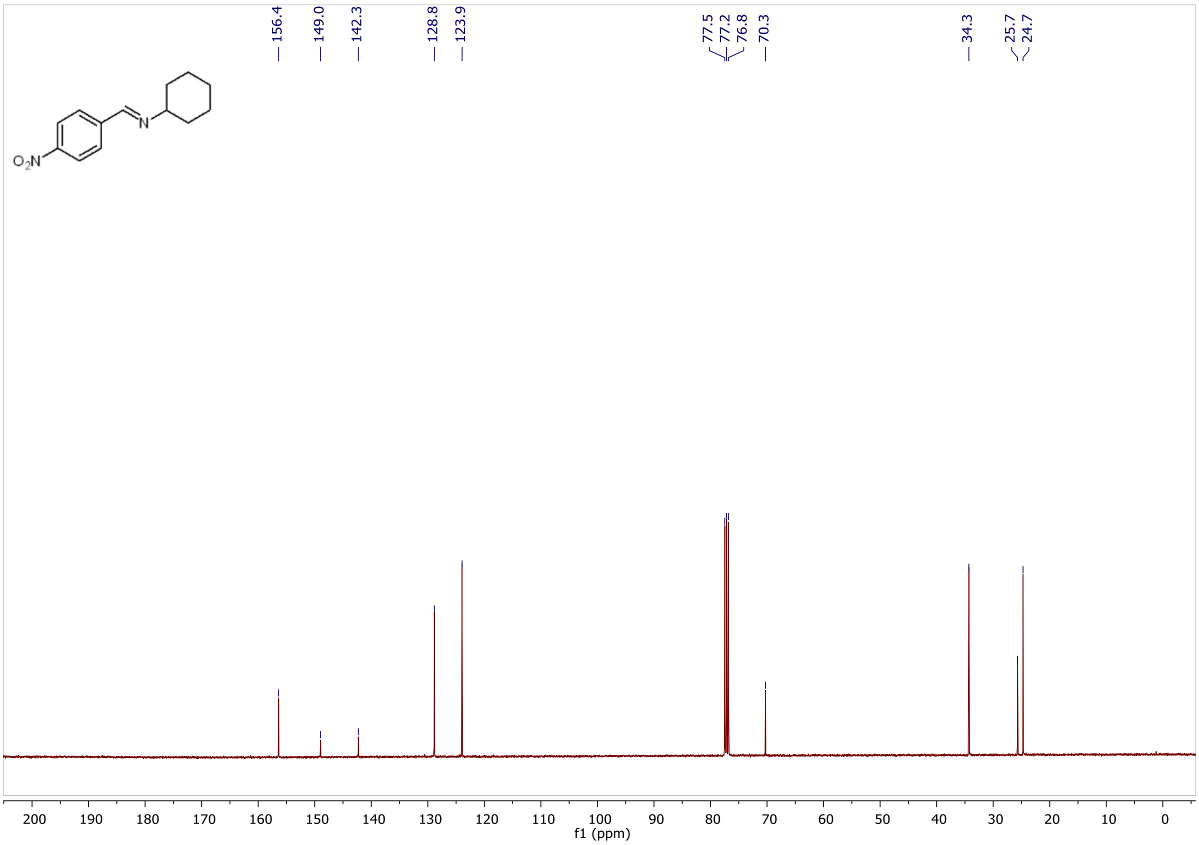

156.4
149.0
142.3
128.8
123.9
77.5
77.2
76.8
70.3
34.3
25.7
24.7
O2N
N
200 190 180 170 160 150 140 130 120 110 100 90 80 70 60 50 40 30 20 10 0
f1 (ppm)

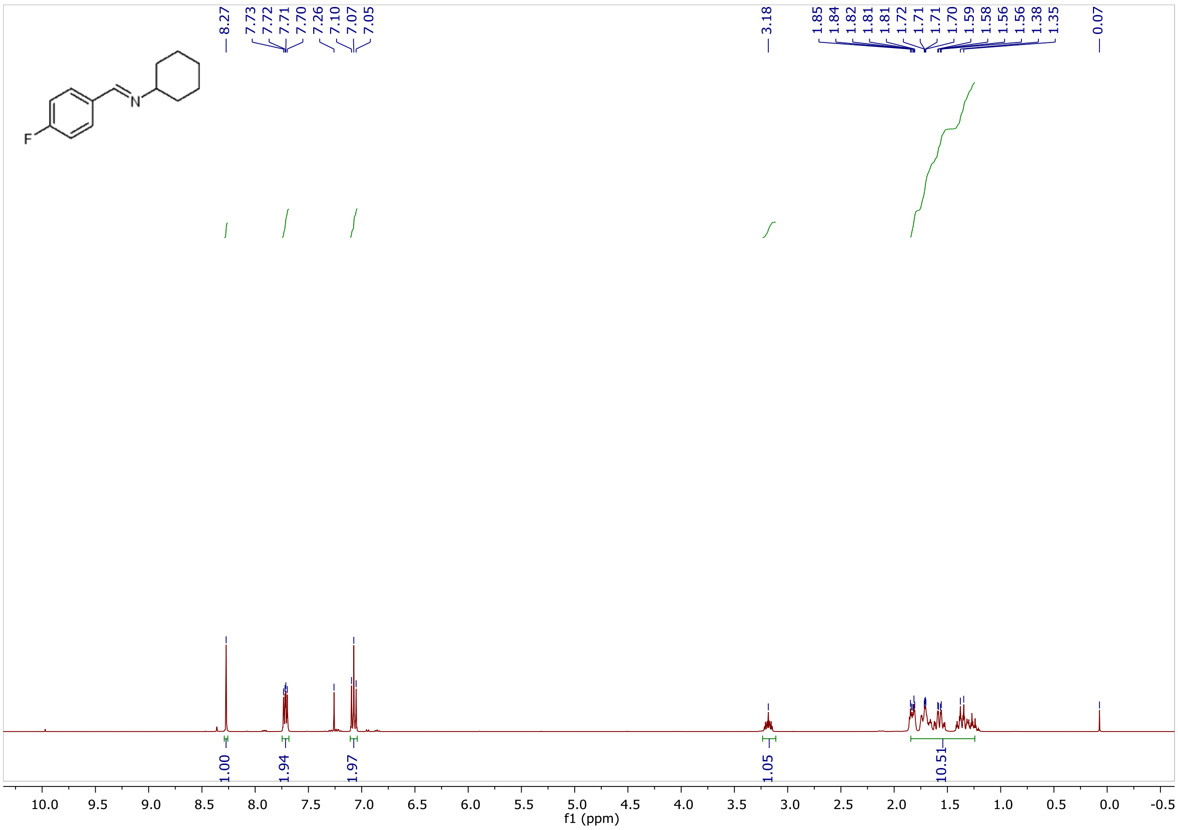
8.27
7.73
7.72
7.71
7.70
7.26
7.10
7.07
7.05
3.18
1.85
1.84
1.82
1.81
1.81
1.72
1.71
1.71
1.70
1.59
1.58
1.56
1.56
1.38
1.35
0.07
F
N
1.00
1.94
1.97
1.05
10.51
f1 (ppm)

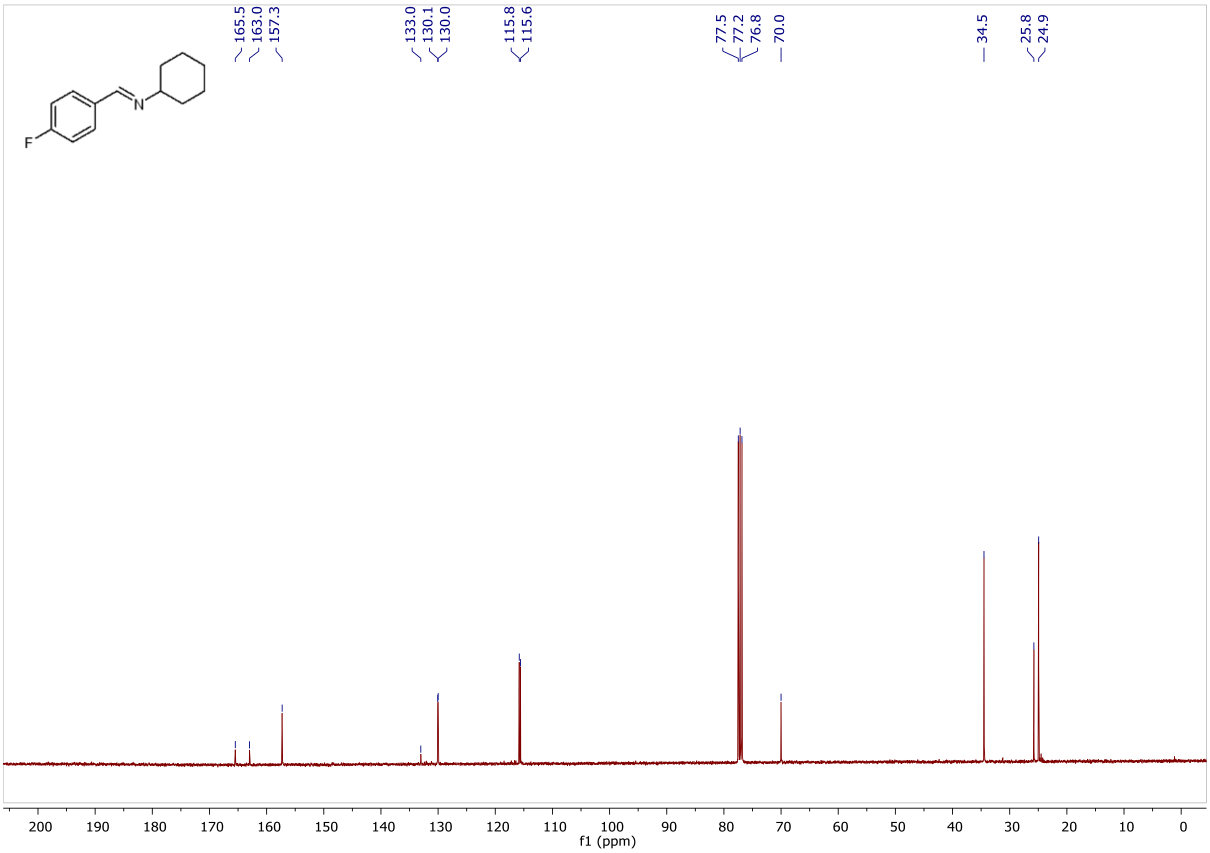
165.5
163.0
157.3
133.0
130.1
130.0
115.8
115.6
77.5
77.2
76.8
70.0
34.5
25.8
24.9
F
N
f1 (ppm)

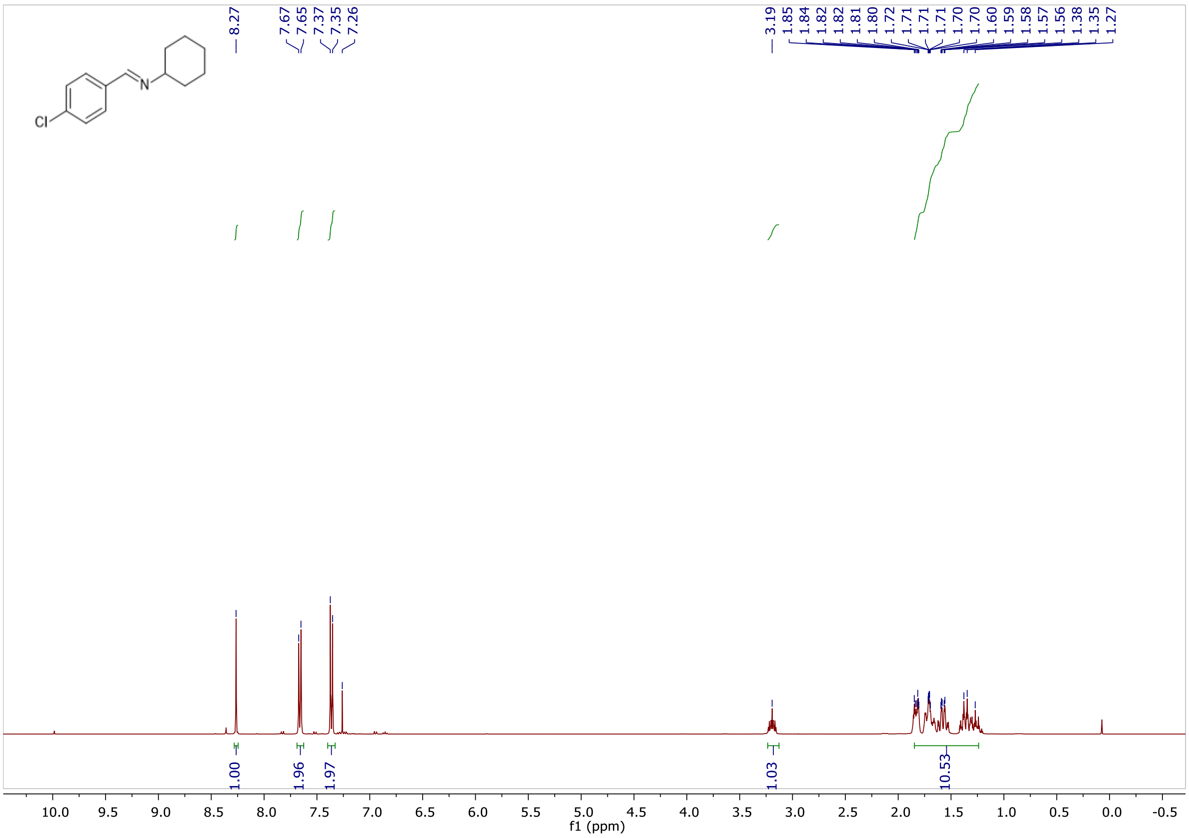
Cl
N
8.27
7.67
7.65
7.37
7.35
7.26
3.19
1.85
1.84
1.82
1.82
1.81
1.80
1.72
1.71
1.71
1.71
1.70
1.70
1.60
1.59
1.58
1.57
1.56
1.38
1.35
1.27
1.00
1.96
1.97
1.03
10.53
f1 (ppm)

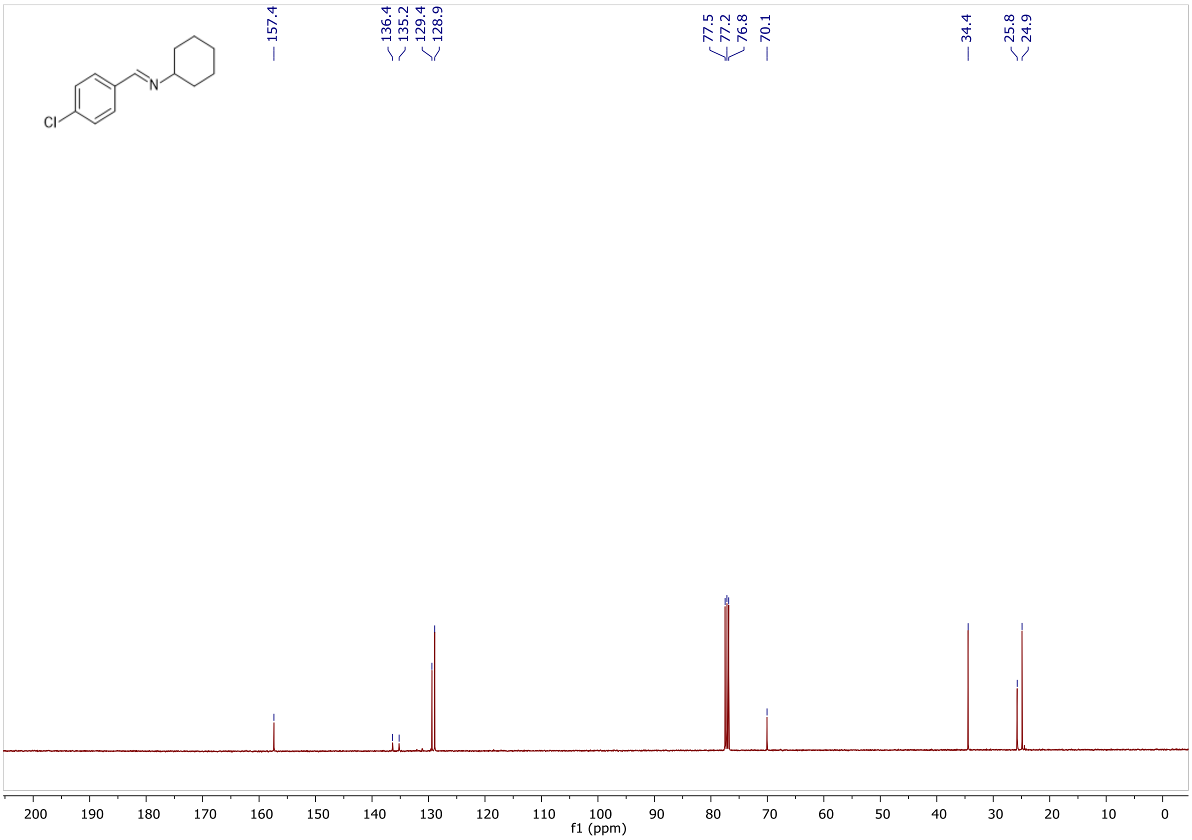
Cl
N
157.4
136.4
135.2
129.4
128.9
77.5
77.2
76.8
70.1
34.4
25.8
24.9
f1 (ppm)

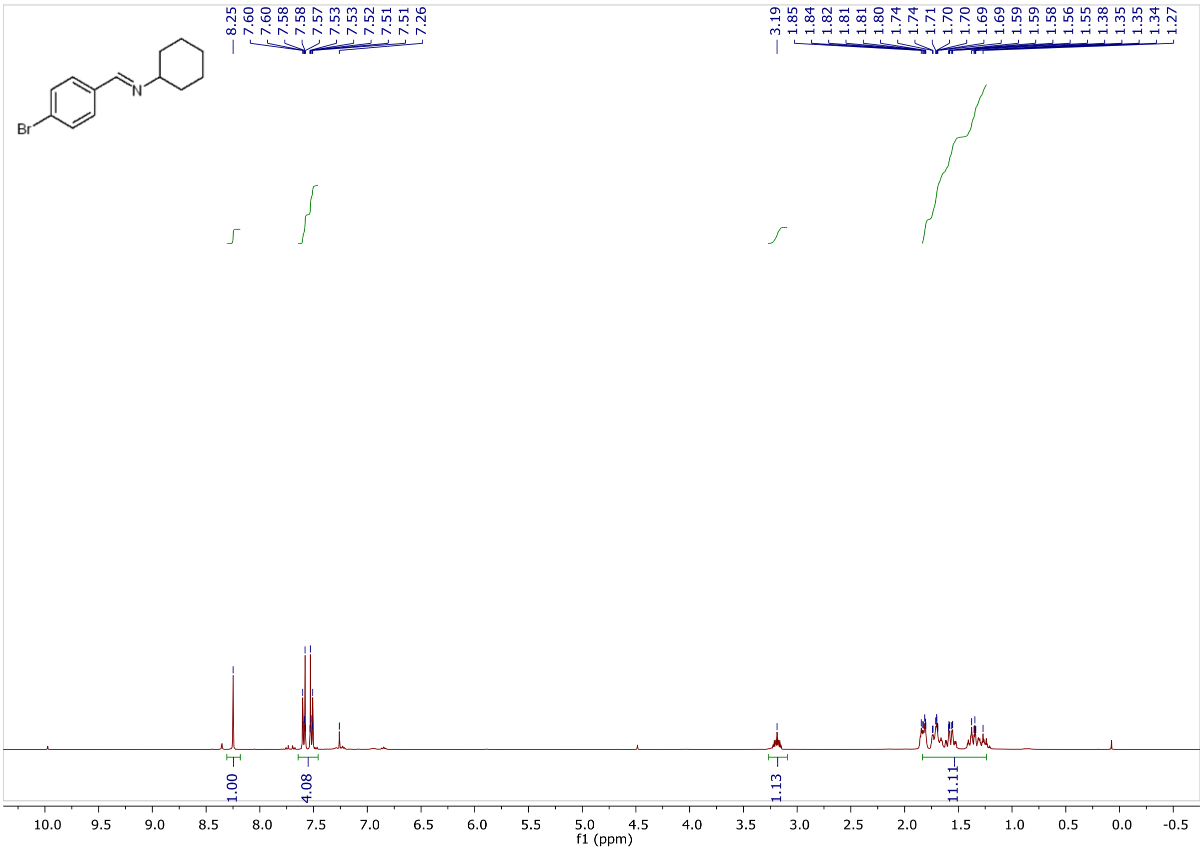

8.25
7.60
7.60
7.58
7.58
7.57
7.53
7.53
7.52
7.51
7.51
7.26
3.19
1.85
1.84
1.82
1.81
1.80
1.74
1.74
1.71
1.70
1.70
1.69
1.69
1.59
1.59
1.58
1.56
1.55
1.38
1.35
1.35
1.34
1.27
Br
N
1.00
4.08
1.13
11.11
f1 (ppm)

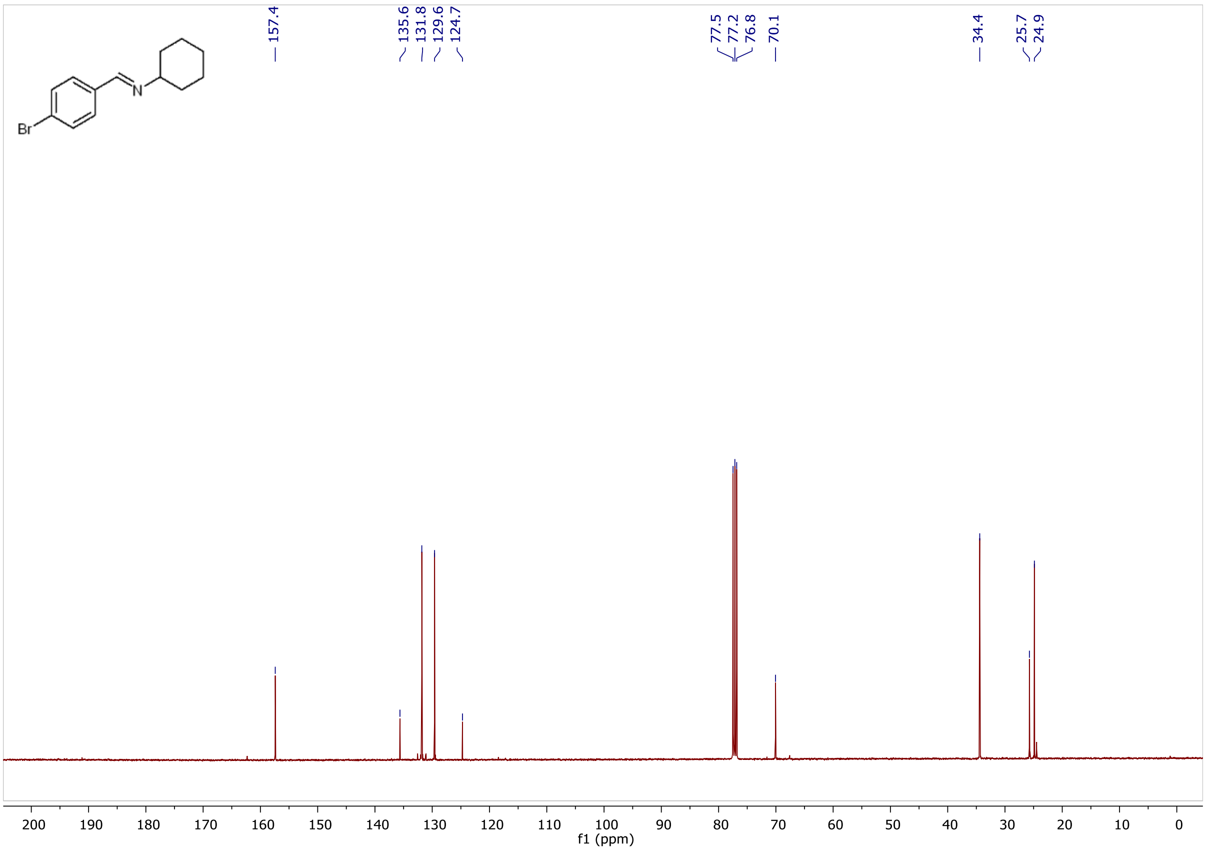

157.4
135.6
131.8
129.6
124.7
77.5
77.2
76.8
70.1
34.4
25.7
24.9
Br
N
f1 (ppm)

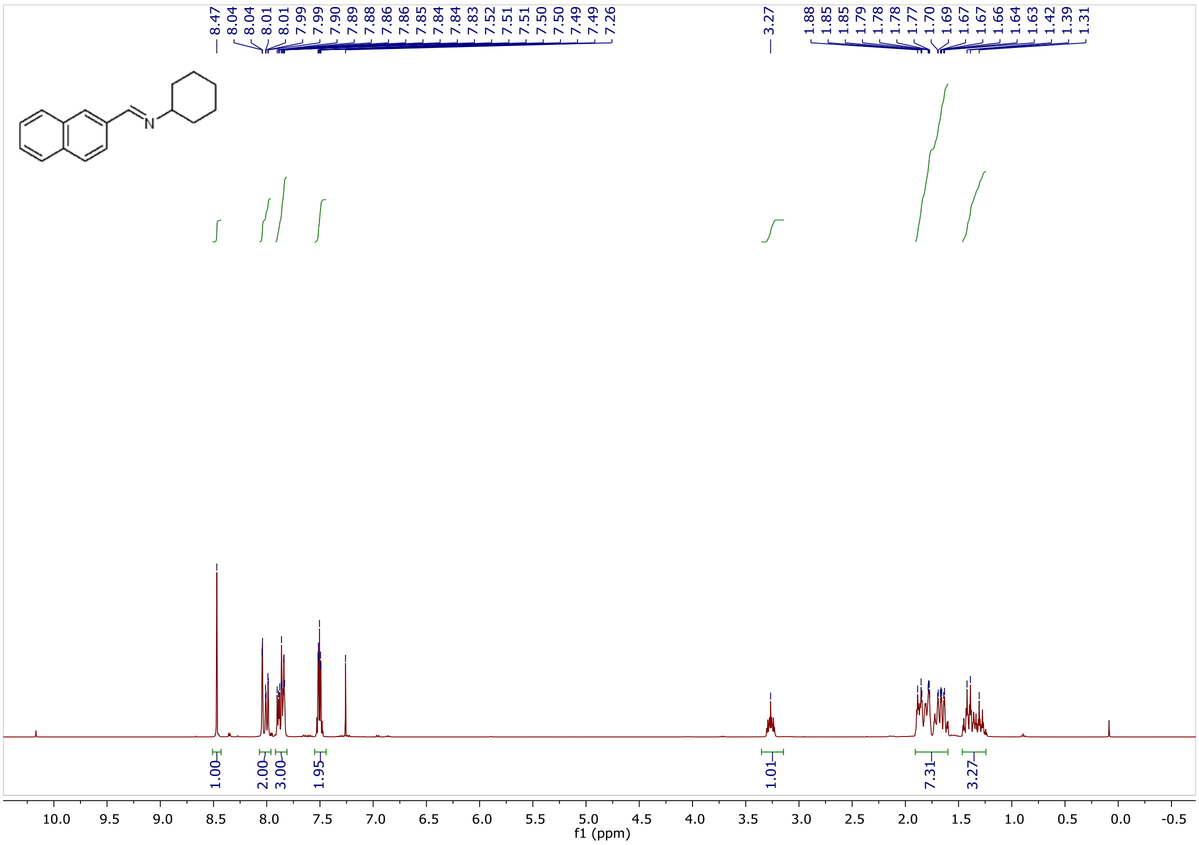
N
8.47
8.04
8.04
8.01
8.01
7.99
7.90
7.89
7.88
7.86
7.86
7.85
7.84
7.84
7.83
7.52
7.51
7.51
7.50
7.49
7.49
7.26
3.27
1.88
1.85
1.85
1.79
1.78
1.78
1.77
1.70
1.69
1.67
1.67
1.66
1.64
1.63
1.42
1.39
1.31
1.00
2.00
3.00
1.95
1.01
7.31
3.27
f1 (ppm)

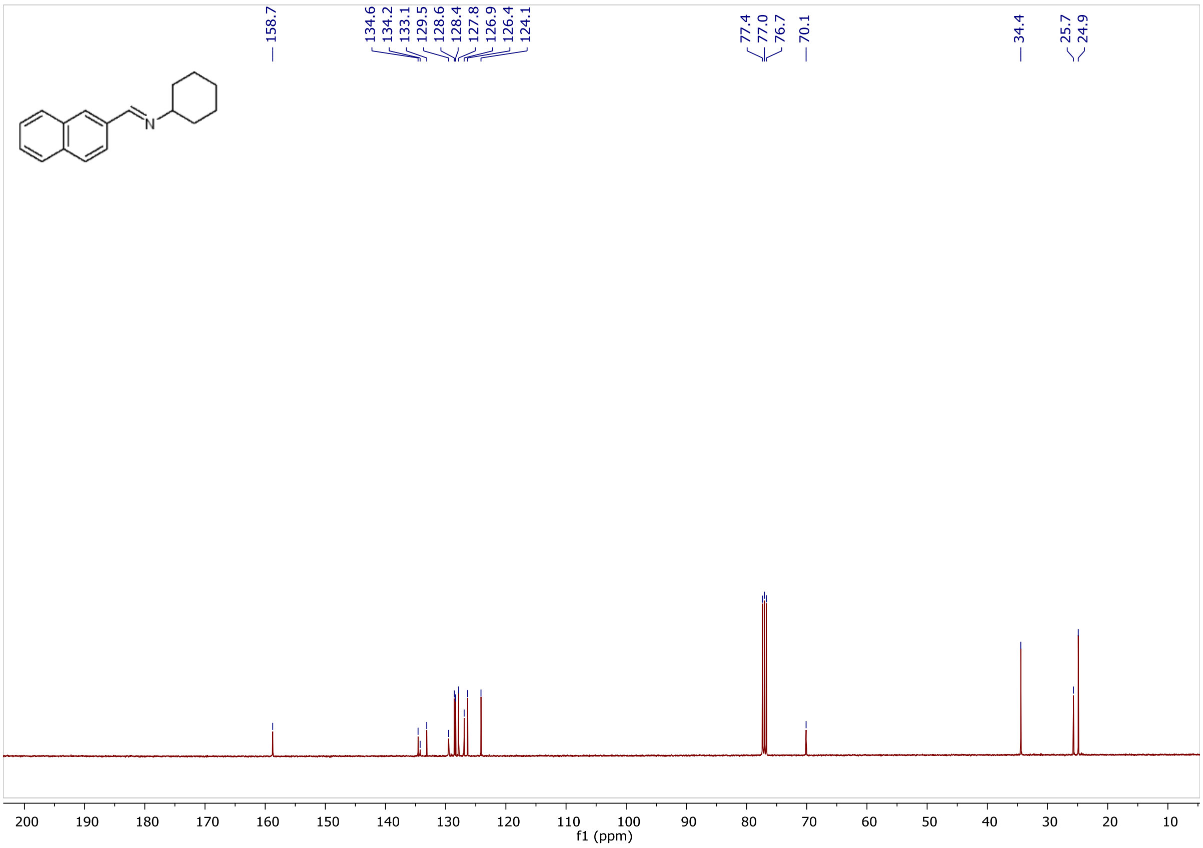
N
158.7
134.6
134.2
133.1
129.5
128.6
128.4
127.8
126.9
126.4
124.1
77.4
77.0
76.7
70.1
34.4
25.7
24.9
f1 (ppm)

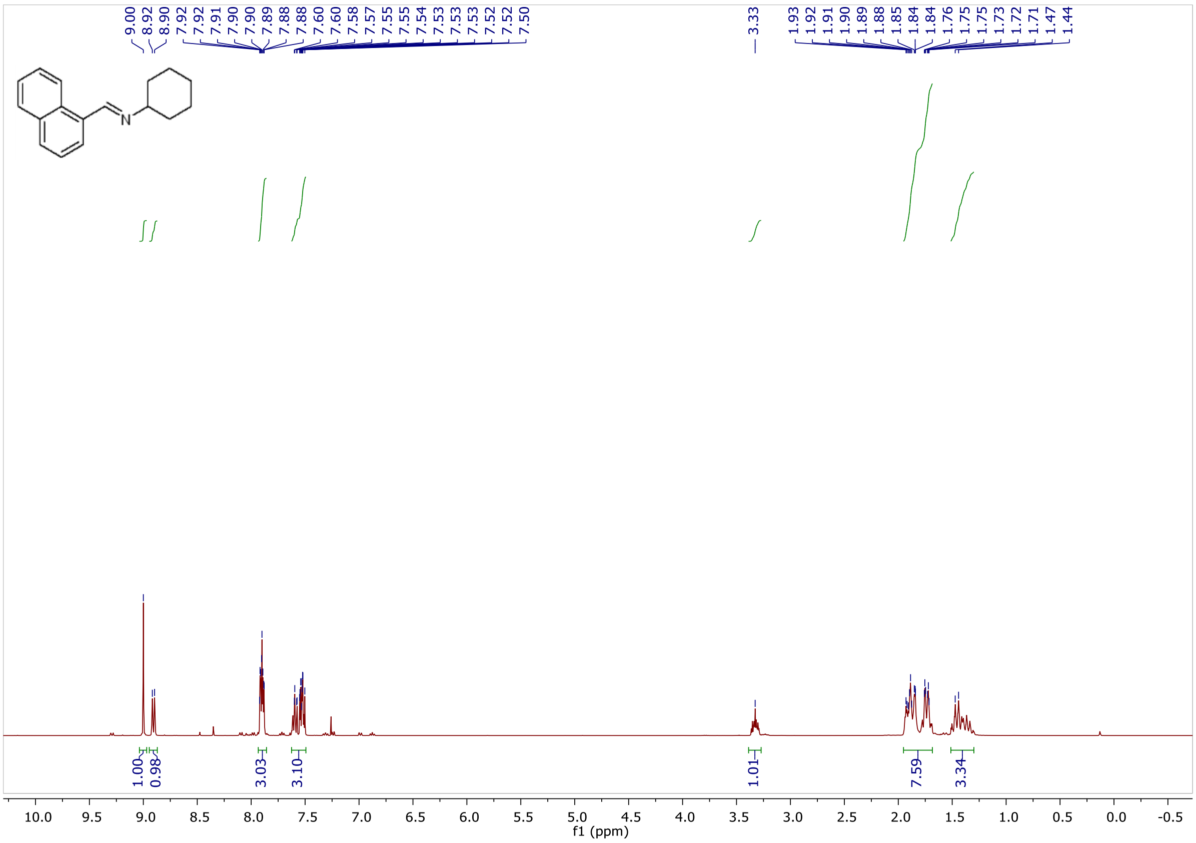

9.00
8.92
8.90
7.92
7.92
7.91
7.90
7.90
7.89
7.88
7.88
7.60
7.60
7.58
7.57
7.55
7.55
7.54
7.53
7.53
7.53
7.52
7.52
7.50
3.33
1.93
1.92
1.91
1.90
1.89
1.88
1.85
1.84
1.84
1.76
1.75
1.75
1.73
1.72
1.71
1.47
1.44
N
1.00
0.98
3.03
3.10
1.01
7.59
3.34
10.0 9.5 9.0 8.5 8.0 7.5 7.0 6.5 6.0 5.5 5.0 4.5 4.0 3.5 3.0 2.5 2.0 1.5 1.0 0.5 0.0 -0.5
f1 (ppm)

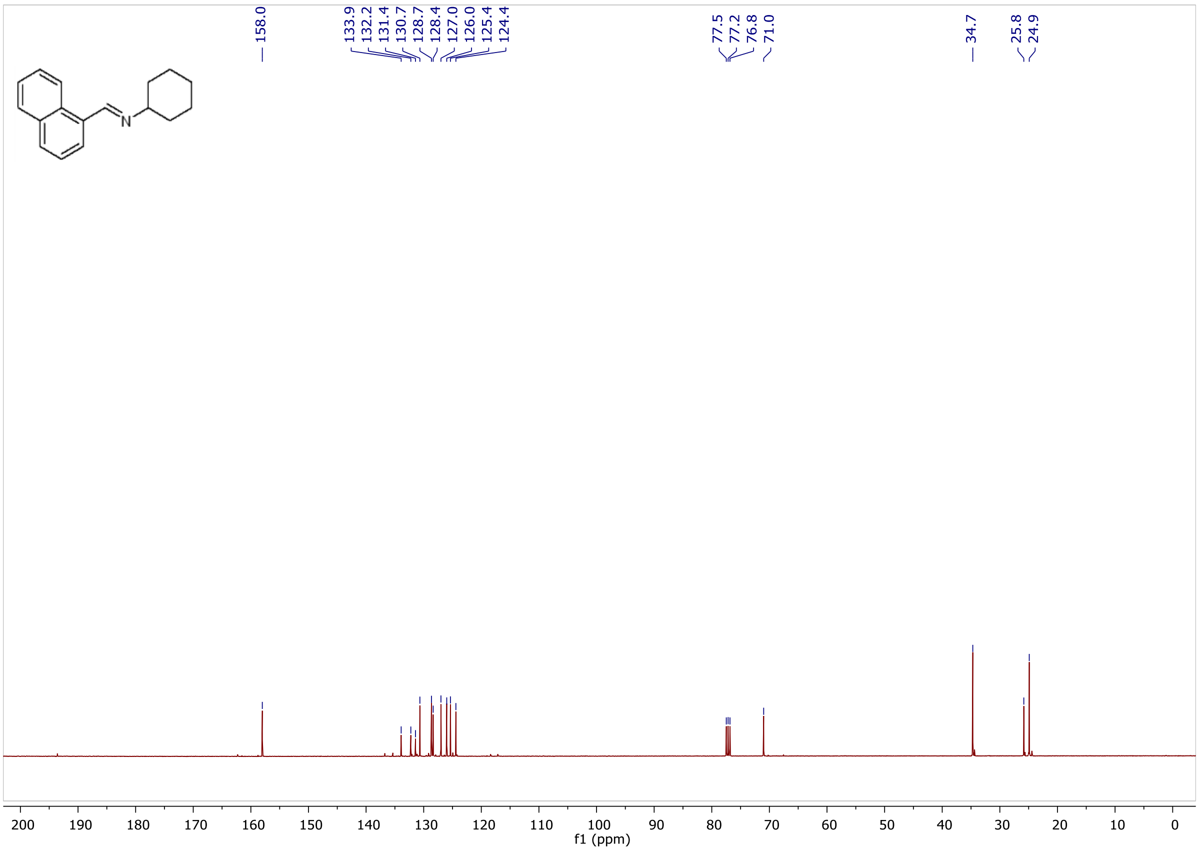

158.0
133.9
132.2
131.4
130.7
128.7
128.4
127.0
126.0
125.4
124.4
77.5
77.2
76.8
71.0
34.7
25.8
24.9
N
200 190 180 170 160 150 140 130 120 110 100 90 80 70 60 50 40 30 20 10 0
f1 (ppm)

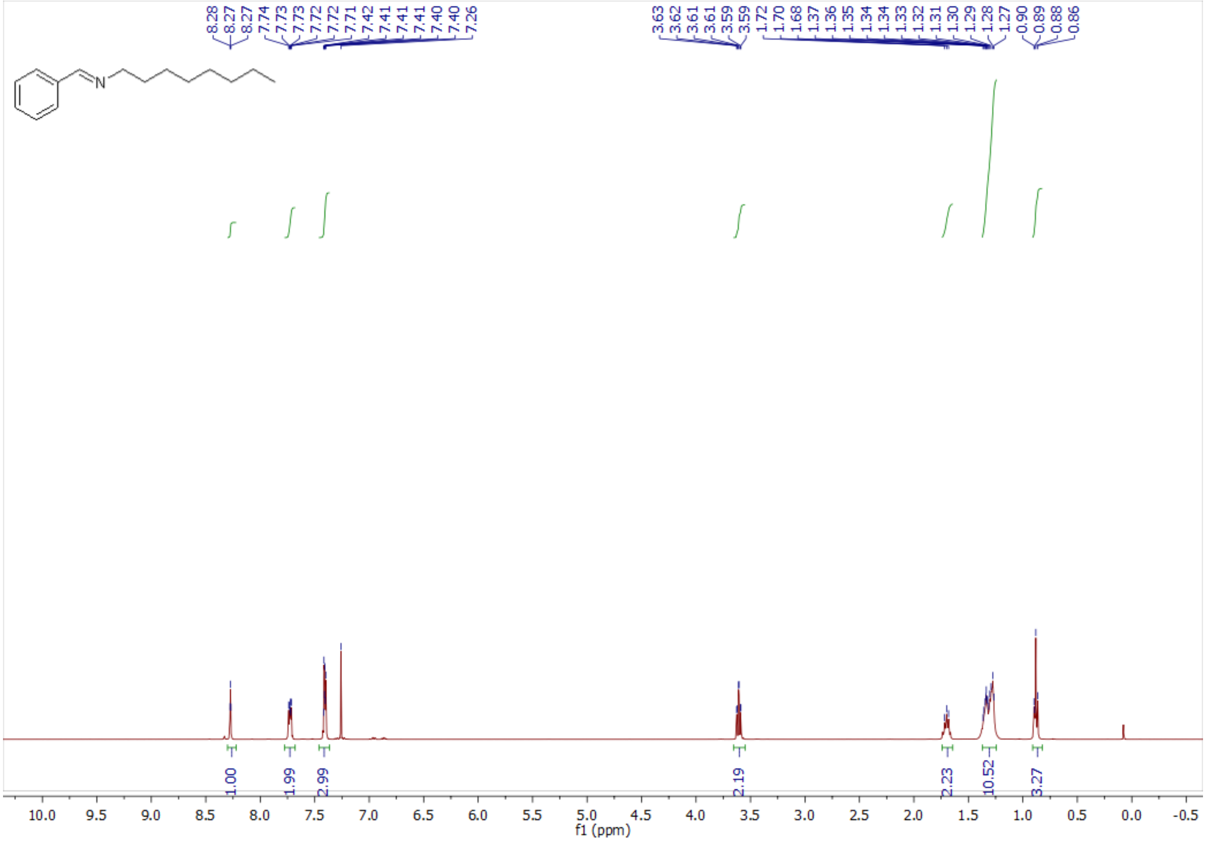
N
8.28
8.27
8.27
7.74
7.73
7.73
7.72
7.72
7.71
7.42
7.41
7.41
7.41
7.40
7.40
7.26
3.63
3.62
3.61
3.61
3.59
3.59
1.72
1.70
1.68
1.37
1.36
1.35
1.34
1.34
1.33
1.32
1.31
1.30
1.29
1.28
1.27
0.90
0.89
0.88
0.86
1.00
1.99
2.99
2.19
2.23
10.52
3.27
f1 (ppm)

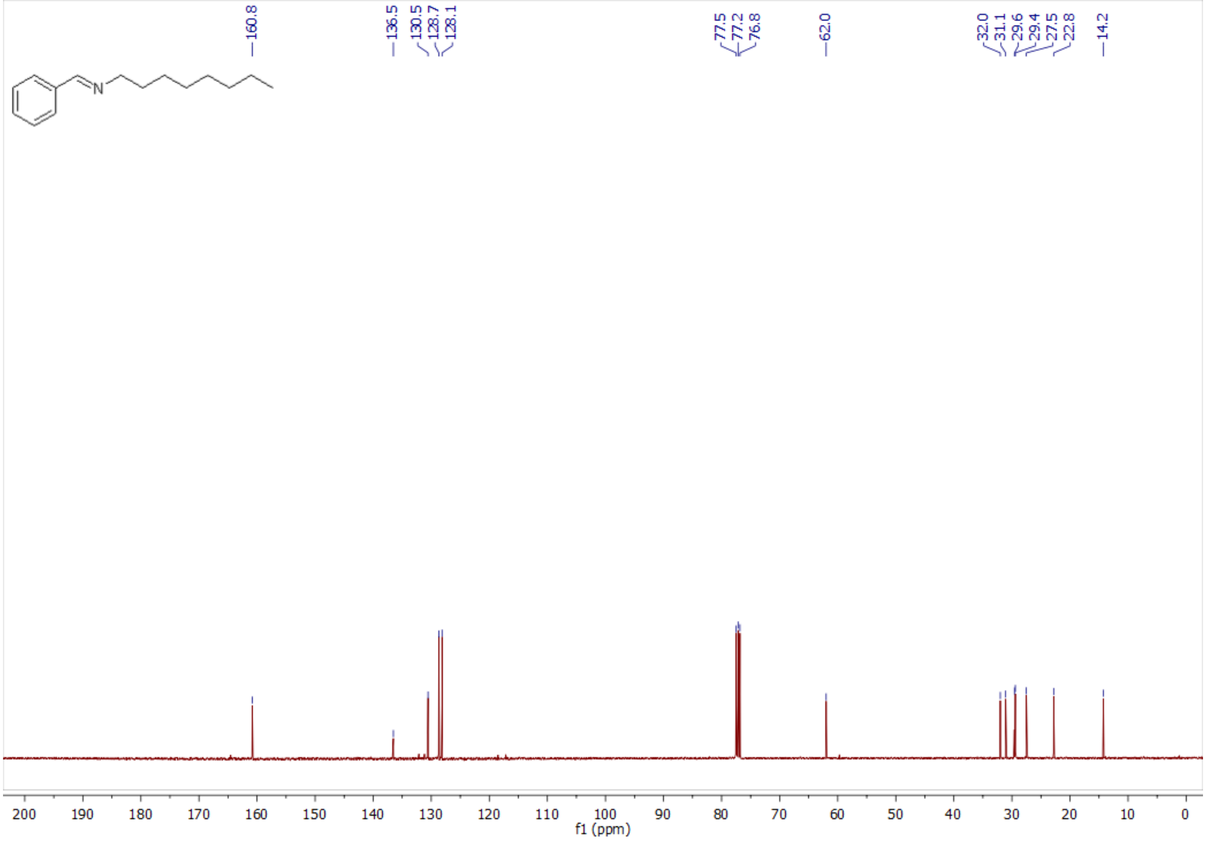
N
160.8
136.5
130.5
128.7
128.1
77.5
77.2
76.8
62.0
32.0
31.1
29.6
29.4
27.5
22.8
14.2
f1 (ppm)

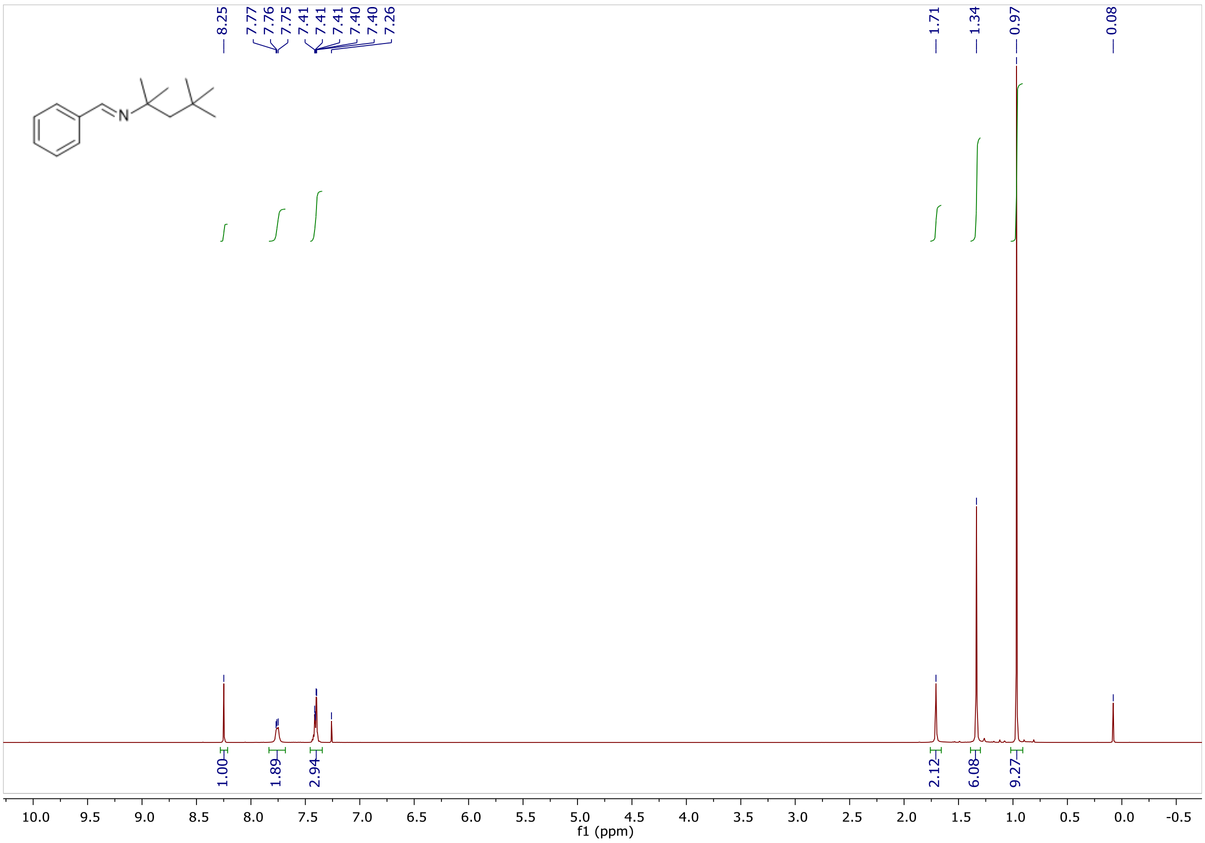
8.25
7.77
7.76
7.75
7.41
7.41
7.41
7.40
7.40
7.26
1.71
1.34
0.97
0.08
N
1.00
1.89
2.94
2.12
6.08
9.27
10.0
9.5
9.0
8.5
8.0
7.5
7.0
6.5
6.0
5.5
5.0
4.5
4.0
3.5
3.0
2.5
2.0
1.5
1.0
0.5
0.0
-0.5
f1 (ppm)

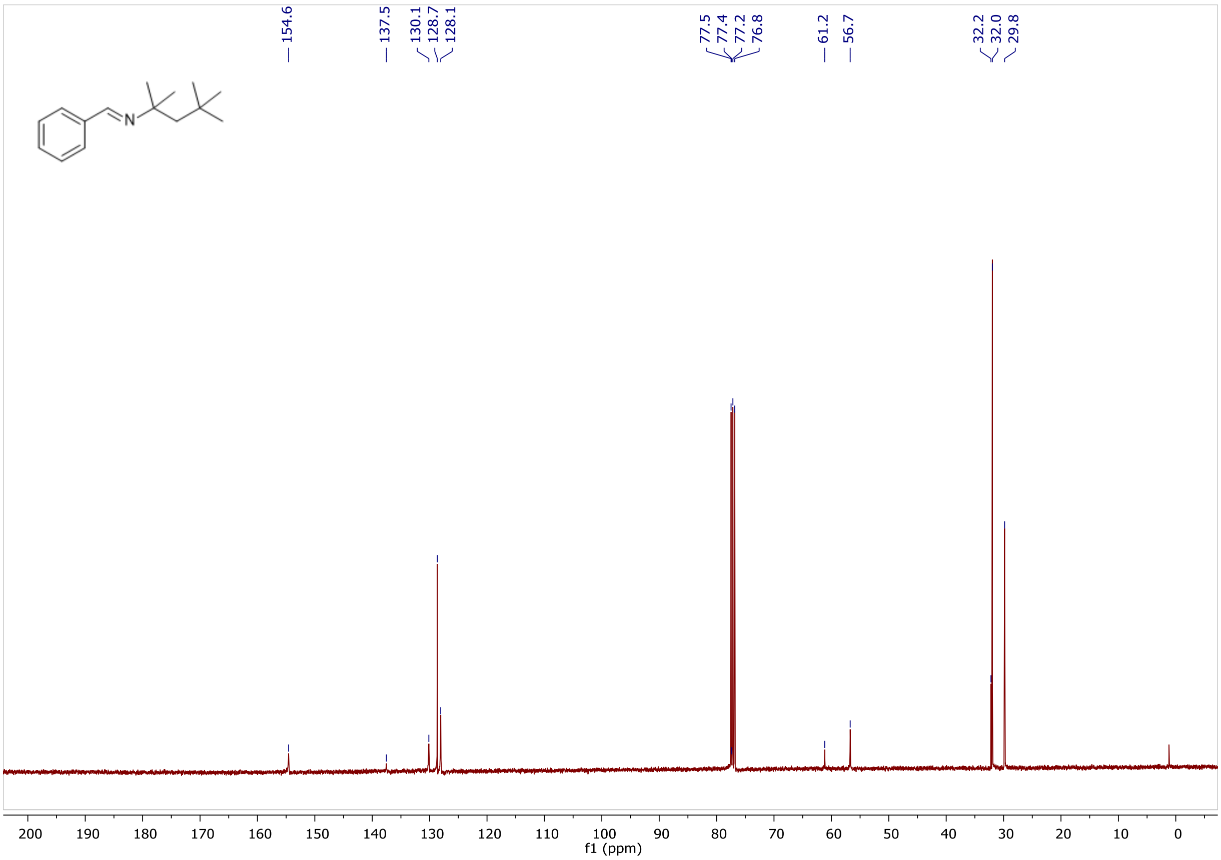
154.6
137.5
130.1
128.7
128.1
77.5
77.4
77.2
76.8
61.2
56.7
32.2
32.0
29.8
N
200
190
180
170
160
150
140
130
120
110
100
90
80
70
60
50
40
30
20
10
0
f1 (ppm)

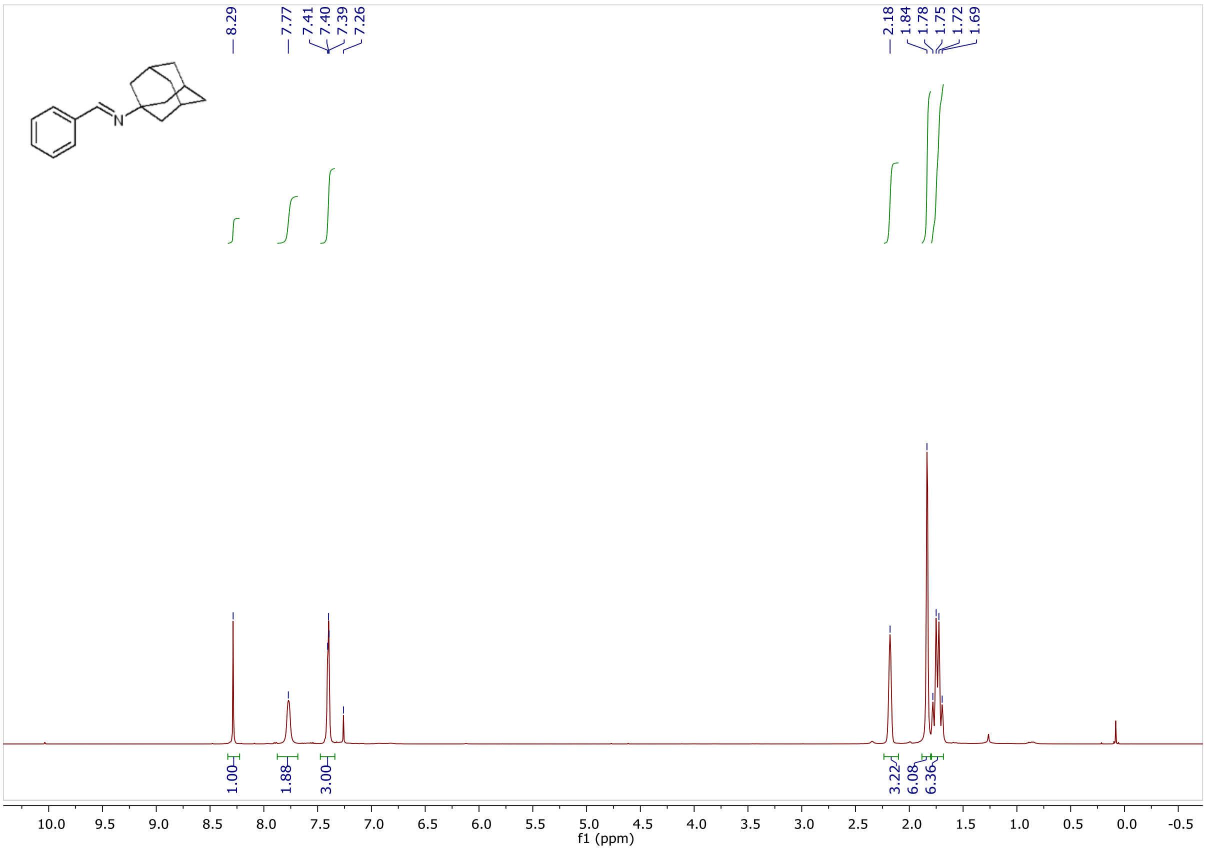
8.29
7.77
7.41
7.40
7.39
7.26
2.18
1.84
1.78
1.75
1.72
1.69
1.00
1.88
3.00
3.22
6.08
6.36
N
10.0 9.5 9.0 8.5 8.0 7.5 7.0 6.5 6.0 5.5 5.0 4.5 4.0 3.5 3.0 2.5 2.0 1.5 1.0 0.5 0.0 -0.5
f1 (ppm)

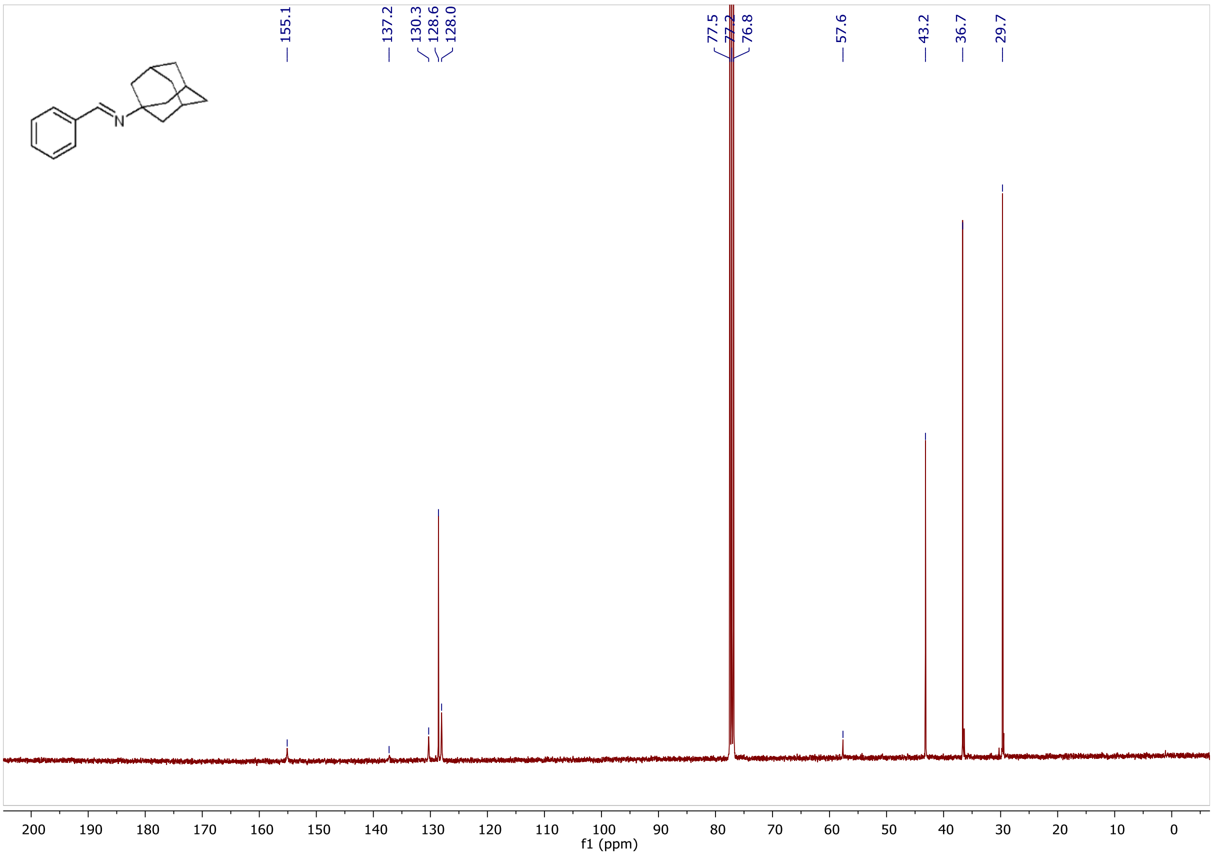
155.1
137.2
130.3
128.6
128.0
77.5
77.2
76.8
57.6
43.2
36.7
29.7
N
200 190 180 170 160 150 140 130 120 110 100 90 80 70 60 50 40 30 20 10 0
f1 (ppm)

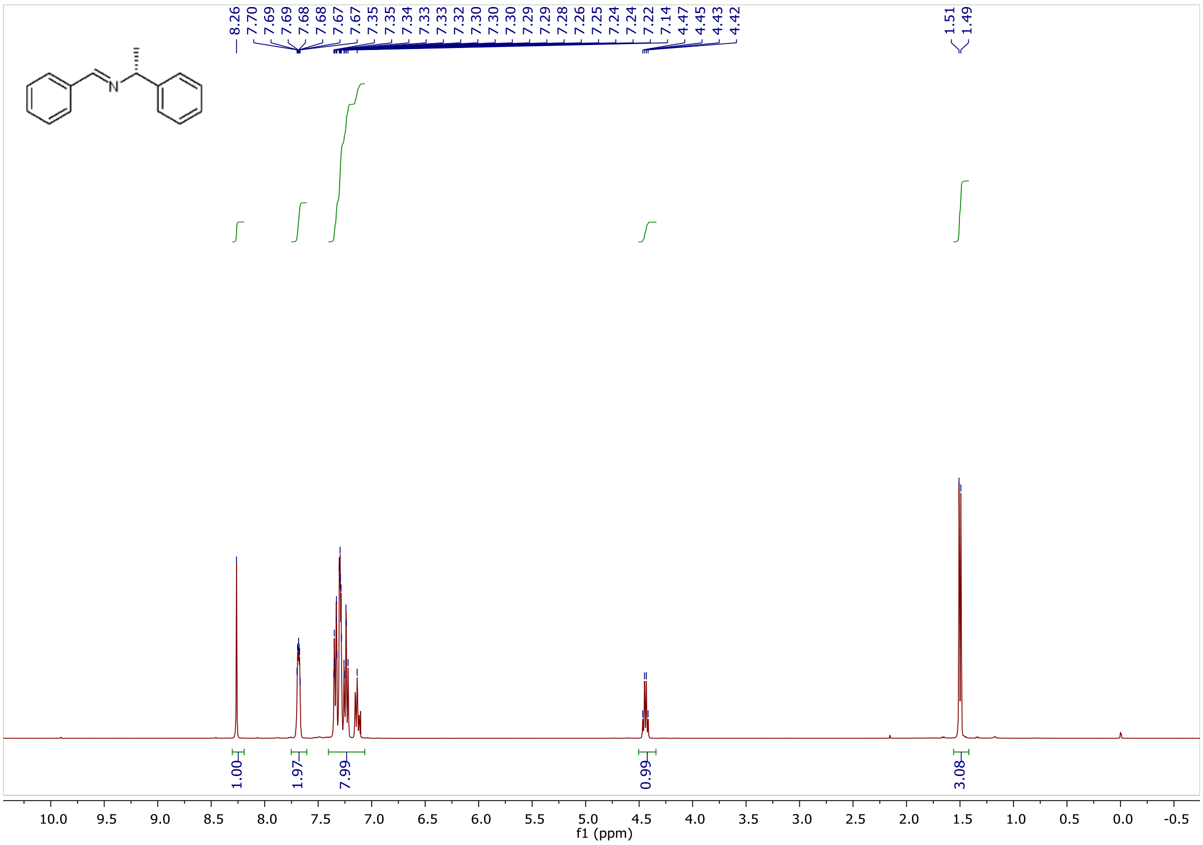
8.26
7.70
7.69
7.69
7.68
7.68
7.67
7.67
7.35
7.35
7.34
7.33
7.33
7.32
7.30
7.30
7.30
7.29
7.29
7.28
7.26
7.25
7.24
7.24
7.22
7.14
4.47
4.45
4.43
4.42
1.51
1.49
1.00
1.97
7.99
0.99
3.08
f1 (ppm)

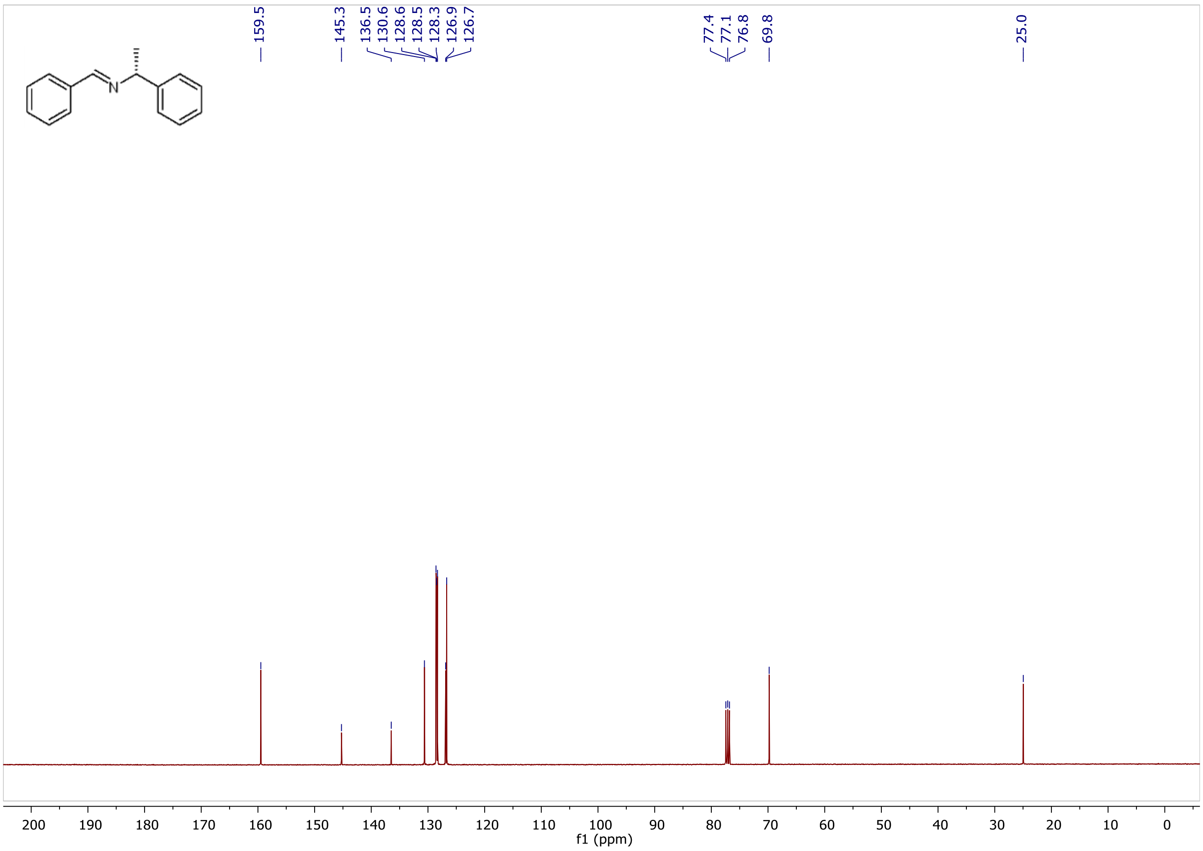
159.5
145.3
136.5
130.6
128.6
128.5
128.3
126.9
126.7
77.4
77.1
76.8
69.8
25.0
f1 (ppm)

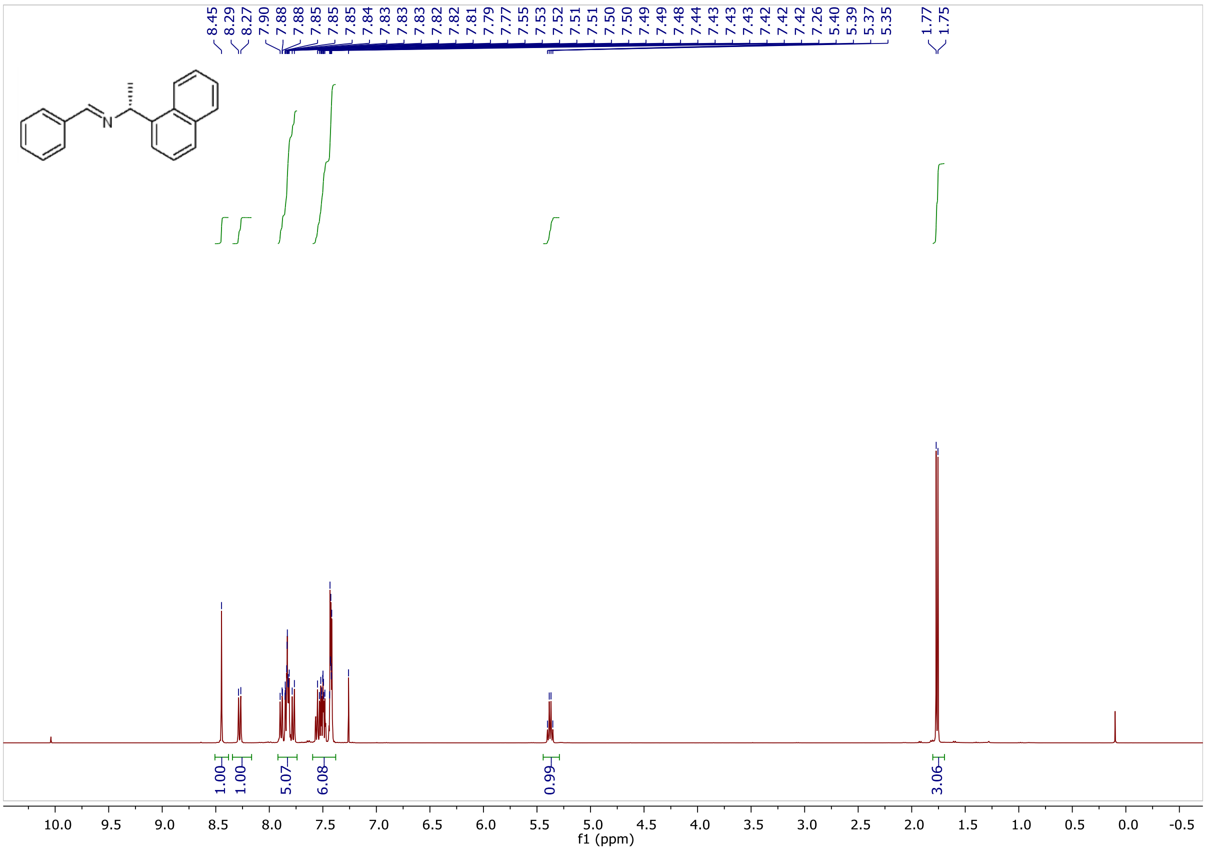

8.45
8.29
8.27
7.90
7.88
7.88
7.85
7.85
7.85
7.84
7.83
7.83
7.83
7.82
7.82
7.81
7.79
7.77
7.55
7.53
7.52
7.51
7.51
7.50
7.50
7.49
7.49
7.48
7.44
7.43
7.43
7.43
7.42
7.42
7.42
7.26
5.40
5.39
5.37
5.35
1.77
1.75
N
1.00
1.00
5.07
6.08
0.99
3.06
10.0 9.5 9.0 8.5 8.0 7.5 7.0 6.5 6.0 5.5 5.0 4.5 4.0 3.5 3.0 2.5 2.0 1.5 1.0 0.5 0.0 -0.5
f1 (ppm)

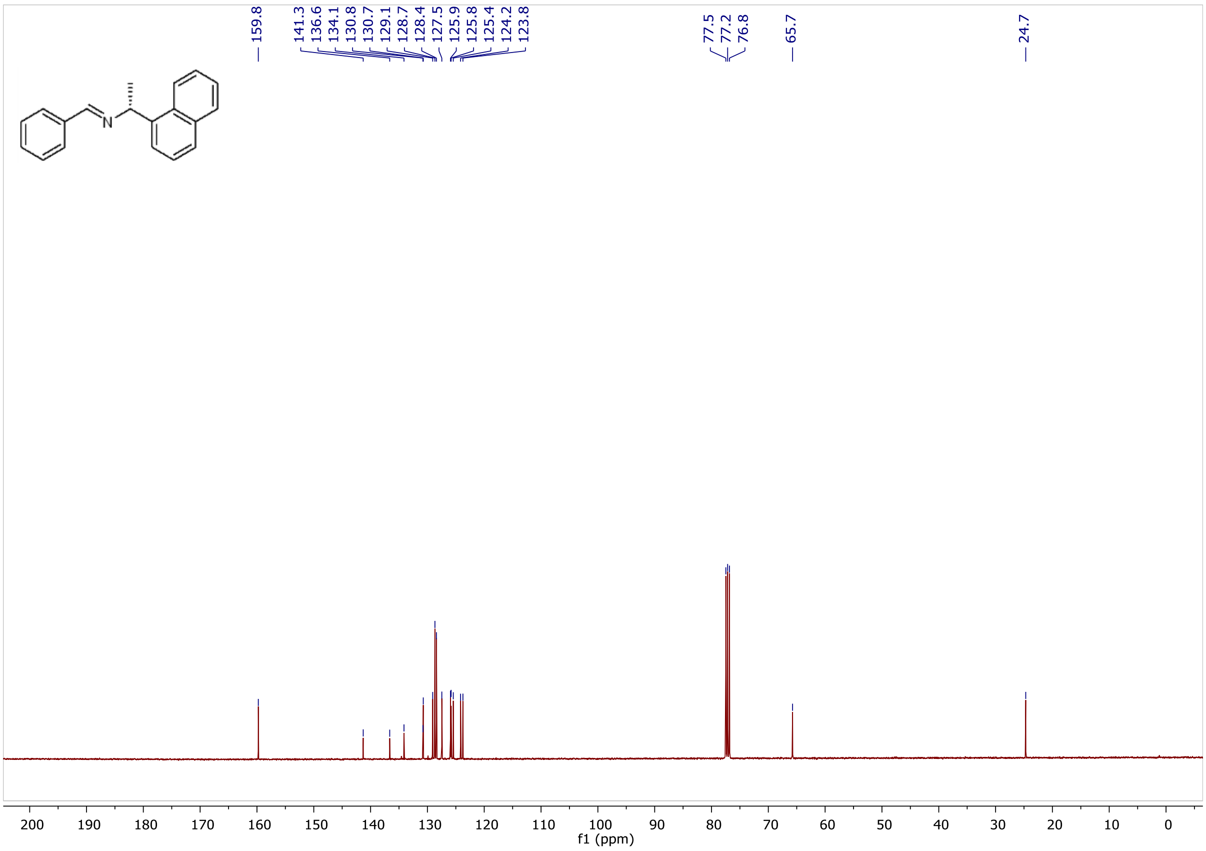

159.8
141.3
136.6
134.1
130.8
130.7
129.1
128.7
128.4
127.5
125.9
125.8
125.4
124.2
123.8
77.5
77.2
76.8
65.7
24.7
N
200 190 180 170 160 150 140 130 120 110 100 90 80 70 60 50 40 30 20 10 0
f1 (ppm)

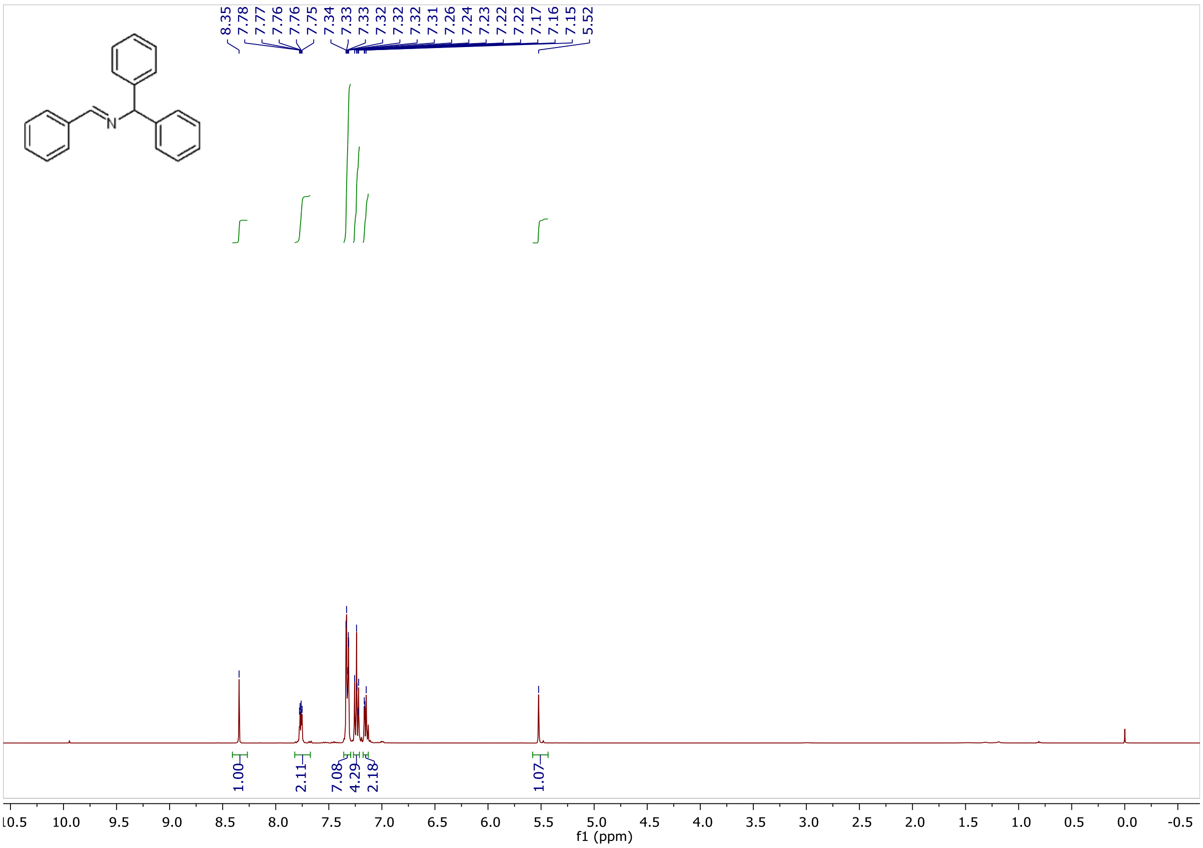
N
8.35
7.78
7.77
7.76
7.76
7.75
7.34
7.33
7.33
7.32
7.32
7.32
7.31
7.26
7.24
7.23
7.22
7.22
7.17
7.16
7.15
5.52
1.00
2.11
7.08
4.29
2.18
1.07
10.5 10.0 9.5 9.0 8.5 8.0 7.5 7.0 6.5 6.0 5.5 5.0 4.5 4.0 3.5 3.0 2.5 2.0 1.5 1.0 0.5 0.0 -0.5
f1 (ppm)

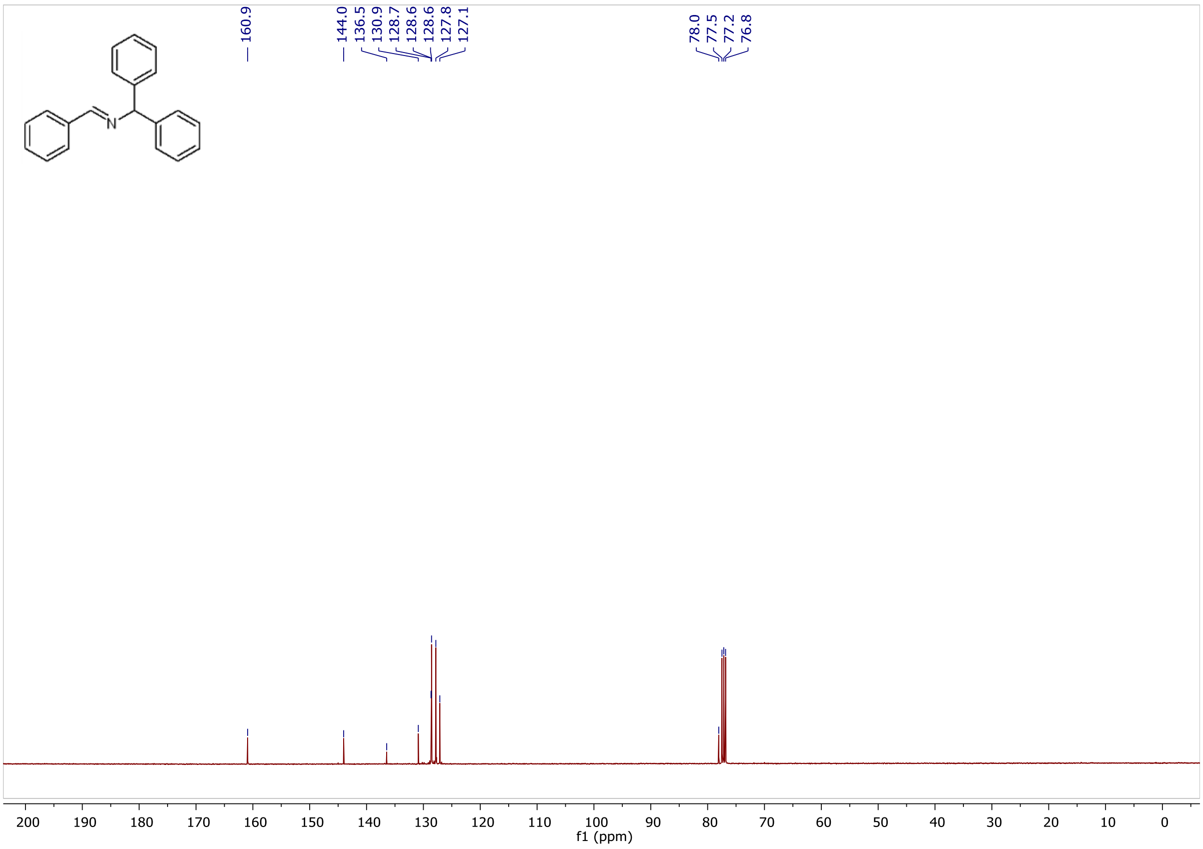
N
160.9
144.0
136.5
130.9
128.7
128.6
128.6
127.8
127.1
78.0
77.5
77.2
76.8
200 190 180 170 160 150 140 130 120 110 100 90 80 70 60 50 40 30 20 10 0
f1 (ppm)

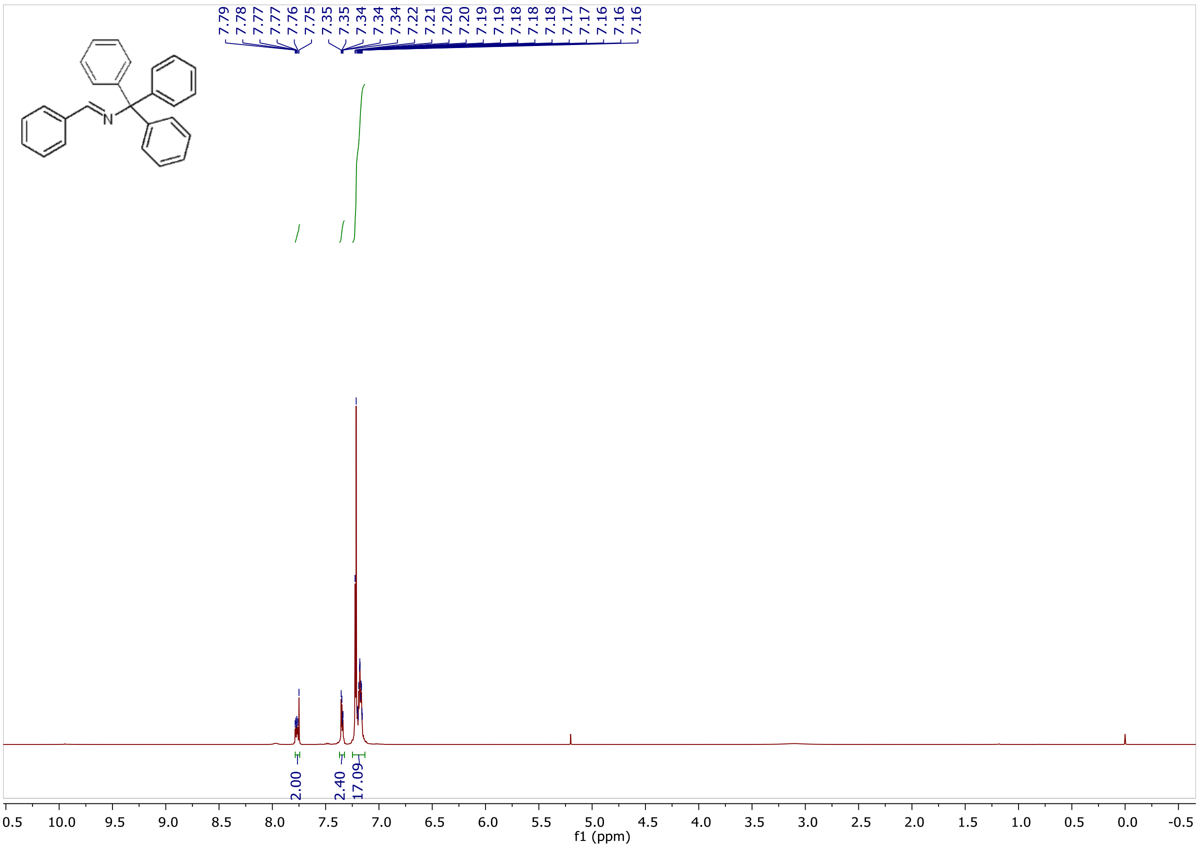
7.79
7.78
7.77
7.77
7.76
7.75
7.35
7.35
7.34
7.34
7.34
7.22
7.21
7.20
7.20
7.19
7.19
7.18
7.18
7.18
7.17
7.17
7.16
7.16
7.16
N
2.00
2.40
17.09
f1 (ppm)

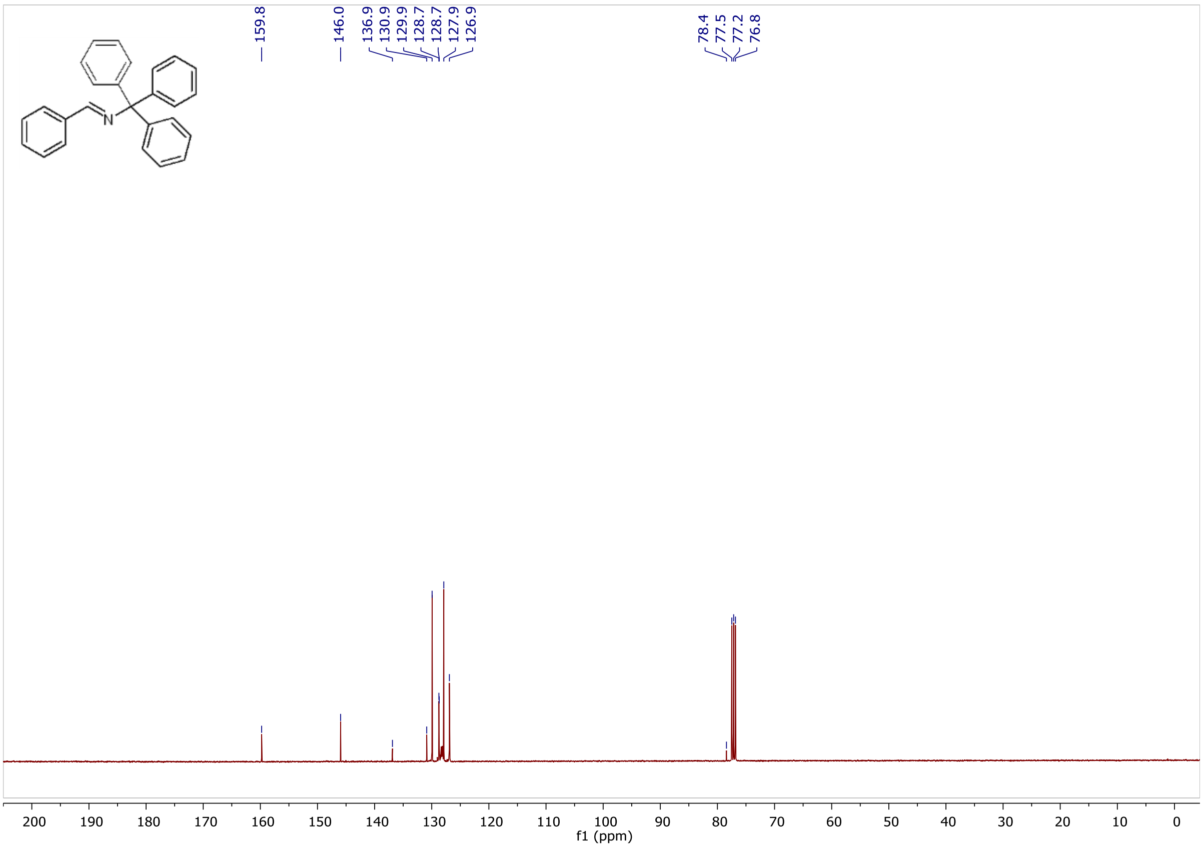
159.8
146.0
136.9
130.9
129.9
128.7
128.7
127.9
126.9
78.4
77.5
77.2
76.8
N
f1 (ppm)

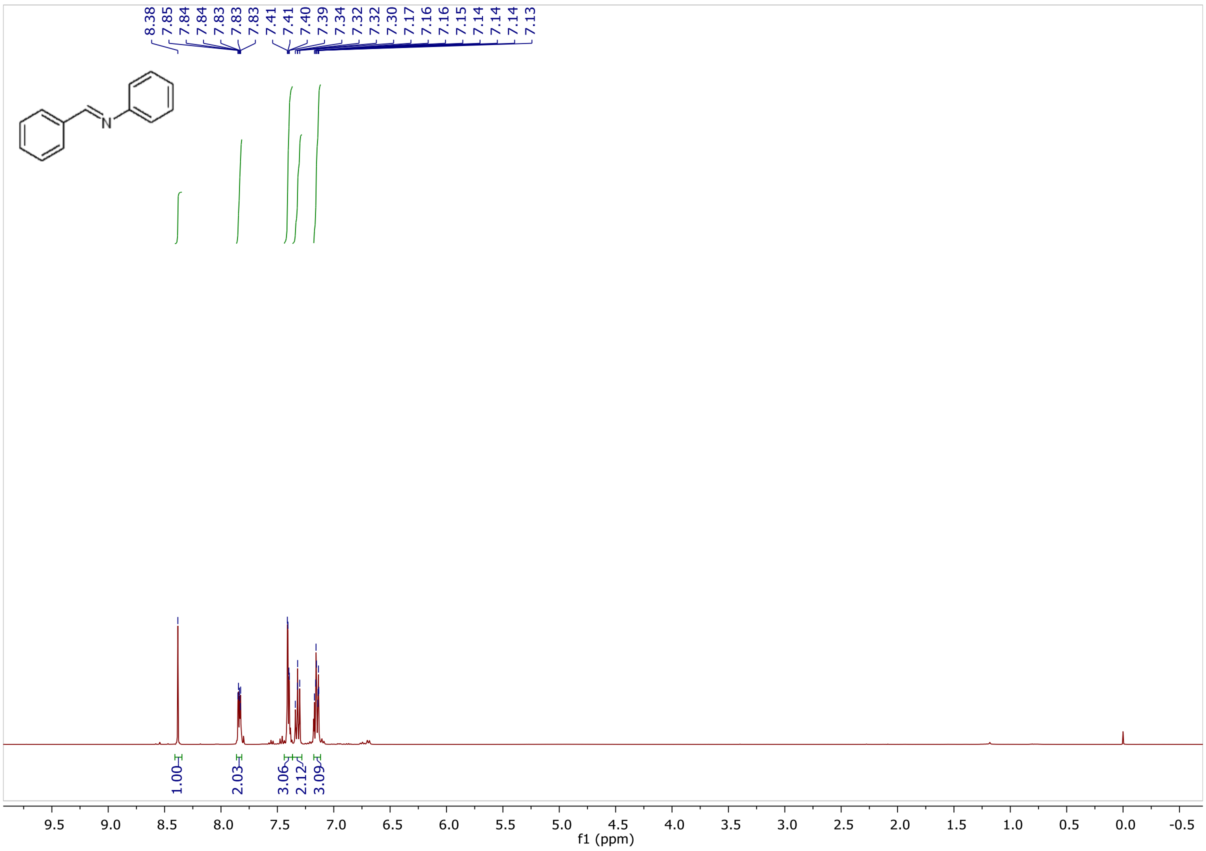
8.38
7.85
7.84
7.84
7.83
7.83
7.83
7.41
7.41
7.40
7.39
7.34
7.32
7.30
7.17
7.16
7.16
7.15
7.14
7.14
7.14
7.13
N
1.00
2.03
3.06
2.12
3.09
9.5 9.0 8.5 8.0 7.5 7.0 6.5 6.0 5.5 5.0 4.5 4.0 3.5 3.0 2.5 2.0 1.5 1.0 0.5 0.0 -0.5
f1 (ppm)

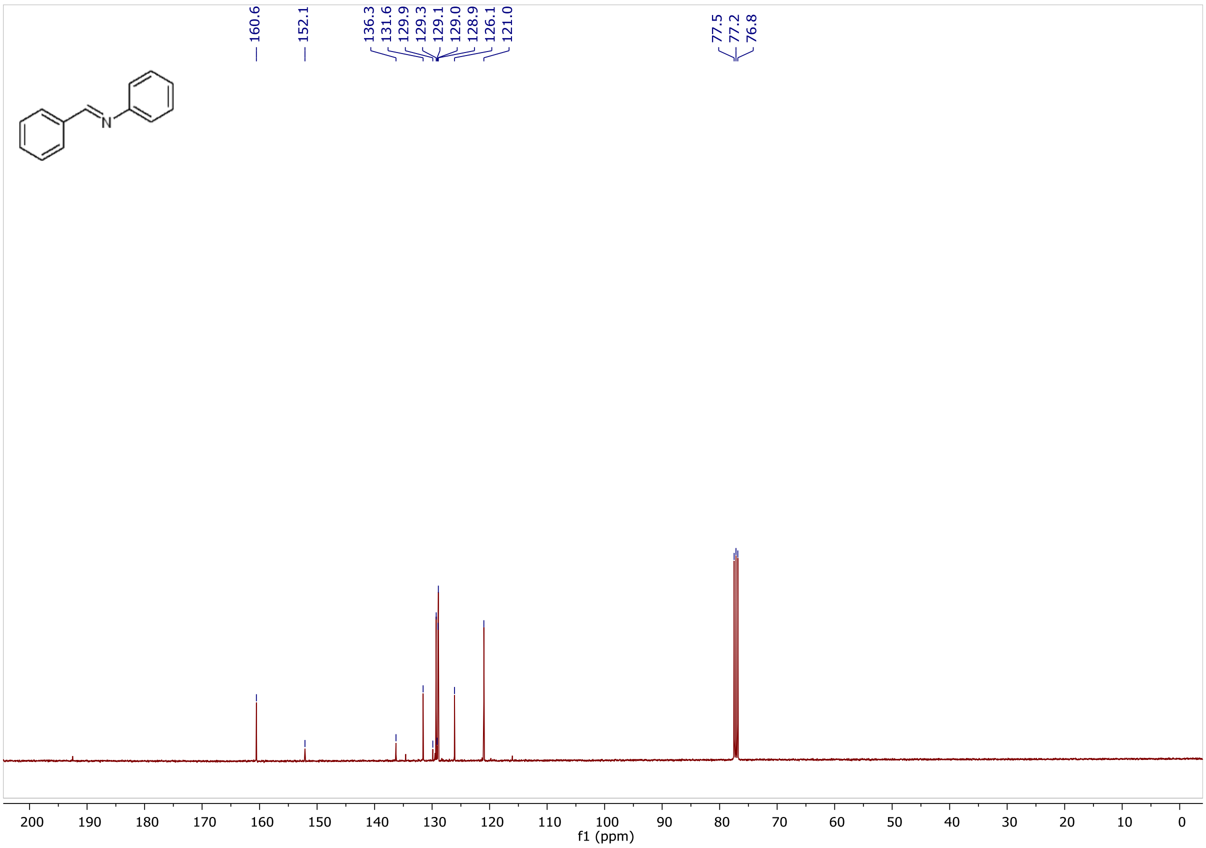
160.6
152.1
136.3
131.6
129.9
129.3
129.1
129.0
128.9
126.1
121.0
77.5
77.2
76.8
N
200 190 180 170 160 150 140 130 120 110 100 90 80 70 60 50 40 30 20 10 0
f1 (ppm)

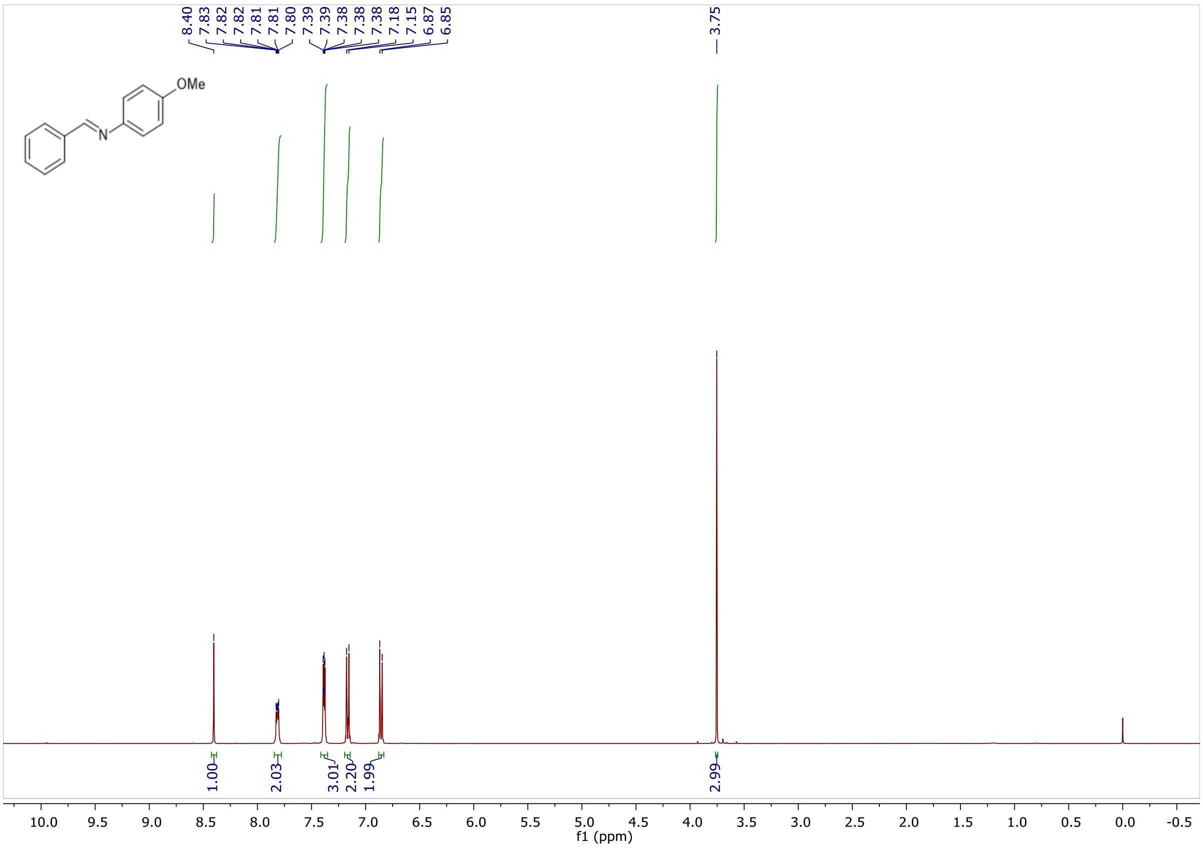
8.40
7.83
7.82
7.82
7.81
7.81
7.80
7.39
7.39
7.38
7.38
7.38
7.38
7.18
7.15
6.87
6.85
3.75
OMe
N
1.00
2.03
3.01
2.20
1.99
2.99
10.0
9.5
9.0
8.5
8.0
7.5
7.0
6.5
6.0
5.5
5.0
4.5
4.0
3.5
3.0
2.5
2.0
1.5
1.0
0.5
0.0
-0.5
f1 (ppm)

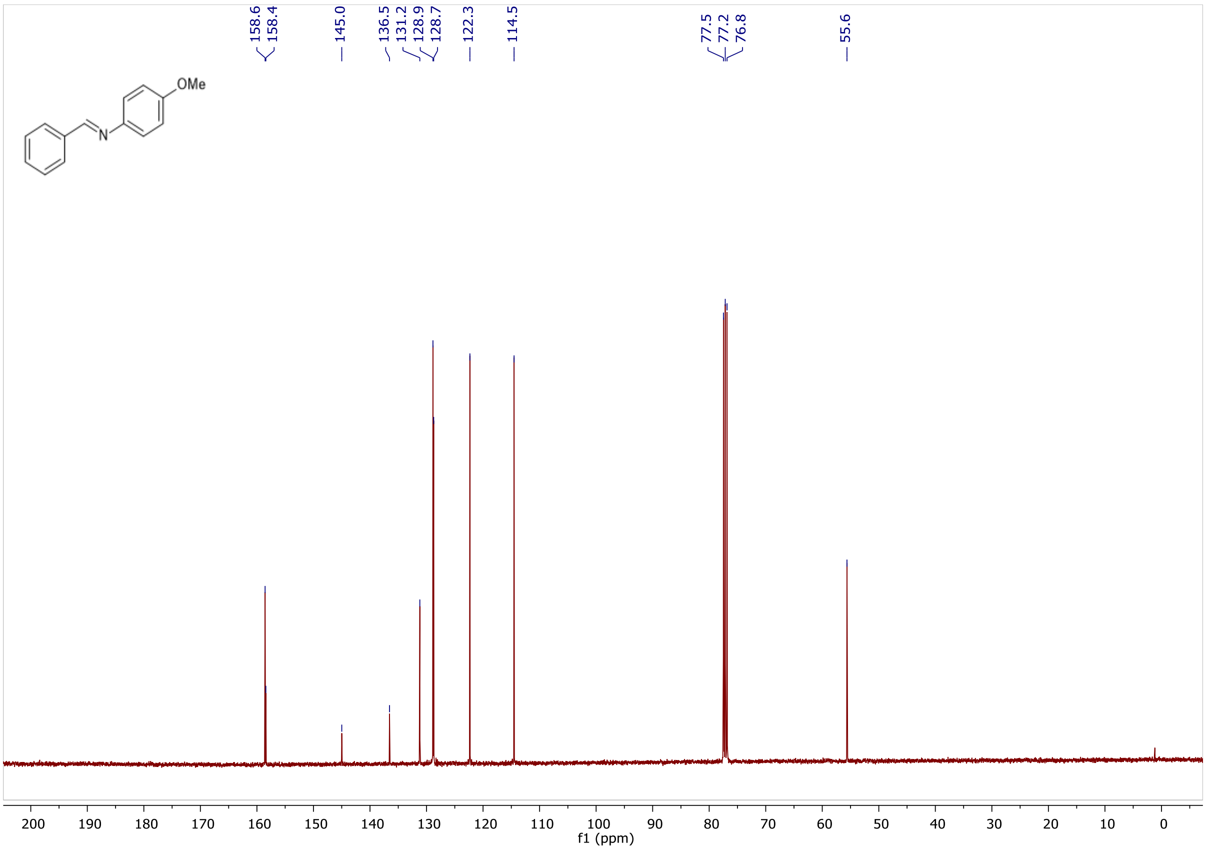
158.6
158.4
145.0
136.5
131.2
128.9
128.7
122.3
114.5
77.5
77.2
76.8
55.6
OMe
N
200
190
180
170
160
150
140
130
120
110
100
90
80
70
60
50
40
30
20
10
0
f1 (ppm)

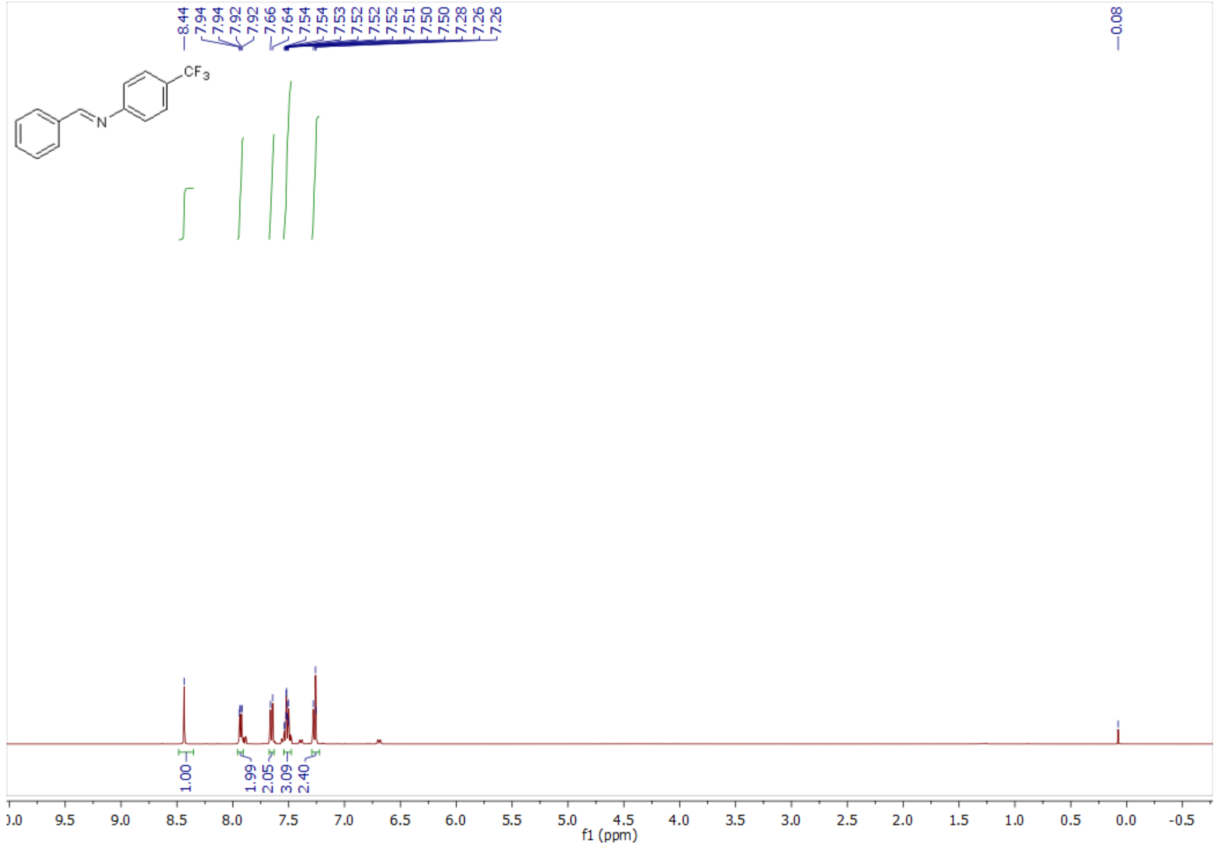
8.44
7.94
7.94
7.92
7.92
7.66
7.64
7.54
7.54
7.53
7.52
7.52
7.52
7.51
7.50
7.50
7.28
7.26
7.26
0.08
CF3
N
1.00
1.99
2.05
3.09
2.40
f1 (ppm)

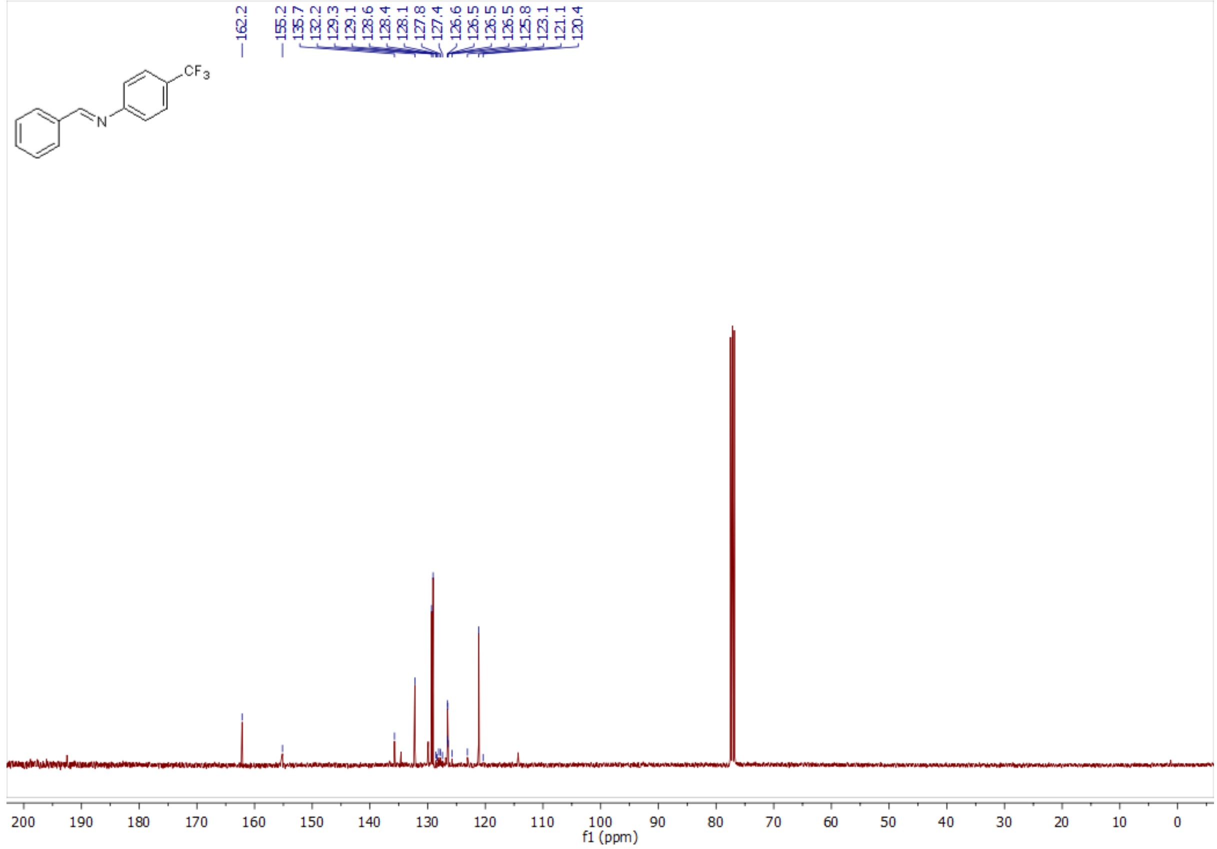
162.2
155.2
135.7
132.2
129.3
129.1
128.6
128.4
128.1
127.8
127.4
126.6
126.5
126.5
126.5
125.8
123.1
121.1
120.4
CF3
N
f1 (ppm)

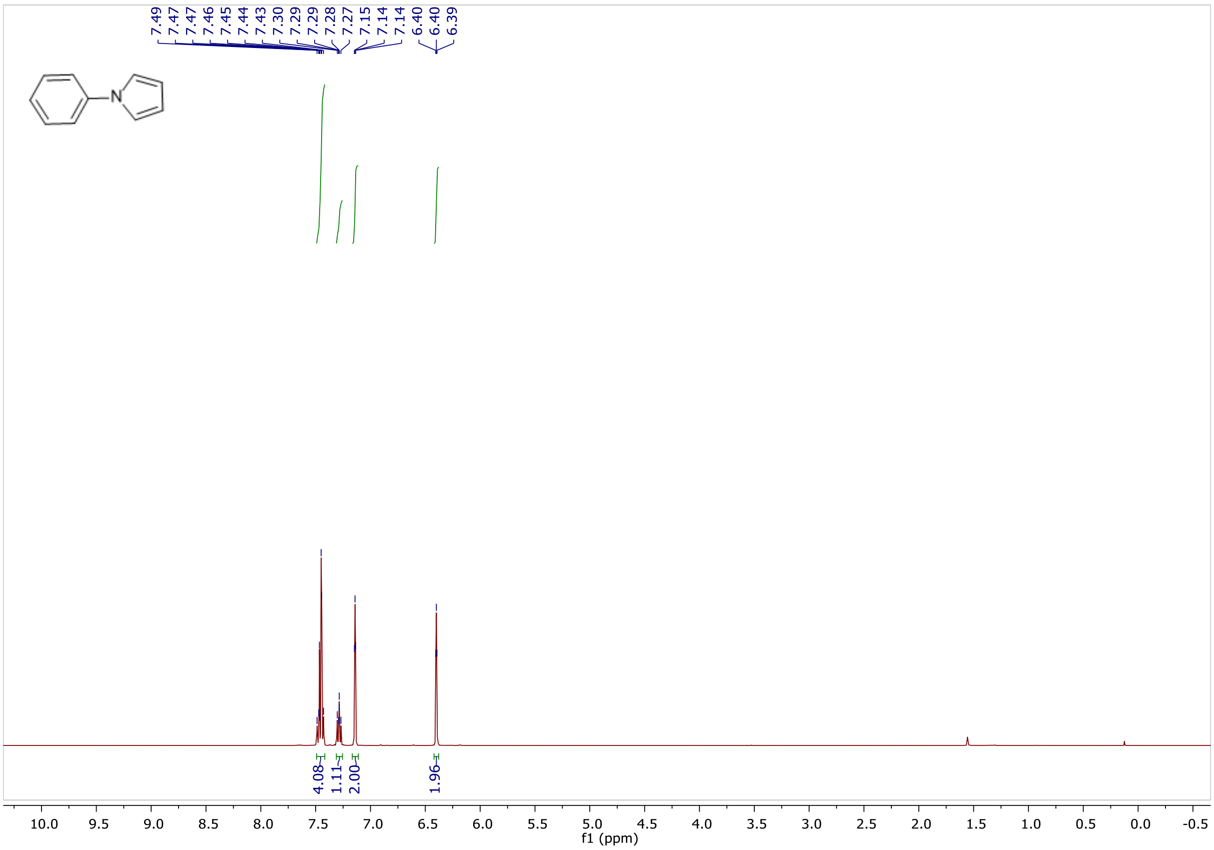
7.49
7.47
7.47
7.46
7.45
7.44
7.43
7.30
7.29
7.29
7.28
7.27
7.15
7.14
7.14
6.40
6.40
6.39
N
4.08
1.11
2.00
1.96
10.0 9.5 9.0 8.5 8.0 7.5 7.0 6.5 6.0 5.5 5.0 4.5 4.0 3.5 3.0 2.5 2.0 1.5 1.0 0.5 0.0 -0.5
f1 (ppm)

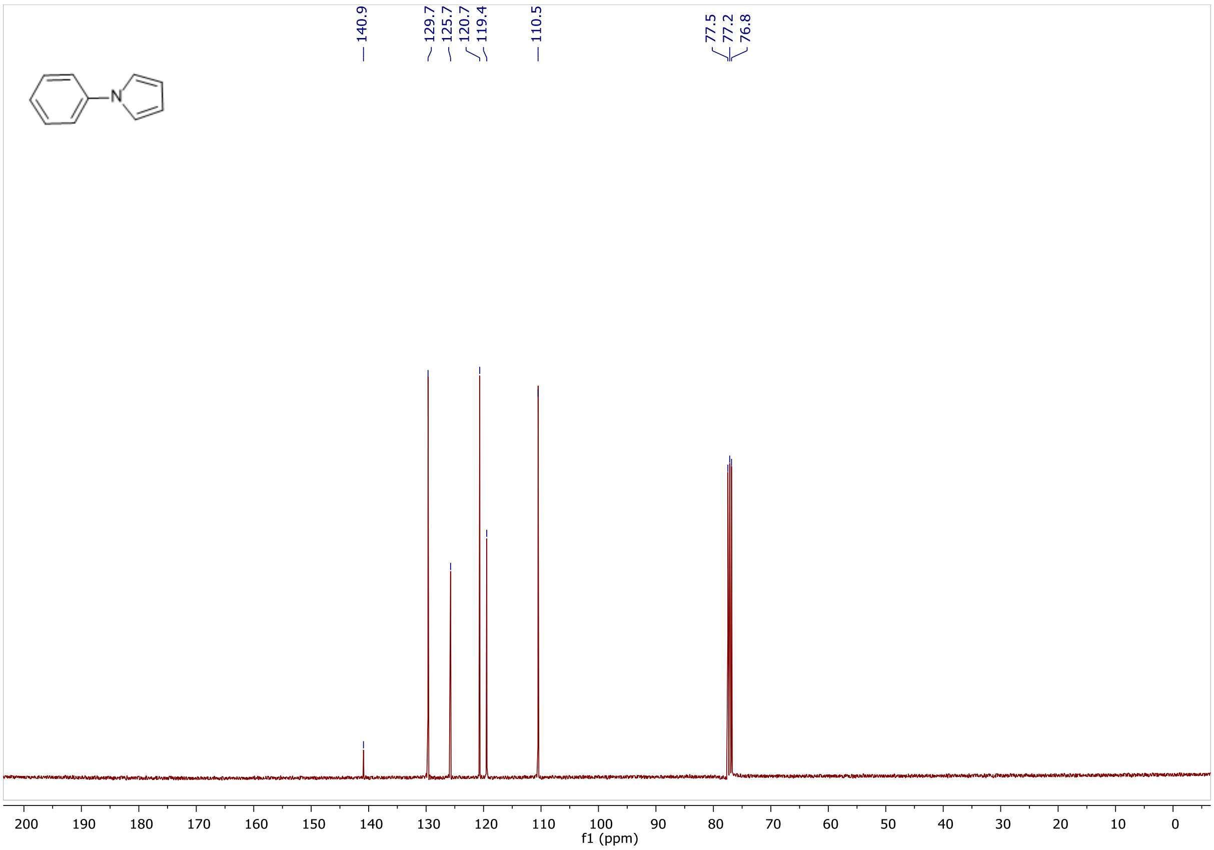
140.9
129.7
125.7
120.7
119.4
110.5
77.5
77.2
76.8
N
200 190 180 170 160 150 140 130 120 110 100 90 80 70 60 50 40 30 20 10 0
f1 (ppm)

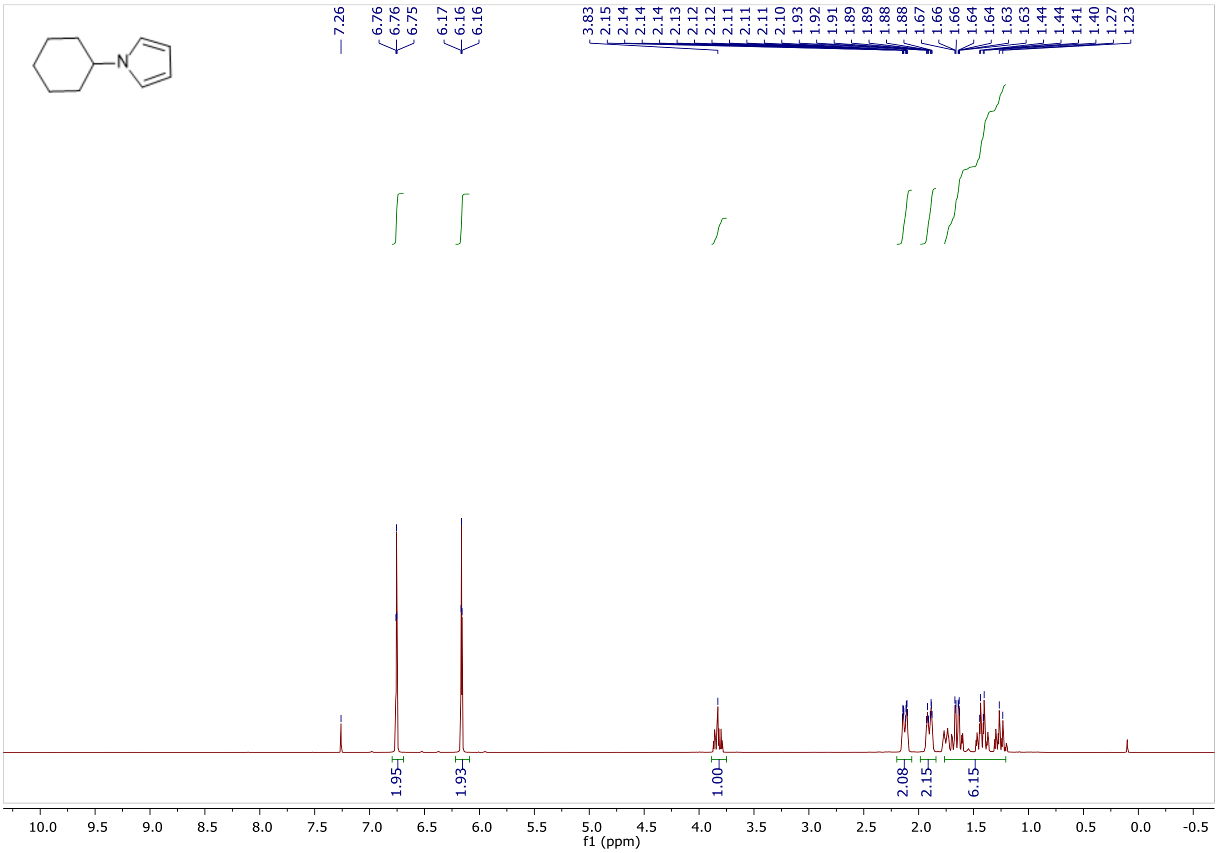
N
7.26
6.76
6.76
6.75
6.17
6.16
6.16
3.83
2.15
2.14
2.14
2.14
2.13
2.12
2.12
2.11
2.11
2.11
2.10
1.93
1.92
1.91
1.89
1.89
1.88
1.88
1.67
1.66
1.66
1.64
1.64
1.63
1.63
1.44
1.44
1.41
1.40
1.27
1.23
1.95
1.93
1.00
2.08
2.15
6.15
10.0 9.5 9.0 8.5 8.0 7.5 7.0 6.5 6.0 5.5 5.0 4.5 4.0 3.5 3.0 2.5 2.0 1.5 1.0 0.5 0.0 -0.5
f1 (ppm)

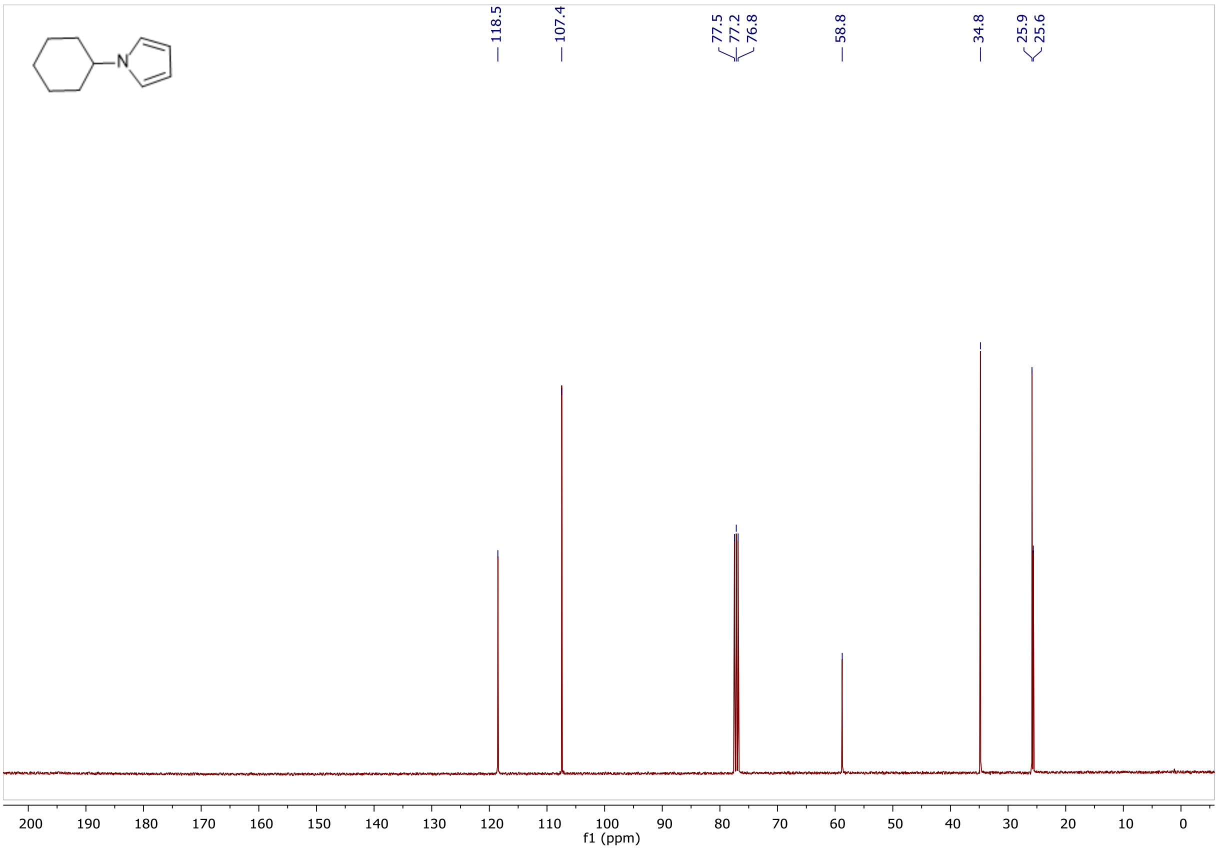
N
118.5
107.4
77.5
77.2
76.8
58.8
34.8
25.9
25.6
200 190 180 170 160 150 140 130 120 110 100 90 80 70 60 50 40 30 20 10 0
f1 (ppm)
